# Metabolic shifts during coffee consumption refresh the immune response: insight from comprehensive multiomics analysis

**DOI:** 10.1002/mco2.617

**Published:** 2024-06-17

**Authors:** Pinglang Ruan, Ming Yang, Xinyi Lv, Kai Shen, Yiran Chen, Hongli Li, Di Zhao, Jianhua Huang, Yang Xiao, Weijun Peng, Haijing Wu, Qianjin Lu

**Affiliations:** ^1^ Department of Dermatology The Second Xiangya Hospital, Central South University Hunan Key Laboratory of Medical Epigenomics Changsha China; ^2^ Hospital for Skin Diseases Institute of Dermatology Chinese Academy of Medical Sciences and Peking Union Medical College Nanjing China; ^3^ Department of Integrated Traditional Chinese and Western Medicine The Second Xiangya Hospital Central South University Changsha China; ^4^ Hunan Academy of Chinese Medicine Hunan University of Chinese Medicine Changsha China; ^5^ National Clinical Research Center for Metabolic Diseases The Second Xiangya Hospital, Central South University Changsha China; ^6^ Key Laboratory of Basic and Translational Research on Immune‐Mediated Skin Diseases Chinese Academy of Medical Sciences Nanjing China; ^7^ Jiangsu Key Laboratory of Molecular Biology for Skin Diseases and STIs Institute of Dermatology Chinese Academy of Medical Sciences and Peking Union Medical College Nanjing China

**Keywords:** anti‐immunosenescence, anti‐inflammatory, coffee consumption, coffee‐related metabolism, immune remodeling

## Abstract

Coffee, a widely consumed beverage, has shown benefits for human health but lacks sufficient basic and clinical evidence to fully understand its impacts and mechanisms. Here, we conducted a cross‐sectional observational study of coffee consumption and a 1‐month clinical trial in humans. We found that coffee consumption significantly reshaped the immune system and metabolism, including reduced levels of inflammatory factors and a reduced frequency of senescent T cells. The frequency of senescent T cells and the levels of the senescence‐associated secretory phenotype were lower in both long‐term coffee consumers and new coffee consumers than in coffee nondrinking subjects, suggesting that coffee has anti‐immunosenescence effects. Moreover, coffee consumption downregulated the activities of the The Janus kinase/signal transduction and activator of transcription (JAK/STAT) and mitogen‐activated protein kinases (MAPK) signaling pathways and reduced systemic proinflammatory cytokine levels. Mechanistically, coffee‐associated metabolites, such as 1‐methylxanthine, 3‐methylxanthine, paraxanthine, and ceramide, reduced the frequency of senescent CD4^+^CD57^+ ^T cells in vitro. Finally, in vivo, coffee intake alleviated inflammation and immunosenescence in imiquimod‐induced psoriasis‐like mice. Our results provide novel evidence of the anti‐inflammatory and anti‐immunosenescence effects of coffee, suggesting that coffee consumption could be considered a healthy habit.

## Graphical abstract

Coffee, a widely consumed beverage, has shown benefits for human health but lacks sufficient basic and clinical evidence to fully understand its impacts and mechanisms. In this study, our findings provide novel evidence of coffee in the anti‐inflammatory and anti‐immunosenescence effects, suggesting that coffee consumption could be considered as one healthy suggestion for healthcare.

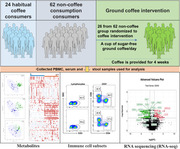

## 1 INTRODUCTION

Coffee, a commonly consumed beverage, has been reported to have beneficial effects on human health. However, the exact mechanism by which it influences health remains incompletely understood.^1^ Previous studies have demonstrated that coffee consumption reduced mortality in patients with inflammatory diseases.^2^, ^3^ The consumption of ≥2 cups of coffee per day has been reported to show a reduction in mortality risk by 30%.^4^

Coffee consumption has been shown the capacity of reducing the risk of Parkinson's disease, Alzheimer's disease, obesity,^5^, ^6^ and type 2 diabetes^7^ due to its anti‐inflammatory and antioxidant properties. It also shows a protective effect against the development of ulcerative colitis, primary sclerosing cholangitis, and multiple sclerosis.^8^ Caffeine can elevate cyclic adenosine monophosphate levels to suppress the secretion of interferon‐gamma (IFN‐γ) and tumor necrosis factor‐alpha (TNF‐α). Moreover, a study demonstrated that caffeic acid can downregulate inflammatory cytokine levels (TNF‐α, interleukin‐6 [IL‐6], etc.),^9^ indicating that coffee consumption has anti‐inflammatory effects.

Several studies have described an association between coffee intake and immune cells. Caffeine supplementation ameliorates experimental allergic encephalomyelitis (EAE) by reducing the frequency and number of Th17 cells. However, consumption of excessive sugar without caffeine promoted Th17 responses and exacerbated inflammation in a mouse model of EAE.^10^ Coffee extract enhances natural killer (NK) cell activity and monocyte phagocytosis and inhibits monocyte chemoattractant protein 1 (MCP‐1)‐induced monocyte migration.^11^ However, the complete molecular mechanism underlying the association between coffee consumption and immune remodeling remains unclear.

It is therefore worth investigating the immunological and metabolic effects of coffee consumption. In this study, we analyzed the effects of coffee consumption on metabolism and the immune response, revealing the potential biological mechanisms underlying immune remodeling. Furthermore, our results highlighted the potential anti‐inflammatory and anti‐immunosenescence effects of ground coffee consumption, providing more evidence for a better understanding of the benefits of coffee to healthcare.

## 2 RESULTS

### 2.1 The adaptive immune cell and metabolomic landscape after habitual coffee consumption

**FIGURE 1:**
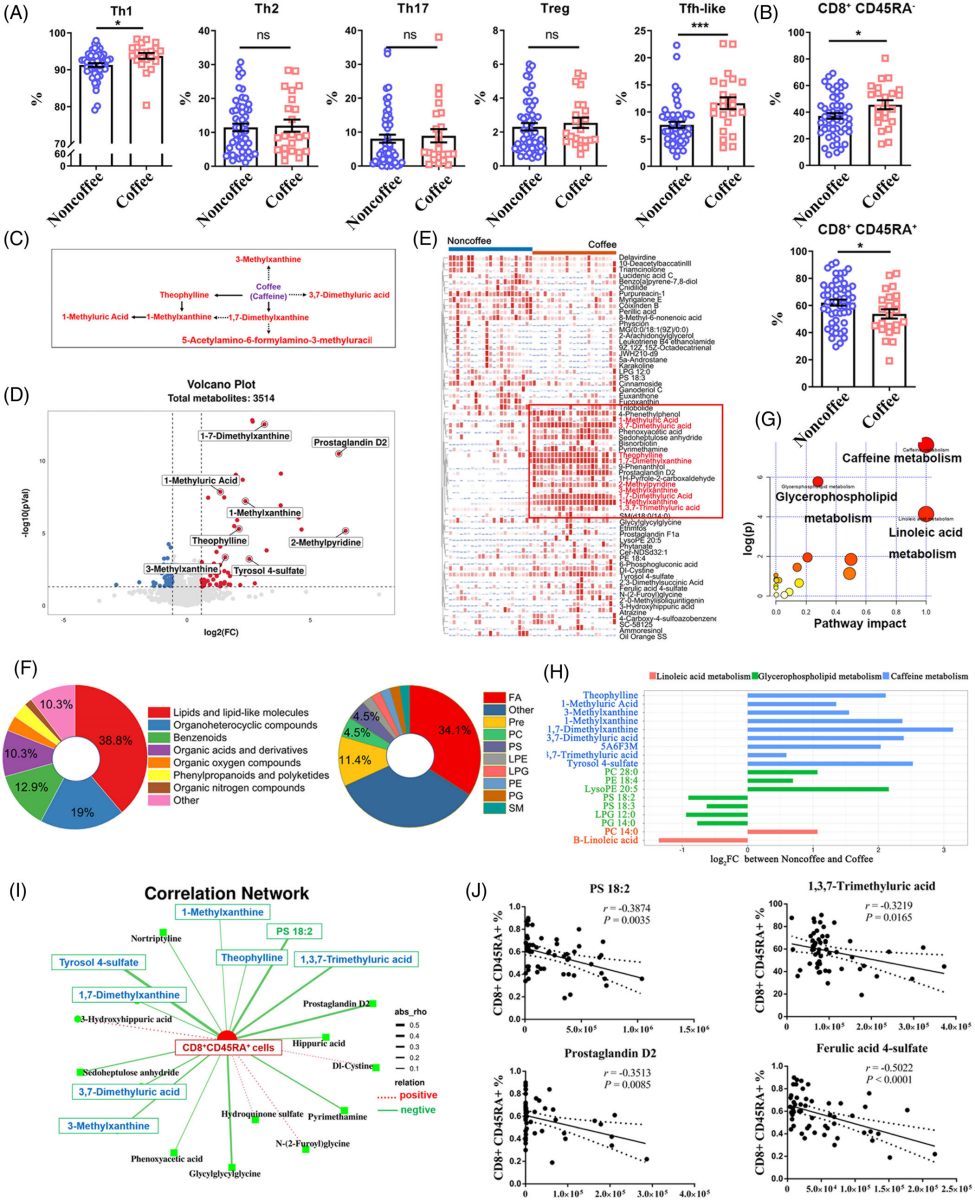
The impacts of habitual coffee consumption on immune cells and serum metabolism. (A) Statistical analysis of the frequency of each CD4^+^ T‐cell subgroup among peripheral blood mononuclear cells (PBMCs) from coffee nonconsumers (*n* = 62) and habitual coffee consumers (*n* = 24). Th1 (CD4^+^CXCR3^+^CCR6^−^CCR4^−^), Th2 (CD4^+^CXCR3^−^CCR6^−^CCR4^+^), Th17 (CD4^+^CXCR3^−^CCR6^+^CCR4^+^), Treg (CD4^+^CD25^+^CD127^−^), and Tfh‐like (CD4^+^CXCR5^+^PD‐1^+^) cells. ^*^ *p *< 0.05; ^***^ *p* < 0.001; ns: no significance. Bars represent the means ± standard errors of the mean (SEMs). (B) Analysis of the frequencies of CD8^+^CD45RA^+^ and CD8^+^CD45RA^−^ T cells. (C) Metabolism of caffeine metabolites found in habitual coffee consumers. Red font indicates increased abundance. (D) Volcano plot of identified differentially abundant metabolites between habitual coffee consumers and coffee nonconsumers, including both the negative electrospray ionization (ESI^−^) and positive electrospray ionization (ESI^+^) modes. Log_2_FC = 1.5, *p* < 0.05. (E) Heatmap showing the differential metabolite abundances between habitual coffee consumers and coffee nonconsumers. Red indicates the metabolite abundances. FA: fatty acids; LPE: LysoPE; LPG: LysoPG; PC: phosphorylcholine; PE: phosphatidylethanolamine; PG: phosphatidylglycerol; Pre: prenol lipids; PS: phosphatidylserine; SM: sphingomyelin. (F) The numbers and proportions of 117 identified metabolites (left) and lipid and lipid‐like molecule subclasses (right) between habitual coffee consumers and coffee nonconsumers. The “other” group of lipid and lipid‐like molecule subclasses included steroids and steroid derivatives, endocannabinoids, glycerolipids, and other undefinable metabolites according to the human metabolome database. (G) KEGG metabolic pathway analysis between habitual coffee consumers and coffee nonconsumers. *p* < 0.05. (H) Analysis of pathway‐based metabolite shifts between habitual coffee consumers and coffee nonconsumers. (I and J) Correlation analysis between CD8^+^CD45RA^+^ cell frequency and metabolite abundances with significant differences.

Several studies have reported that coffee has anti‐inflammatory effects that can reduce the serum levels of inflammatory cytokines.^12^ However, the impact of coffee consumption on immune responses remains uncertain. Initially, we investigated the effects of coffee intake on B‐cell and T‐cell subsets in the peripheral blood of habitual coffee consumers and coffee nonconsumers. Most subsets did not show differential variations in coffee consumers except for an increased frequency of Tfh‐like and Th1 cells (Figures 1A, S1, S2, and S3). Additionally, our findings revealed that the frequency of CD8^+^CD45RA^+^ T cells was reduced in habitual coffee consumers (Figure 1B).

We then conducted a comprehensive metabolomic analysis between habitual coffee consumers and coffee nonconsumers. A total of 117 differentially abundant metabolites (DAMs) were identified, with 67 more abundant metabolites and 50 less abundant metabolites in the habitual coffee consumer group than in the coffee nonconsumer group (Figure 1C,D and Table S1). Then, we visualized the relative abundances of the metabolites using a heatmap (Figure 1E). Notably, caffeine‐related metabolites, including 1‐methylxanthine (1‐MX), 1‐methyluric acid (1‐MUA), 3‐methylxanthine (3‐MX), 1,3,7‐trimethyluric acid (1,3,7‐TMUA), 3,7‐dimethyluric acid (3,7‐DMUA), theophylline (TP), 1,7‐dimethylxanthine (1,7‐DMX), and 2‐methylpyridine, exhibited significantly greater levels in habitual coffee consumers (Figure S4). Furthermore, we performed subclass analysis on 117 DAMs to gain deeper insights into the functional categories to which they belong (Figure 1F). Enrichment analysis revealed that many of these metabolites belong to the lipid and lipid‐like subclass category (38.8%), as well as to the organoheterocyclic compounds subclass category (19%). Fatty acids (34.1%) and prenol lipids (11.4%) were the top two subgroups in the lipid and lipid‐like molecules category.

We performed KEGG metabolic pathway analysis on 117 DAMs. These DAMs were primarily enriched in the caffeine metabolism, glycerophospholipid metabolism, and linoleic acid metabolism pathways (Figure 1G,H and Table S2). We investigated the associations between CD8^+^CD45RA^+^ cells and DAMs (Table S3). The results indicated that 1,3,7‐TMUA, ferulic acid 4‐sulfate, prostaglandin D2, and phosphatidylserine (PS) 18:2 were associated with a decrease in the frequency of CD8^+^CD45RA^+^ cells (Figure 1I,J). 1,3,7‐TMUA, a major metabolite of coffee,^13^ is a potent antioxidant that is effective at scavenging hydroxyl radicals and inhibiting lipid peroxidation,^14^ which are associated with immunosenescence.^15^ Ferulic acid 4‐sulfate, a ferulic acid (FA), is also a major coffee metabolite with antioxidant and anti‐inflammatory effects.^16^, ^17^ FA supplementation can effectively delay immunosenescence. Therefore, it is widely used as an antiphotoaging agent in skin,^18^, ^19^ but its molecular mechanism remains unclear. Abundances of PS, a lipid metabolite, increased significantly in habitual coffee consumers. Research has reported that adding PS to the diet can reduce changes in cognitive function caused by aging and neuroinflammation.^20^

### 2.2 Anti‐inflammatory and anti‐immunosenescent effects of ground coffee intake for 28 days

A recent study demonstrated that caffeinated coffee has significant impacts on obesity, cardiovascular health,^21^ and acute health effects (including arrhythmia, daily steps, and sleep time).^22^ In this study, we recruited 26 coffee nonconsumers to consume freshly ground unsweetened black coffee for 28 days (1 cup/day, approximately 1 ounce/cup). At the endpoint of the trial, no significant differences in the body weights of the participants compared to their weights before the initiation of the trial were observed (Table 1). Our findings revealed that during the postintervention period, the total time the participants spent in bed slightly increased by an average of 5.42 min per night. However, the average total sleep time during the postintervention period was 436.3 min per night (441.3 min preintervention, for a decrease of approximately 5 min per night) (Table 2). Our results indicate that coffee intake does not significantly affect the prolongation of sleeping time.

**TABLE 1:** Weight changes in the total cohort.

	Ground coffee (*n* = 26)		
	Preintervention	Postintervention	*p*‐Value
iHealth weight (kg)	57.04 (54.13, 59.95)	54.65 (53.79, 59.74)	Ns
Body mass index	21.08 (20.18, 21.98)	20.98 (20.03, 21.92)	Ns

*Note*: The data are represented as the mean (95% confidence interval).

Abbreviation: ns, not significant.

**TABLE 2:** Self‐reported sleep measures.

	Ground coffee (*n* = 26)		
	Preintervention	Postintervention	*p‐*Value
RED scale score	21.60 (20.37, 22.82)	21.27 (20.04, 22.49)	Ns
PSQI score	6.47 (5.24, 7.69)	6.69 (5.55, 7.84)	Ns
Total time in bed (min)	28.96 (23.19, 34.73)	34.38 (28.33, 40.42)	Ns
Total sleep time (min)	441.3 (426.6, 455.9)	436.3 (421.3, 451.2)	Ns

*Note*: When scoring the PSQI, seven different scores are calculated for each component, ranging from 0 (no issues) to 3 (significant issues). The individual scores are added together to create an overall score that falls between 0 and 21. Poorer sleep quality is indicated by higher scores. For the RED scale, participants read statements about food and eating. On a scale of 1—“Not at all like me” to 5—“Exactly like me,” participants rated to what extent they agreed with the statement. Scores range from 9 to 45; higher scores indicate greater reward‐based eating drive. The data are represented as the mean (95% confidence interval).

Abbreviations: ns, not significant; PSQI, Pittsburg Sleep Quality Index; RED scale, Rewards‐based Eating Drive Scale.

**FIGURE 2:**
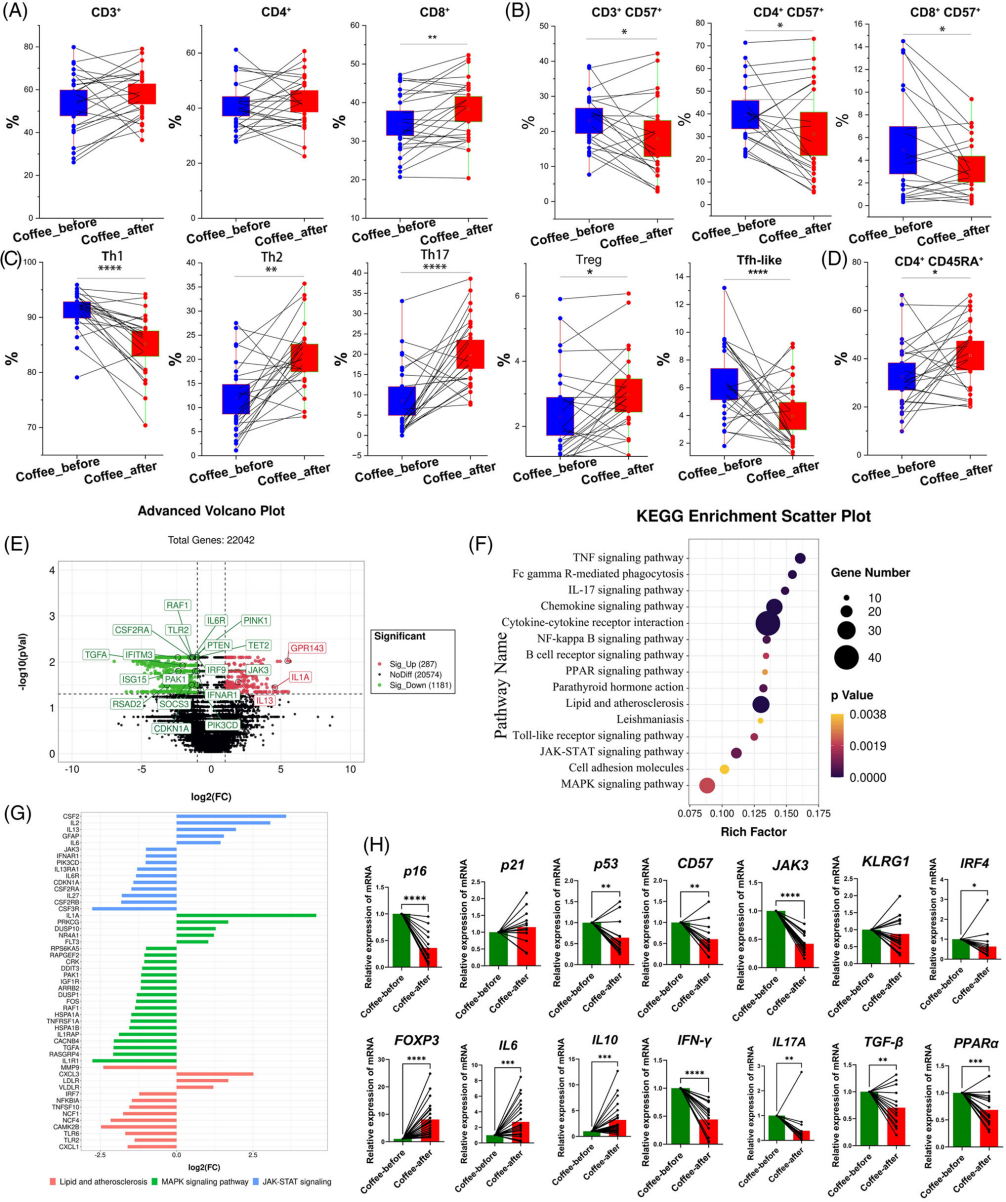
The effects of ground coffee consumption on adaptive immune cells. (A‒D) Statistical analysis of each CD4^+^ T‐cell subgroup among peripheral blood mononuclear cells (PBMCs) obtained from 26 volunteers. Boxes represent the dates, and lines connecting dates show the data from one subject. (E) Volcano plot of differentially expressed genes (DEGs) identified in PBMCs collected from five individuals both before and after consuming ground coffee. *n* = 5. Fold change value = ±1, *p* < 0.05. (F) KEGG enrichment analyses of DEGs in subjects after ground coffee consumption. (G) Pathway‐based analysis of mRNA expression in subjects after ground coffee consumption. (H) The mRNAs expression of *p16*, *p21*, *p53*, *CD57*, *JAK3*, *KLRG1*, *IRF4*, *FOXP3*, *IL6*, *IL10*, *IFN‐γ*, *IL17A*, *TGF‐β*, and *PPARα*. *n* = 26. Green: before coffee; red: after coffee; ^*^ *p* < 0.05; ^**^ *p* < 0.01; ^***^ *p* < 0.001; ^****^ *p* < 0.0001.

After ground coffee consumption, the frequency of total CD8^+^ T cells increased (Figure 2A). We found a significant decrease in the frequency of CD57^+^ T cells, including CD3^+^CD57^+^, CD4^+^CD57^+^, and CD8^+^CD57^+^ cells (Figure 2B), which are senescent T cells.^23^ However, except for the reduced frequency of CD57^+^ Treg cells, no significant difference was found in the frequency of other CD57^+^ Th cell subsets (Figure S5). In addition, after ground coffee consumption, the frequencies of Treg cells (CD25^hi^CD127^lo^), Th2 cells and Th17 cells increased, while the frequencies of Th1, Tfh‐like, and CD4^+^CD45RA^+^ T cells decreased (Figure 2C,D). Furthermore, there was no significant difference in the frequency of CD8^+^CD45RA^+^ T cells (Figure S6A). Our results provide evidence for the effects on anti‐immunosenescence from coffee consumption.

The frequencies of NK cells (CD56^+^ cells) and antibody‐secreting cells (ASCs, CD19^+^CD27^hi^CD38^hi^) increased after ground coffee consumption. Additionally, the frequency of (double positive B cells (DPB), CD19^+^IgD^+^CD27^+^) was lower following ground coffee consumption. However, no significant differences were detected in the frequencies of other B‐cell subsets, such as switched memory B cells (CD19^+^IgD^−^CD27^+^), (double negative B cells (DNB) cells, CD19^+^CD27^−^IgD^−^), and naïve B cells (CD19^+^IgD^+^CD27^−^), after ground coffee consumption (Figure S6B).

### 2.3 Downregulation of inflammatory signaling pathways after 28 days of ground coffee consumption

We next focused on transcriptomic alterations that occurred following coffee consumption. A total of 1468 differentially expressed genes (DEGs) were found after ground coffee consumption (Figure 2E). GO analysis revealed that many DEGs were enriched mainly in the pathways of IL‐10 production and T‐cell differentiation (Table S4), and KEGG analysis revealed that the activities of several inflammatory signaling pathways, such as the TNF, IL‐17, lipid metabolism and atherosclerosis processes, the Janus kinase/signal transduction and activator of transcription (JAK/STAT), mitogen‐activated protein kinases (MAPK), nuclear factor‐kappa B and toll‐like receptor pathways, were downregulated (Figure 2F and Table S5). Notably, gene set enrichment analysis revealed a decreased risk of oxidative phosphorylation, Parkinson's disease, Alzheimer's disease, Huntington's disease, and type 1 diabetes after coffee consumption (Figure S7A and Table S6).

The data from RNA‐seq revealed that the expression of several proinflammatory genes and senescence‐associated secretory phenotype genes (e.g., *CDKN1A*, *IL6R*, *ISG15*, *IRF9*, *TGFA*, *IFNAR1*, *CSF2RA*, *RAF1*, *IFITM3*, *SOCS3*, *PIK3CD*, and *JAK3*) significantly decreased in ground coffee consumers (Figures 2G and S7B). Consistent with the RNA‐seq results, the mRNA expression levels of *p16*, *p53*, *CD57*, *BAX*, *IFNγ*, *IL17A*, *IRF4*, *JAK3*, *TGFB*, and *PPAR*α were downregulated, while levels of *Foxp3*, *IL6*, and *IL10* were significantly upregulated after coffee consumption (Figure 2H). Similarly, levels of *p21*, *CD57*, *IL17A*, and *IFNγ* were downregulated, and the levels of *IL6* and *IL10* were upregulated in habitual coffee drinkers (Figure S8).

We then investigated the associations between genomic features and metabolic features to explore potential key genetic factors. Using the Recon3D database,^24^ we examined the correlation between the mRNA expression of metabolic genes and the abundances of corresponding polar metabolites. The results showed that most gene‒metabolite pairs exhibited moderate correlations, highlighting the complexity and subtlety of the coffee‐related metabolic networks (Table S7).

### 2.4 The upregulation of ceramide metabolism and lipid metabolism after ground coffee consumption

**FIGURE 3:**
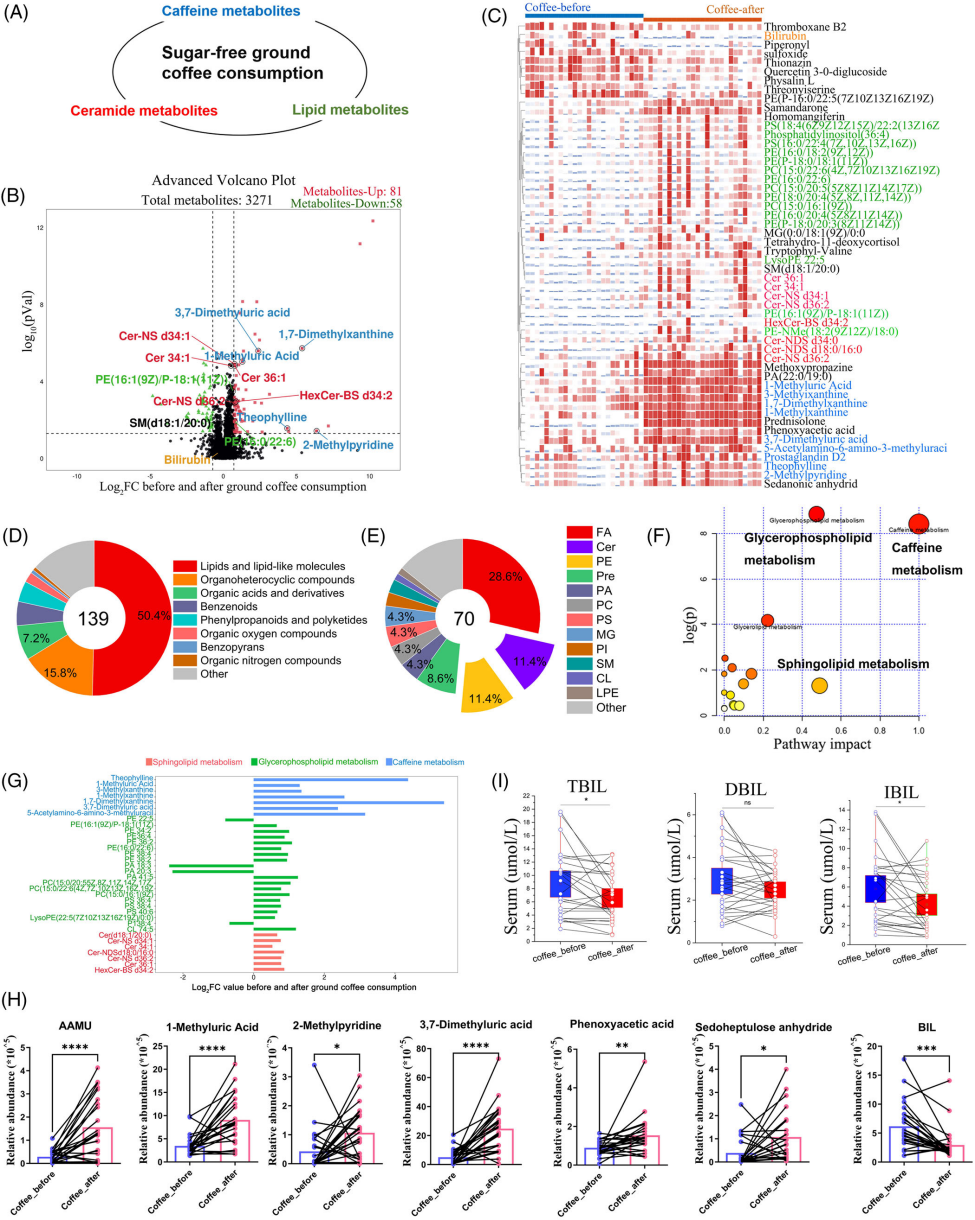
Metabolomic alterations in subjects before and after ground coffee consumption. (A) Unsweetened ground coffee metabolism, including caffeine metabolism, glycerophospholipid metabolism, and sphingolipid metabolism. (B) Volcano plot of identified metabolites in subjects after ground coffee consumption in both the negative electrospray ionization (ESI^−^) and positive electrospray ionization (ESI^+^) modes. *n* = 26. Log_2_‐fold change value = 1.5, *p* < 0.05. (C) Heatmap showing the metabolite abundances in subjects before and after ground coffee consumption. Red indicates the metabolite abundances. FA: fatty acids; LPG: LysoPG; LPE: LysoPE; PC: phosphorylcholine; PE: phosphatidylethanolamine; PG: phosphatidylglycerol; Pre: prenol lipids; PS: phosphatidylserine; SM: sphingomyelin. (D and E) The numbers and proportions of 140 identified metabolites (D) and 70 lipid and lipid‐like molecule subclasses (E) in subjects after ground coffee consumption. (F) KEGG metabolic pathway analysis in subjects after ground coffee consumption. *p* < 0.05. (G) Analysis of pathway‐based metabolite shifts in subjects after ground coffee consumption. (H) Common metabolites between serum samples and stool samples from subjects before and after ground coffee consumption. The data were derived from stool samples. The bars indicate the means ± standard errors of the mean (SEMs). (I) Serum samples from individuals were tested for total bilirubin (TBIL), direct bilirubin (DBIL), and indirect bilirubin (IBIL) levels before and after consuming ground coffee, with the results displayed on the *Y*‐axis in µmol/L. Boxes represent the dates, and lines connecting dates show the data from one subject. ^*^ *p* < 0.05; ^**^ *p* < 0.01; ^***^ *p *< 0.001; ^****^ *p* < 0.0001.

To further reveal metabolic changes related to coffee consumption, we compared the DAMs in subjects before and after ground coffee consumption. Ceramide metabolism and lipid metabolism were significantly upregulated after ground coffee consumption compared with before ground coffee consumption (Figure 3A). Notably, we identified a total of 139 DAMs, including 81 with higher abundances and 58 with lower abundances after ground coffee consumption (Figure 3B and Table S8).

Then, we visualized the abundances of the DAMs after ground coffee consumption via a heatmap (Figure 3C). Among the DAMs, we found that coffee consumption upregulated the abundances of coffee‐associated metabolites (including 1‐MX, 1‐MUA, 3‐MX, 3,7‐DMUA, TP, PX, and 5A6F3M), ceramide subclass metabolites (including Cer 34:1, Cer 36:1, Cer‐NDS d34:0, Cer‐NDS d18:0/16:0, Cer‐NS d34:1, and Cer‐NS d36:2), and lipid subclass metabolites (including phosphatidylethanolamine [PE], phosphorylcholine [PC], PS, and phosphatidylglycerol [PG]) (Figure S9).

Moreover, we conducted subclass analysis of the annotated metabolites (Figure 3D). The DAMs mainly were lipids and lipid‐like molecules (50.4%), and fewer metabolites involved were organoheterocyclic compounds (15.8%) after ground coffee consumption. Among the lipid and lipid‐like molecule classes, fatty acids (28.6%), PEs (11.4%), and ceramide metabolites (11.4%) were the three main subclasses (Figure 3E).

Furthermore, KEGG metabolic pathway analysis revealed that the DAMs were significantly enriched in caffeine metabolism, glycerophospholipid metabolism, and sphingolipid metabolism (Figure 3F), and the abundances of their metabolites significantly increased after ground coffee consumption (Figure 3G). We also explored the metabolite abundances in stool samples before and after ground coffee consumption (Figure S10). A total of 82 metabolite abundances were significantly changed (Table S9). In particular, there were a total of seven metabolites shared between the serum samples and stool samples, including 2‐methylpyridine, 1‐MUA, bilirubin, phenoxyacetic acid, sedoheptulose anhydride, 3,7‐DMUA, and 5‐acetylamino‐6‐amino‐3‐methyluracil (Figure 3H). Several epidemiological studies have described an association between bilirubin and the risk of cardiovascular disease, brain injury, and hypertension.^25^, ^26^ Additionally, we examined the serum levels of total bilirubin, direct bilirubin, and indirect bilirubin, which were significantly reduced after coffee consumption (Figure 3I).

### 2.5 Crucial coffee metabolites downregulated immunosenescence in vitro

**FIGURE 4:**
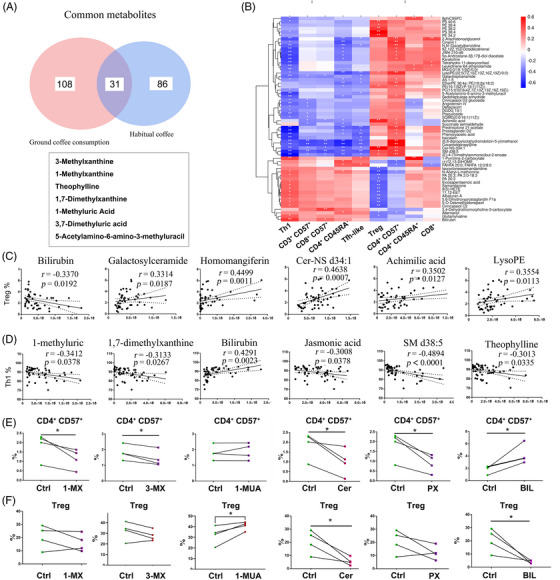
Crucial coffee metabolites downregulated immunosenescence in vitro. (A) Thirty‐one shared metabolites with elevated abundances. (B) Correlation heatmap and correlation network between metabolite abundances and T‐cell frequencies in subjects after ground coffee consumption. (C and D) Associations of ground coffee consumption‐related metabolites with the frequencies of Treg cells (C) and Th1 cells (D). (E and F) Statistical analysis of the frequencies of CD4^+ ^CD57^+^ T cells (E) and Treg cells (F). CD4^+^ T cells were treated with 100 µM 1‐methylxanthine (1‐MX), 100 µM bilirubin, 100 µM paraxanthine (PX), 100 µM 1‐methyluric acid (1‐MUA), 100 µM ceramide mixed metabolites (Cer), or 100 µM 3‐methylxanthine (3‐MX). *n* = 4. ^*^ *p* < 0.05; ^**^ *p* < 0.01.

We found 31 shared metabolites with elevated abundances, 15 in the negative electrospray ionization (ESI^−^) mode and 16 in the positive electrospray ionization (ESI^+^) mode, in the habitual coffee drinker group (including 117 DAMs) and the ground coffee consumption group (including 139 DAMs) (Figures 4A and S11).

To further explore the associations between metabolomics and T‐cell subsets after ground coffee consumption, we identified several important T‐cell‐regulated metabolites (Figure 4B and Table S10). By correlation analysis, we found that the frequency of Treg cells was positively correlated with the serum metabolite abundances (including galactosylceramide, homomangiferin, Cer‐NS d34:1, and achimilic acid) but negatively correlated with the serum abundance of bilirubin. Moreover, the frequency of Th1 cells was negatively correlated with the serum metabolite abundances (including 1‐MUA, PX, jasmonic acid, SM d28:5, and TP) but positively correlated with the serum abundance of bilirubin (Figure 4C,D).

We next investigated whether the downregulation of immunosenescence is mediated by these coffee‐associated metabolites. In light of this difference, we therefore, selected six metabolites, which included four coffee metabolites, ceramide complexes, and bilirubin, to examine their effects in vitro. Treatment of CD4^+^ T cells with different coffee metabolites at 100 µM did not affect cell survival (Figure S12A). We observed that 1‐MX, 3‐MX, paraxanthine (PX), and ceramide mixed metabolites (Cer) administration downregulated the frequency of CD4^+^CD57^+^ T cells, and bilirubin administration upregulated the frequency of CD4^+^CD57^+^ T cells (Figure 4E). Furthermore, the effects of these compounds on each T‐cell subset exhibited variability. Specifically, 1‐MX administration had no significant effect on other T‐cell subsets, while 3‐MX administration only reduced the frequency of CD4^+^ effector T cells. Similarly, 1‐MUA administration selectively increased the frequency of Treg cells. PX administration decreased the frequency of Tfh, CD57^+^ naïve T and CD57^+^ Treg cells, while Cer administration decreased the frequency of Tfh, CD57^+^ Tfh, CD57^+^ Th17 and CD57^+^ naïve T cells. Finally, bilirubin administration increased the frequencies of CD4^+^ effector T, CD57^+^ Tfh, CD57^+^ Th1, and CD57^+^ naïve T cells and decreased the frequency of Treg cells (Figures 4F and S12B–G). These results indicate the anti‐immunosenescent effects on T‐cell subsets from coffee and its metabolites.

### 2.6 Coffee exhibited anti‐inflammatory and anti‐immunosenescence properties in vivo

**FIGURE 5:**
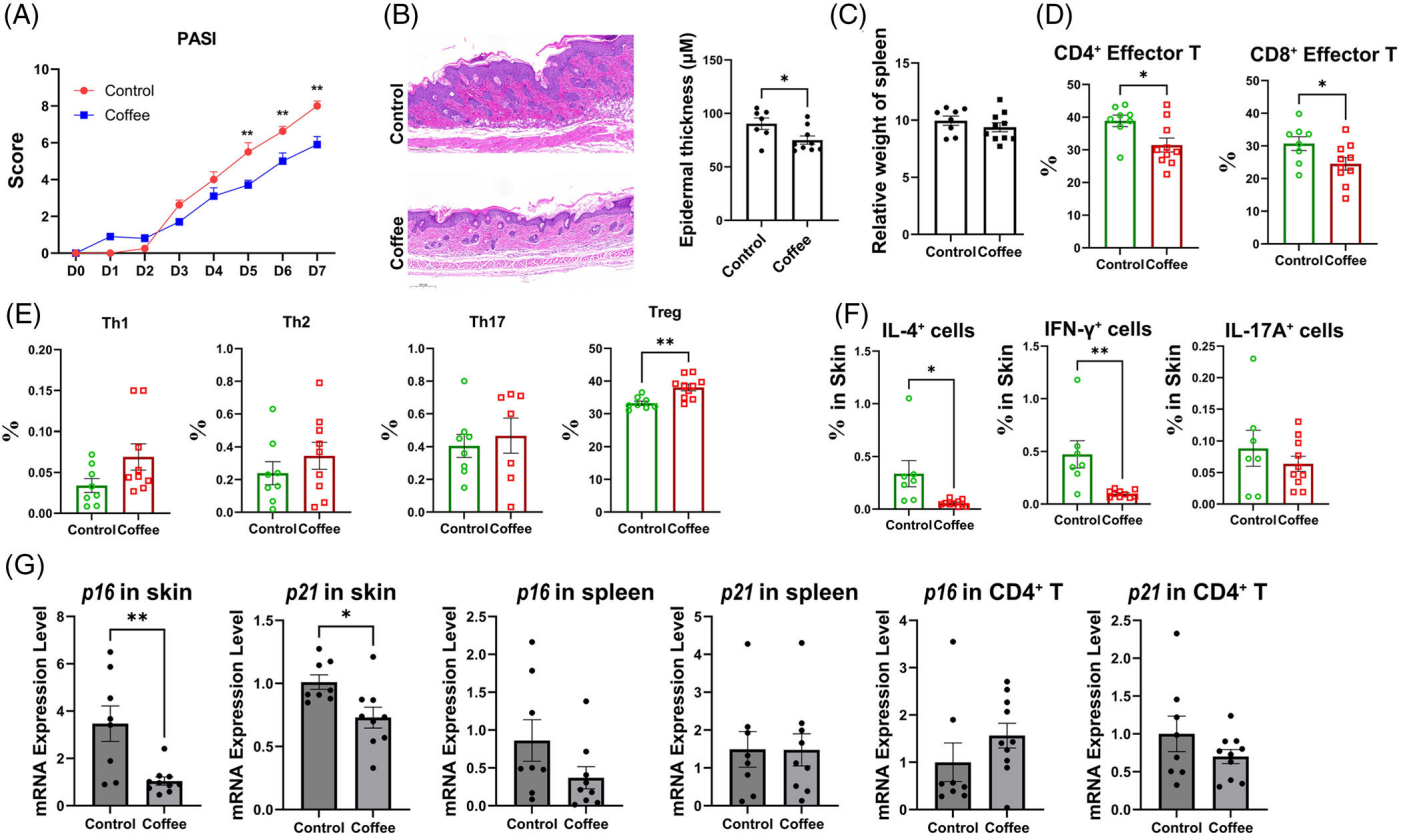
Coffee reduced imiquimod (IMQ)‐induced psoriasis‐like inflammation on mice. (A) Comparison of PASI scores between the control (*n* = 8) and coffee treatment groups (*n* = 10). (B) Examination of lesional H&E staining. (C) Evaluation of spleen weight. (D and E) Frequency analysis of T‐cell subsets in the spleens. (F) Frequency analysis of IL4^+^, IFN‐γ^+^, and IL‐17A^+^ immune cells in the skin. (G) mRNA levels of *p16* and *p21* in skin cells, spleen cells, and CD4^+^ T cells isolated from the spleens. PASI, Psoriasis Area and Severity Index; H＆E, Hematoxylin and eosin. ^*^ *p* < 0.05; ^**^ *p* < 0.01.

**FIGURE 6:**
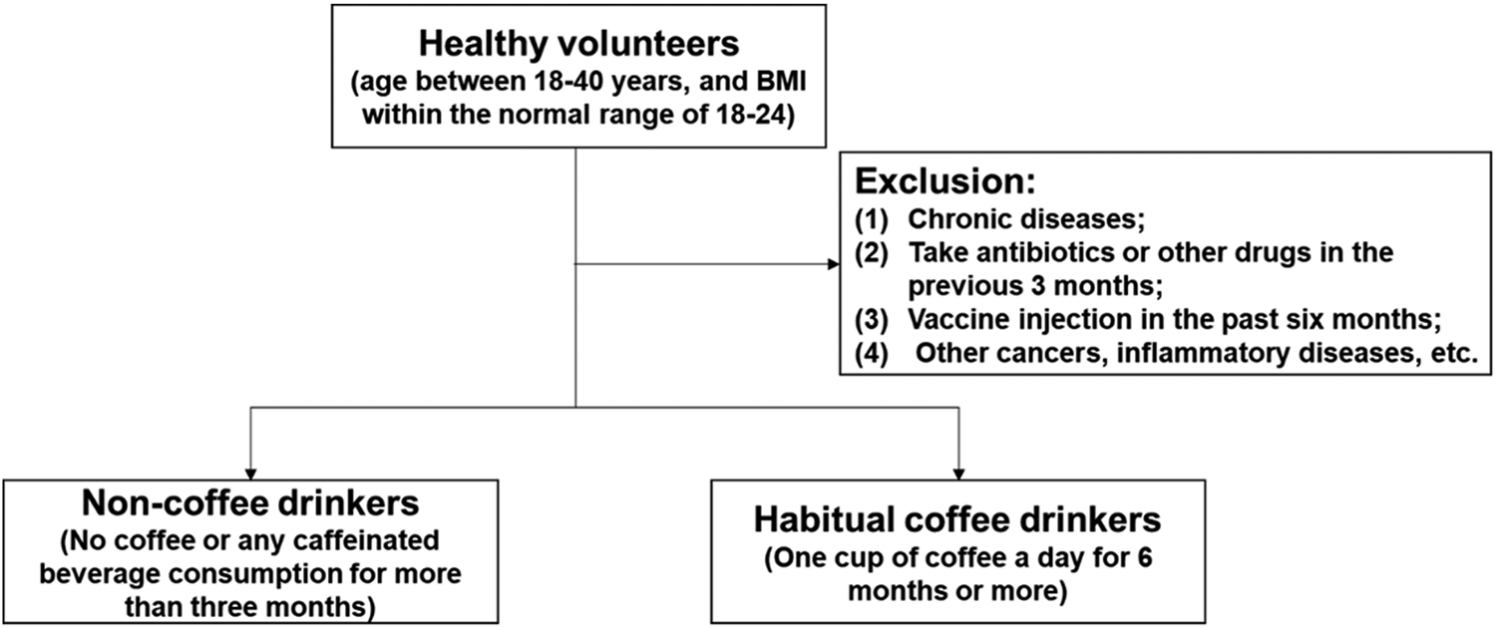
Flowchart depicting the inclusion and exclusion criteria.

To investigate the potential of coffee to alleviate psoriasis‐like skin inflammation, we used ground coffee (130 mg/kg/day) to treat imiquimod (IMQ)‐induced psoriasis‐like skin inflammation in mice. Compared with those in the control mice, the psoriasis‐like lesions in the mice receiving coffee treatment were alleviated (Figures 5A and S13). Additionally, histopathological analysis revealed alleviated epidermal thickening in mice treated with coffee (Figure 5B). Although no significant difference in the spleen index was detected (Figure 5C), a lower frequency of CD4^+^ effector cells and CD8^+^ effector cells and a greater frequency of Treg cells were detected in splenocytes after coffee treatment (Figure 5D,E). Moreover, in the skin tissue of the coffee intervention group, a decreased frequency of IL‐4^+^ and IFN‐γ^+^ immune cells was observed (Figure 5F). Furthermore, the mRNA expression of *p16* and *p21* was lower in the skin tissue of the mice after coffee treatment (Figure 5G). Figures 6

We conducted keyhole limpet haemocyanin (KLH) immunization on mice to observe the effects of coffee on B‐cell responses to antigens (Figure S14). After KLH immunization, the mice that received coffee treatment exhibited an increased frequency of Treg and Tfh cells in the lymph nodes as well as of germinal center B cells in the spleen. Notably, the coffee intervention group exhibited increased levels of total immunoglobulin G (IgG), IgG1, IgG2A, IgG2B, and IgM. This finding suggested that coffee intake promoted a humoral immune response in KLH‐immunized mice. Moreover, the mRNA levels of *p16* and *p21* were reduced in splenocytes and CD4^+^ splenocytes after coffee treatment. These findings indicate that coffee treatment enhances the immune response while reducing immunosenescence in KLH‐immunized mice.

## 3 DISCUSSION

In this current study, we conducted a comprehensive analysis of metabolomic and immune remodeling associated with coffee consumption. Habitual coffee consumers exhibited a decrease in the frequency of CD8^+^CD45RA^+^ cells. Additionally, after ground coffee intake, participants showed an increased frequency of Treg cells and a decreased frequency of CD3^+^CD57^+^ T cells, CD4^+^CD57^+^ T cells, and CD8^+^CD57^+^ T cells. Furthermore, there was a significant reduction in inflammatory pathway activity. Several key metabolites, including PX and 1‐MX, have been demonstrated to effectively reduce the frequency of CD4^+^CD57^+^ T cells in vitro. Moreover, coffee intake reduced the frequency of IL4^+^ and IFN‐γ^+^ immune cells and the mRNA levels of *p16* and *p21* in vivo. Our research provides a detailed overview of the anti‐inflammatory and anti‐immunosenescent effects of coffee and its metabolites.

Our research revealed that primary coffee metabolites were enriched in both habitual coffee consumers and ground coffee intervention consumers. Previous studies confirmed the same results by identifying certain metabolites as potential indicators of coffee intake.^27^, ^28^ Additionally, coffee intake could also impact lipid metabolism.^29^ FAs, PS, prenol lipids, phosphatidylethanolamine, phosphorylcholine, phosphatidylglycerol, and LysoPE were found to be significantly enriched in both habitual coffee consumers and ground coffee intervention consumers. Consistent with our results, increased levels of ceramides were closely linked to coffee consumption.^30^ However, PE and ceramide metabolites were only enriched in the ground coffee intervention group, which may be due to the different types of coffee consumed in these two groups. Our results revealed that the abundance of ceramide metabolites increased after coffee intake, including Cer 34:1, Cer 36:1, Cer‐NDS d34:0, Cer‐NDS d18:0/16:0, Cer‐NS d34:1, and Cer‐NS d36:2. This finding was different from those of previous studies (only three ceramide metabolites were measured, Cer 20:4, Cer 18:1, and Cer 18:2).^29^ Ceramide metabolites increase T cell receptor (TCR) expression.^31^ However, some studies have shown that ceramide metabolites can induce proinflammatory responses,^32^ suggesting the need for ceramide‐specific studies.

Coffee is rich in bioactive compounds such as chlorogenic acid, caffeine, trigonelline, and magnesium, which can boost immunity and reduce inflammation by acting as antioxidants and regulating glycemic and lipid levels.^6^, ^33^ We found that ceramide metabolites and lipid metabolites (such as PE, PS, and were associated with an increased frequency of Treg cells and a decreased frequency of Th1 cells. The inhibition of SMS1 by Foxp3 leads to the accumulation of ceramide metabolites in Treg cells, which leads to the inhibition of the Mammalian Target of Rapamycin complex 1/protein kinase B (mTORC1/AKT) pathway.^34^ Additionally, it is likely that PS can suppress inflammatory signaling pathways and inhibit the release of inflammatory cytokines.^20^ Therefore, coffee consumption can directly or indirectly inhibit inflammation.

Another interesting aspect of coffee that has attracted attention is its antioxidant and anti‐immunosenescent effects. The anti‐immunosenescence potential of coffee can be attributed to its antioxidant ability to suppress the production of metalloproteinases (MMPs).^35^ In our study, we also observed anti‐immunosenescent effects of coffee, including decreased mRNA expression levels of MMP‐9. CD57 is another key marker of immunosenescent cells.^36^ Our results revealed a reduced frequency of many CD57^+^ T‐cell subsets after coffee consumption, which were negatively correlated with the abundances of SM, Cer‐NS, and galactosylceramide (Figure S6).

Our results showed significantly downregulated levels of proinflammatory genes and high expression of anti‐inflammatory genes after coffee consumption. Previous research has indicated that coffee consumption can suppress inflammation, as indicated by decreased mRNA expression levels of *STAT1*, *TNF*, *IFNG*, and *PPARG*.^37^ Our findings also suggested that coffee intake led to a significant reduction in the mRNA expression levels of immunosenescence marker genes such as *p16*, *p53*, *p21*, and *CD57*, as well as proinflammatory genes such as *IL17A* and *IFNγ*. The results showed that the mRNA expression of the anti‐inflammatory gene *IL10* increased. Furthermore, consistent with previous studies, our analysis revealed a significant increase in the mRNA expression level of *IL6* after coffee intake.^38^ These results indicate that coffee consumption has potential anti‐immunosenescence and anti‐inflammatory effects.

Furthermore, we utilized in vitro experiments to confirm the capacity of coffee metabolites in reducing immunosenescence, showing that 1‐MX, PX, and Cer administration reduced the frequency of CD4^+^CD57^+^ T cells, while bilirubin administration increased their frequency. We also validated the effects of coffee in keyhole limpet haemocyanin (KLH)‐immunized mice and IMQ‐induced psoriasis‐like mice. Inflammation‐related factors were reduced in the coffee intervention group. The coffee intervention group showed a lower frequency of CD4^+^ and CD8^+^ effector T cells and a greater frequency of Treg cells in IMQ‐induced psoriasis‐like mice. Additionally, there was a lower frequency of IL4^+^ and IFN‐γ^+^ immune cells in the skin. In addition, immunosenescence can promote tumor progression. By activating the *p38* pathway, tumor cells can activate immunosenescence, and then promote the expression of *p53*, *p21*, and *p16*.^36^, ^39^, ^40^ A previous study revealed a negative correlation between coffee consumption and the likelihood of developing liver and endometrial cancer. It is probable that coffee consumption could be linked to a lower likelihood of disease progression and mortality in individuals with advanced or metastatic colorectal cancer.^41^ Our results showed that the mRNA expression levels of *p16* and *p21* were reduced after coffee intake, suggesting that coffee consumption might also have an anti‐cancer capacity.

This current study has several limitations. First, we used a single coffee subtype (sugar‐free ground black coffee) for the coffee intervention. A significant important study involving a large group of participants revealed that consuming moderate amounts of both sugar‐free and sugary coffee was linked to a decreased likelihood of mortality.^42^ Thus, further study of different coffee subtypes, specifically distinguishing between forms and preparations of coffee,^43^ is also important for measuring the health effects of coffee. Similarly, the variation in the ability of coffee to reduce inflammation may be attributed to the complex nature of coffee, which contains more than 1000 chemical components. However, the subtypes of coffee consumed by habitual coffee consumers are variable, so whether this was the reason for the inconsistency with our postintervention results requires further analysis.

In summary, our study represents a “clinical” investigation elucidating the characteristics of the coffee metabolome and its correlation with immune alterations. Our findings provide evidence for the anti‐inflammatory and anti‐immunosenescence effects associated with coffee consumption and its byproducts, suggesting that coffee is a beneficial dietary behavior for human health.

## 4 MATERIALS AND METHODS

### 4.1 Human subjects

After excluding those who did not meet the criteria, we recruited a total of 86 volunteers, including 36, 24, and 26 subjects in the coffee nondrinking group, habitual coffee drinking group and coffee intervention group, respectively. Individuals who consumed more than one cup of coffee per day for a duration exceeding 6 months and for a minimum of 20 days per month were classified as habitual coffee drinkers. Individuals who refrained from consuming coffee or any caffeinated beverages for a period exceeding 3 months were classified as coffee nondrinkers. The coffee intervention group was recruited from the coffee nondrinking group.

The inclusion criteria were as follows: healthy volunteers, aged between 18 and 40 years, and had a body mass index (BMI) within the normal range of 18−24.

The exclusion criteria were as follows: (1) had no chronic disease; (2) had not taken antibiotics or other drugs in the previous 3 months; (3) had not received a vaccine via injection in the past 6 months; and (4) had no other cancers or inflammatory diseases. Volunteers who drank tea or smoked were also excluded because our primary outcome measure was to study the immune and metabolic changes associated with coffee intake (Figure 6). Tea consumption and smoking are widely recognized as lifestyle factors that have distinct effects on inflammation.^44^, ^45^

A total of 86 subjects were included in our study, including 36, 24, and 26 subjects in the coffee nondrinking group, habitual coffee drinking group, and ground coffee intervention group, respectively. Our results showed that there was no significant difference in BMI between the coffee nondrinkers and those who habitually consumed coffee (*p* = 0.091) and before and after the ground coffee intervention. However, in our study, compared with that of coffee nonconsumers (23.72 ± 0.3024), the age of habitual coffee consumers (25.79 ± 0.6084) was significantly greater (*p* ≤ 0.05 between the control group [36 coffee nonconsumers + 26 people before ground coffee intervention] and habitual coffee consumers [*n* = 24]), which may be associated with characteristics of the population that consumes coffee, such as their work or the economy (see Table 3). The same person extracted the coffee using the same espresso machine and ensured the same amount of coffee per cup. Participants drank coffee between 9 a.m. and 12 p.m. (1 cup/day, ∼1 ounce/cup). Participants were told not to consume any other food/drinks containing caffeine (including tea, soda, or other dietary sources) during the period.

**TABLE 3:** Descriptive statistics and characteristics of the coffee nonconsumers, habitual coffee consumers, and ground coffee intervention groups.

	Coffee nonconsumers (*n* = 36)	Habitual coffee consumers (*n* = 24)	Ground coffee intervention group (*n* = 26)	
			Before	After
Age (years)^[Link]^, ^[Link]^	23.72 ± 0.3024	25.79 ± 0.6084	24.44 ± 0.6413	
Sex				
Male, % (*n*)	6	5	9	
Female, % (*n*)	30	19	17	
Smoking before blood sample				
Yes, % (*n*)	0	1	1	
No, % (*n*)	30	23	25	
Tea consumption before blood sample				
Yes but <4 cups/month, % (*n*)	2	7	2	
No, % (*n*)	34	17	24	
Coffee before blood sample				
No, % (*n*)	36	–	26	–
≥1 to ≤3 cups/day	–	23	–	26
>3 cups/day	–	1	–	–
Antibiotics (within previous 3 months)	No	No	No	
Chronic disease, cancer, inflammatory diseases, etc.	No	No	No	

*Note*: Values are presented as the means ± standard errors of the mean (SEMs).

^a^ *p* ≤ 0.05 between control (36 coffee nonconsumers + the 26 people before the ground coffee intervention) and habitual coffee consumers (*n* = 24).

^*^ *p* ≤ 0.05.

### 4.2 Sample collection

After taking 20 mL of blood from volunteers on the first day, peripheral blood mononuclear cells (PBMCs) were isolated and used to analyze subsets of immune cells by flow cytometry and the mRNA expression of several genes by RT‐qPCR and RNA‐seq. Fresh stool samples were collected from the volunteers, flash‐frozen in liquid nitrogen within 1 h and then stored in a −80°C freezer. In the coffee intervention group, blood and stool samples were collected again after 30 days of coffee consumption.

### 4.3 Metabolomic analysis and metabolite annotation

To ensure stable and accurate metabolome results, data quality control was conducted using Pearson correlation analysis (Figures S15–S17). The relationships between serum samples tested with ESI^−^ and ESI^+^ in individuals who regularly consumed coffee, between serum ESI^−^ and ESI^+^ samples, and between stool ESI^−^ and ESI^+^ samples in those who recently started coffee consumption were strongly consistent, suggesting that the reliability and stability of the data were high. Furthermore, the quality control samples showed close clustering in the principal component analysis of the serum ESI^−^ and ESI^+^ as well as in the stool samples, providing additional confirmation of the reliability of the data. Among the samples collected, 4246 metabolites with the Human Metabolome Database (HMDB); IDs were found, with 862 (ESI^−^) and 800 (ESI^+^) metabolites in the serum samples and 1209 (ESI^−^) and 1375 (ESI^+^) metabolites in the stool samples.

### 4.4 Flow cytometry analysis

PBMCs were obtained from individuals to examine alterations in T‐cell and B‐cell populations. Flow cytometry using a DxP AthenaTM (Cytek) was used to sort all samples, which were then analyzed using FlowJo_V10 software.

Various antibodies targeting different human cell surface markers, including those against CD19, CD27, CD38, IgG, CD56, CD16, CD11c, CD3, CD4, CD8, CXCR5, PD‐1, CXCR3, CCR4, CCR6, CD25, CD127, CD57, and CD45RA, were used in the experiment.

### 4.5 Statistical analysis

Statistical analyses were conducted using SPSS version 18.0. The data are presented as the means ± standard errors of the mean and were assessed for a normal distribution and comparable variance across groups.

All detailed methods can be found in the Supporting Information.

## AUTHOR CONTRIBUTIONS

Haijing Wu and Qianjin Lu designed the experiments. Pinglang Ruan wrote the manuscript. Pinglang Ruan and Ming Yang performed almost all experiments on human samples and data analysis. Xinyi Lv, Yiran Chen, and Kai Shen performed the PBMC experiments. Weijun Peng, Jianhua Huang, Di Zhao, and Hongli Li performed the metabolomics analysis. Weijun Peng, Haijing Wu, Yang Xiao, and Qianjin Lu edited the manuscript. Weijun Peng, Haijing Wu, and Qianjin Lu conducted the study, oversaw the research, and edited the manuscript. All authors have read and approved the final manuscript.

## CONFLICT OF INTEREST STATEMENT

The authors declare they have no conflicts of interest.

## ETHICS STATEMENT

The Ethics Committee of the Second Xiangya Hospital of Central South University (LYG2023009) reviewed and approved this study. Written informed consent was obtained from all participants. Approval number of clinical trial registration for the experiments at chictr.org.cn: ChiCTR2300069646. The animal experiments were carried out with the approval of the Animal Ethics Committee of the Second Xiangya Hospital of Central South University (20240519).

## Supporting information

Supporting Information

## Data Availability

All data supporting this paper are present within the paper and/or the Supporting Information. The original datasets are available: RNA‐seq data were upload to CNCB‐NGDC (HRA007393) and the metabolomics data were upload to EBI‐MetaboLights (www.ebi.ac.uk/metabolights/MTBLS10148).
