# Supplementary material for: Metabolic shifts during coffee consumption refresh the immune response: insight from comprehensive multiomics analysis
Source: MedComm (2020). 2024 Jun 17;5(7):e617. doi: 10.1002/mco2.617 (PMC11181901; doi:10.1002/mco2.617)
Supplement: Supplementary file 1 — Supporting Information [file MCO2-5-e617-s001.pdf]

Supplementary Materials

Metabolic shifts during coffee consumption refresh immune response: Insight from comprehensive multi-omics analysis

Table of contents

Supplemental methods 2

Supplemental Figure

Figure S1 9

Figure S2 10

Figure S3 11

Figure S4 12

Figure S5 12

Figure S6 13

Figure S7 13

Figure S8 14

Figure S9A 14

Figure S9B 15

Figure S10 16

Figure S11 17

Figure S12A 18

Figure S12B 19

Figure S12C 20

Figure S12D 21

Figure S12F 22

Figure S12G 22

Figure S13 23

Figure S14 23

Figure S15 24

Figure S16 25

Figure S17 26

Supplementary Tables 27

Table of contents

Supplemental methods

Flow cytometry analysis

Peripheral blood mononuclear cells (PBMCs) from Human were separated by Ficoll-Paque Plus (GE healthcare, cat. 17-1440-03) to get a single-cell suspension. About 2×10^6 cells were suspended in PBS and incubated with human Fc-R block (BD Pharmingen, 564765) at room temperature for 10 minutes, and then incubated with antibodies against surface markers at 4°C for 45 minutes in the dark.

RNA isolation and RT-qPCR

Total RNA was extracted using TRIzol reagent (Invitrogen). RNA quality control and concentration were detected by NanoDrop spectrophotometer (ND-2000, Thermo). mRNA was reverse-transcribed by the PrimeScript RT reagent Kit with a gDNA Eraser (TaKaRa, cat. RR047A) according to the manufacturer's protocols. qPCR was carried out with TB Green Premix Ex Taq™ (Tli RNaseH Plus) (TaKaRa, cat. RR420A) by using thermocycler (Roche, LightCycler 96). The fold change of target gene expression was calculated as $2^{-\Delta\Delta Ct}$ ($\Delta\Delta Ct=\Delta Ct$ of experimental group – ΔCt of control group), which was normalized to the control group. mRNA primers for *p16 (CDKN2A), p21 (CDKN1A), p53 (TP53), CD57, BAX, BCL-W, KLRG1, IFNB, IL-17A, IRF4, NF-kB, IL-10, IL-4, IL-6, IL1B, FOXP3, JAK3, TGF-β, PPARα, mTOR and GAPDH* were purchased from Tsingke Biotechnology Co.. All gene primers are shown below.

Genes	Forward primer (5′–3′)	Reverse primer (5′–3′)
p16	CTCGTGCTGATGCTACTGAGGA	GGTCGGCGCAGTTGGGCTCC
P21	AGGTGGACCTGGAGACTCTCAG	TCCTCTTGGAGAAGATCAGCCG
P53	CCTCAGCATCTTATCCGAGTGG	TGGATGGTGGTACAGTCAGAGC
CD57	GAAAGCAGCCTCCTTCGAGAAC	CCTCATTCACCAGCACTGGCTT
IL-10	GGCACCCAGTCTGAGAACAGCT	ACTCTGCTGAAGGCATCTCG
IL-4	CCGTAACAGACATCTTTGCTGCC	GAGTGTCCTTCTCATGGTGGCT
IL-6	AGACAGCCACTCACCTCTTCAG	TTCTGCCAGTGCCTCTTTGCTG

IL-1β	CAGAAGTACCTGAGCTCGCC	AGATTCGTAGCTGGATGCCG
IL-17A	CGGACTGTGATGGTCAACCTGA	GCACTTTGCCTCCCAGATCACA
TNF-α	CACCACTTCGAAACCTGGGA	TGTAGGCCCCAGTGAGTTCT
NF-κB	GCAGCACTACTTCTTGACCACC	TCTGCTCCTGAGCATTGACGTC
BAX	TCAGGATGCGTCCACCAAGAAG	TGTGTCCACGGCGGCAATCATC
BCL-W	CAAGGAGATGGAACCACTGGTG	CCGTATAGAGCTGTGAACTCCG
KLRG1	CCTTTTGCTGGATTGGTCTGAGG	TTGATGGCACCGCATGTCTGCA
PPARα	TCGGCGAGGATAGTTCTGGAAG	GACCACAGGATAAGTCACCGAG
IFNγ	CTTGGATTCCTACAAAGAAGCAGC	TCCTCCTTCTGGAACTGCTGCA
IRF4	GAACGAGGAGAAGAGCATCTTCC	CGATGCCTTCTCGGAACTTTCC
FOXP3	CCTGGTTGTGAGAAGGTCTTCG	TGCTCCAGAGACTGCACCACTT
JAK3	AGTGACCCTCACTTCCTGCTGT	GGCTGAACCAAGGATGATGTGG
TGF-β	TACCTGAACCCGTGTTGCTCTC	GTTGCTGAGGTATCGCCAGGAA
mTOR	AGCATCGGATGCTTAGGAGTGG	CAGCCAGTCATCTTTGGAGACC
GAPDH	GTCAAGGCTGAGAACGGGAA	TCGCCCCACTTGATTTTGGA

RNA-seq and bioinformatics analysis

The Novogene Company conducted the RNA extraction, sample detection, enrichment, amplification, library preparation, and Illumina sequencing. R Studio was employed for the analysis of all RNA-seq data. Differential gene expression analysis was performed using the 'limma' R package. Gene Ontology enrichment analysis was carried out using the cluster Profiler package. The analyses were based on a significance threshold of p-value < 0.05 and fold-change values > 1.5-fold to identify differentially expressed genes.

Sample preparations for metabolome **profiling of human serum and stool**

For serum analysis, 100 μL of serum sample was transferred to an Eppendorf tube and mixed with 300 μL of extraction solution (methanol) containing the internal standard L-2-Chlorophenylalanine (2 pg/mL). After 30 seconds of vortexing, the

samples were sonicated for 10 minutes in an ice-water bath. Subsequently, the samples were incubated at -40°C for 1 hour and centrifuged at 12,000 rpm for 15 minutes at 4°C. Following centrifugation, 100 μL of the supernatant was transferred to a fresh glass vial for LC-MS analysis.

For fecal sample analysis, 50 mg of fecal sample was weighed into an Eppendorf tube. Subsequently, 1000 μL of fecal extraction solution (acetonitrile:methanol:water = 2:2:1) containing the internal standard L-2-Chlorophenylalanine (2 μg/mL) was added. After 30 seconds of vortexing, the samples were homogenized at 40 Hz for 4 minutes and then sonicated for 5 minutes in an ice-water bath. This homogenization and sonication cycle was repeated three times. The samples were then incubated at -40°C for 1 hour and centrifuged at 10,000 rpm for 15 minutes at 4°C. Following centrifugation, 400 μL of supernatant was transferred to a fresh tube and dried in a vacuum concentrator at 37°C. The dried samples were reconstituted in 200 μL of 50% acetonitrile by sonication on ice for 10 minutes. The reconstituted samples were then centrifuged at 13,000 rpm for 15 minutes at 4°C, and 75 μL of fecal supernatant was transferred to a fresh glass vial for LC-MS analysis. To create an overall quality control sample, an equal aliquot of the supernatant from all samples was mixed.

Untargeted metabolomics by high-performance liquid chromatography–mass spectrometry

Metabolic extracts were obtained from serum and fecal samples via methanol protein precipitation, followed by subsequent processing and analysis. A Waters ACQUITY UPLC HSS T3 column, measuring 100 mm × 2.1 mm with a particle size of 1.8 micrometers, was maintained at 45 degrees Celsius. The mobile phase comprised A (water containing 0.1% formic acid) and B (methanol), flowing at a rate of 0.35 mL/min. Analysis was conducted using a Thermo Fisher UltiMate 3000 ultra-high-performance liquid chromatography system coupled with a Thermo Fisher Q-Exactive Orbitrap mass spectrometer. Both positive and negative ion scanning modes were employed for comprehensive spectrum signal acquisition. Proteo Wizard software was utilized to

convert the MS raw data file to mzXML format, followed by processing using XCMS (V.3.2). Processing included peak deconvolution, alignment, and integration, with Minfrac and cutoff values set at 0.5 and 0.3, respectively. Metabolite identification was facilitated using an internal MS2 database.

Metabolomics analysis

LC-MS/MS analyses were conducted utilizing an Agilent Technologies UHPLC System (1290, Santa Clara, CA), equipped with a UPLC BEH Amide column (2.1 × 100 mm, 1.7 μm, Waters). The elution gradient was set as follows: from 0 to 0.5 min, 95% B; from 0.5 to 7.0 min, linearly decreased from 95% to 65% B; from 7.0 to 8.0 min, reduced to 40% B; held at 40% B from 8.0 to 9.0 min; then quickly increased to 95% B from 9.0 to 9.1 min, and maintained at 95% B until 12.0 min. The column was maintained at 25°C, with the autosampler temperature set at 4°C. Injection volumes of 2 μL were used for both ESI^+ and ESI^- modes. MSI data acquisition in the 60-1200 Da range was performed using a 6550 QTOF mass spectrometer (Agilent Technologies). Additionally, MS/MS spectra acquisition during LC/MS experiments was facilitated using a Triple TOF 6600 mass spectrometer (AB Sciex) through information-dependent acquisition (IDA). The AnalystTF 1.7 software (AB Sciex) continuously evaluated full scan survey MS data to trigger MS/MS spectra collection based on predefined criteria. Each acquisition cycle targeted the 12 most intense precursor ions exceeding an intensity threshold of 100 units, with a collision energy of 30 eV and a cycle time of 0.56 seconds. ESI source parameters were set as follows: Gas 1 at 60 psi, Gas 2 at 30 psi, Curtain Gas at 35 psi, Source Temperature at 600°C, Declustering Potential at 60V, and Ion Spray Voltage Floating (ISVF) at 5000V for ESI+ mode and -4000V for ESI- mode.

Animal models

KLH immunization

C57BL/6J mice (8 weeks old) were subcutaneously immunized with an equal volume

(400 μl per mouse) of keyhole limpet hemocyanin (KLH) solution (0.5 mg/ml, Sigma), emulsified in complete Freund's adjuvant (CFA) (0.5 mg/ml, Sigma), administered on the tail. Subsequently, following immunization, mice were euthanized, and lymph nodes, spleen tissues, and serum samples were harvested. Flow cytometry analysis was conducted on the lymph nodes and spleen tissues. Enzyme-linked immunosorbent assay (ELISA) was utilized to quantify serum antigen-specific total IgG, IgG1, IgG2a, IgG2b, and IgM antibody levels.Total RNA from splenic cells and Spleen $CD4^+$ T cells were extracted to analyze the mRNA expression level of p16 and p21 by RT-qPCR.

Animals and IMQ-induced psoriasis mouse model

BALB/c mice, 8 weeks old, were purchased from Skobes biology Experimental Animal Technology Co.Ltd. (Henan, China) and kept under specific pathogen-free conditions. The mice would receive a daily topical dose of 62.5 mg IMQ cream (5%) (Aldara, 3 M Pharmaceuticals, MN) on their shaved back for six consecutive days. The mice were assigned to each group randomly, and the investigators were blinded to the group allocation during the experiment. Based on the scoring system called PASI, we scored erythema, scaling, and thickness on the scores from 0 to 4: none 0; slight 1; moderate 2; marked 3; very marked 4. After the mice were sacrificed, the lymph nodes, spleen tissues, and skin samples were collected for flow cytometry. On day 7， mice were euthanized, and the spleen tissues and skin lesion were harvested to analyze the frequency of immune cells by flow cytometry analysis. Total RNA from skin lesion, splenic cells and Spleen $CD4^+$ T cells were extracted to analyze the mRNA expression level of p16 and p21 by RT-qPCR.

$CD4^+$ T cells sorted from spleens of mice

Mouse spleen cells were isolated and ground to get lymphocytes. $CD4^+$ cells were sorted by magnetic beads according to the protocol (Miltenyi 130-117-043). Trizol lysed CD4 cells according to the protocol for subsequent RT-qPCR experiments.

The immune subtypes and their corresponding molecular markers on each panel

Human:

Th1	CD4^{+}CXCR3^{+}CCR6^{-}CCR4^{-}	Th2	CD4^{+}CXCR3^{-}CCR6^{-}CCR4^{+}
Th17	CD4^{+}CXCR3^{-}CCR6^{+}CCR4^{+}	Treg	CD4^{+}CD25^{hi}CD127^{lo}
Tfh	CD4^{+}CXCR5^{+}PD-1^{+}	TEMRA cells	CD8^{+}CD45RA^{+}
NK	CD56^{+}	DN B cells	CD19^{+}CD27^{-}IgD^{-}
Antibody secreting cells	CD19^{+}CD27^{hi}CD38^{hi}	switched memory B cells	CD19^{+}IgD^{-}CD27^{+}
DPB cells	CD19^{+}IgD^{+}CD27^{+}	naïve B cells	CD19^{+}IgD^{+}CD27^{-}

Mice:

Th1	CD4^{+}IFNg^{+}	CD4^{+}Naive T	CD4^{+}CD44^{-}CD62L^{+}
Th2	CD4^{+}IL4^{+}	CD4^{+}Effector T	CD4^{+}CD44^{-}CD62L^{-}
Th17	CD4^{+}IL17A^{+}	CD4^{+}T$_{CM}$	CD4^{+}CD44^{+}CD62L^{+}
Treg	CD4^{+}CD25^{+}FOXP3^{+}	CD4^{+}T$_{EM}$	CD4^{+}CD44^{+}CD62L^{-}
Tfh	CD4^{+}CXCR5^{+}PD-1^{+}	CD8^{+}Naive T	CD8^{+}CD44^{-}CD62L^{+}
GC B	B220^{+}FAS^{+}IgD^{-}	CD8^{+}Effector T	CD8^{+}CD44^{-}CD62L^{-}
PC	B220$^{+/-}$CD138^{+}	Memory B	B220^{+}IgD^{-}CD138^{-}
Naïve B	B220^{+}IgD^{+}CD138^{-}		

Anti-Human Antibodies

Marker	Fluorescein	Manufacturers	Cat. No.
CD19	BB515	BD	564456
CD27	APC-Cy7	Biolegend	356424
CD38	PerCP-Cy5.5	BD	551400
IgD	PE-CY7	Biolegend	348210
CD56	BV711	Biolegend	318336
CD16	PE	BD	555407
CD11c	APC	Biolegend	301614
CD3	FITC	Biolegend	317206
CD4	PE	BD	555347
CXCR5	APC-Cy7	Biolegend	356926
PD-1	APC	eBioscience	17-2799-42
CXCR3	BV421	Biolegend	356920
CCR4	BV605	Biolegend	359418
CCR6	Pe-dazzle594	Biolegend	353430
CD25	BV711	BD	563159
CD127	PerCP-Cy5.5	Biolegend	351322

CD57	PE-CY7	Biolegend	359624
CD45RA	BV785	Biolegend	304140
Zombie Aqua™	/	Biolegend	423102

Anti-Mouse Antibodies

Marker	Fluorescein	Manufacturers	Cat. No.
CD4	FITC	Biolegend	100406
IFN-γ	Percp-Cy5.5	BD	560660
IL-4	PE	BD	504104
IL-17A	APC	Biolegend	506916
CD25	PE	Biolegend	102008
FOXP3	APC	Invitrogen	17-57773-82
ZombieNIR	/	Biolegend	423106
CD8a	Percp-Cy5.5	Biolegend	100734
CD44	PECY7	Biolegend	103030
CD62L	PE	BD	553151
B220	FITC	BD	553088
IgD	Percp-Cy5.5	Biolegend	405710
CD138	BV421	BD	142508
FAS	PE	BD	554258
CD11c	AF647	Biolegend	117312
CD4	PE	Biolegend	100408
PD-1	APC	Biolegend	135210
Rat Anti-Mouse CXCR5	/	BD	551961
Anti-rat IgG2a	FITC	Invitrogen	PA1-84761

Data analysis

In metabolomics data analysis, we initially utilized Proteo Wizard to convert MS raw data files to mzXML format, followed by processing using XCMS (version 3.2) in the R environment. The processing steps involved peak deconvolution, alignment, and integration, with Minfrac and cutoff values set at 0.5 and 0.3, respectively. Subsequently, we employed an internal MS2 database for metabolite identification. To assess the stability of the dataset, we employed Pearson correlation coefficients based on relative quantification of QC samples as a standard metric. The KEGG and HMDB (https://hmdb.ca/metabolites) were used for metabolites annotation and pathway analysis. Correlation analysis of the metabolites with clinical parameters was performed using SPSS.

Supplemental Figure

Figure S1. The Gating strategy and representative flow cytometric plots.

(A): Gating strategy and representative flow cytometric plots of $CD3^+$, $CD4^+$ and $CD8^+$ T cells. Non_coffee, non-coffee consumption group; Coffee, habitual coffee drinking group.

(B): Gating strategy and representative flow cytometric plots of Th1, Th2 and Th17 cells.

(C): Gating strategy and representative flow cytometric plots of Treg cells and $CD4^+CXCR5^+PD1^+$ Tfh-like cells.

(D): Gating strategy and representative flow cytometric plots of $CD4^+CD45RA^+$ cells and $CD8^+CD45RA^+$ cells.

(E): Gating strategy and representative flow cytometric plots of $CD3^+CD57^+$ cells, $CD4^+CD57^+$ cells and $CD8^+CD57^+$ cells.

(F): Gating strategy and representative flow cytometric plots of $CD57^+$ T cells, respectively. Non_coffee, noncoffee consumption group; Coffee means habitual coffee drinking group.

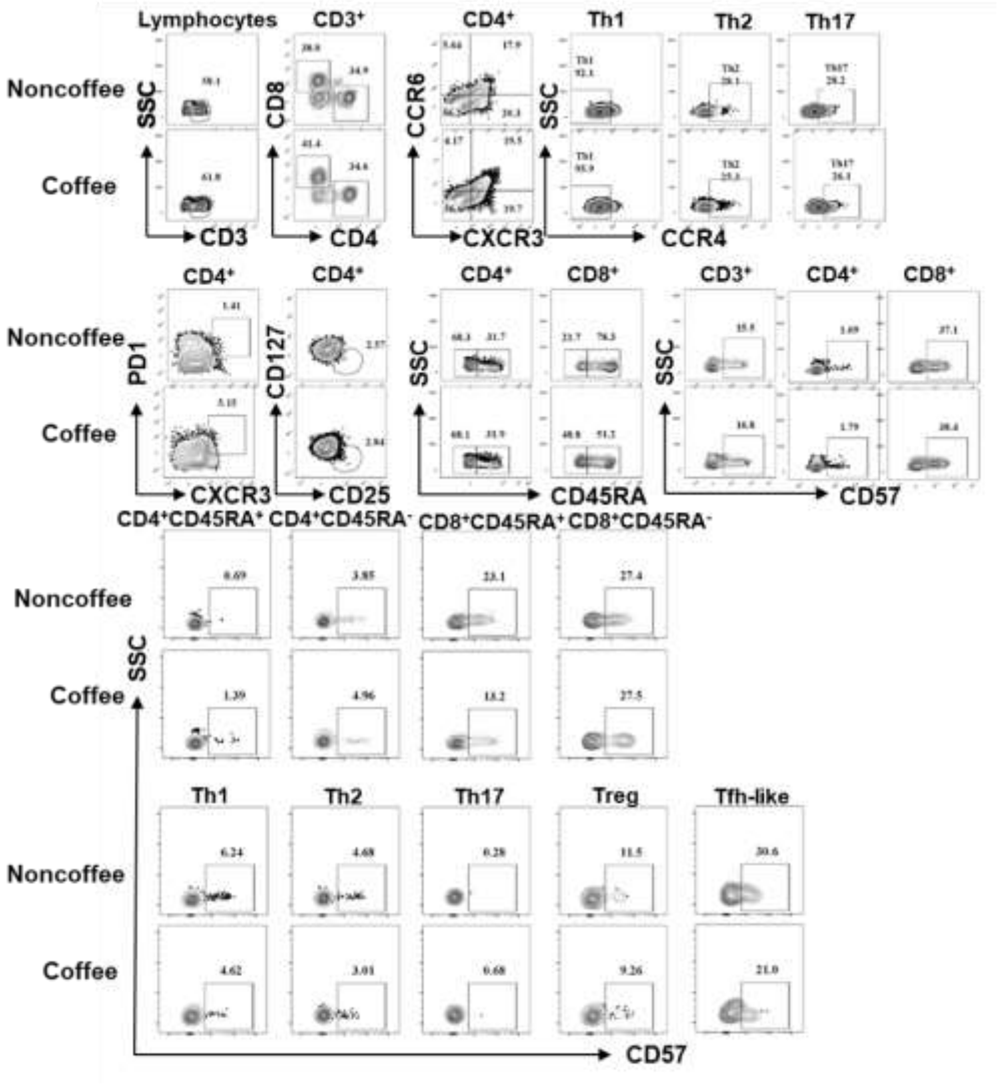

Figure S2. The effects of coffee consumption on T cells. Statistical analysis of the frequency of each T subset and $CD57^+$ T cells from PBMCs of non-coffee consumption consumers (n = 62) and habitual coffee consumers (n = 24). Bars represent the mean ± SEM.

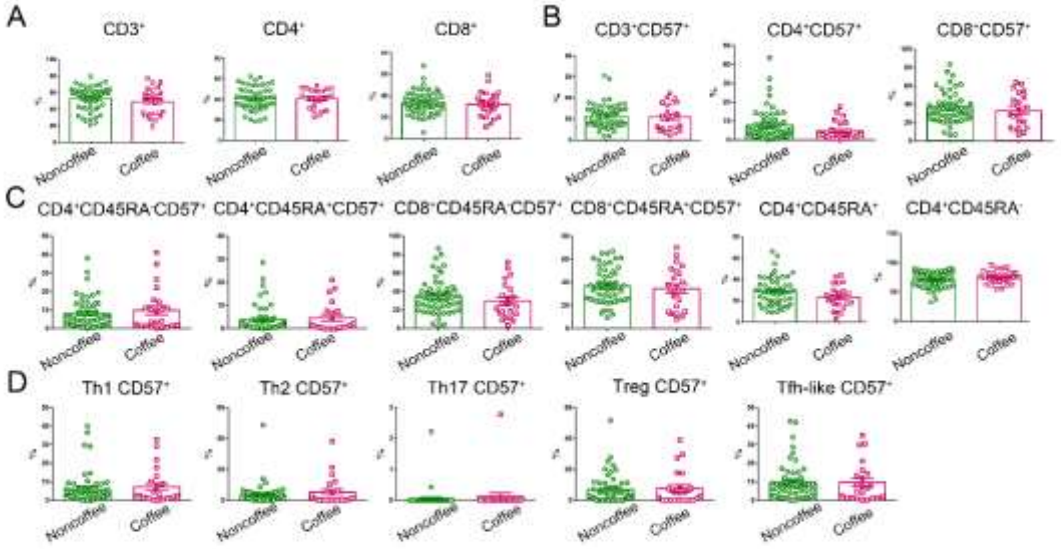

Figure S3. The effects of coffee consumption on B cells.

(A): Gating strategy and representative flow cytometric plots of B cells, respectively. Non_coffee, non-coffee consumption group; Coffee means habitual coffee drinking group.

(B): Statistical analysis for the frequency of each B cell subsets from PBMCs of noncoffee consumption consumers (n = 62) and habitual coffee consumers (n = 24). Noncoffee: non-coffee consumption group; Coffee means habitual coffee drinking group. Bars represent the mean ± SEM.

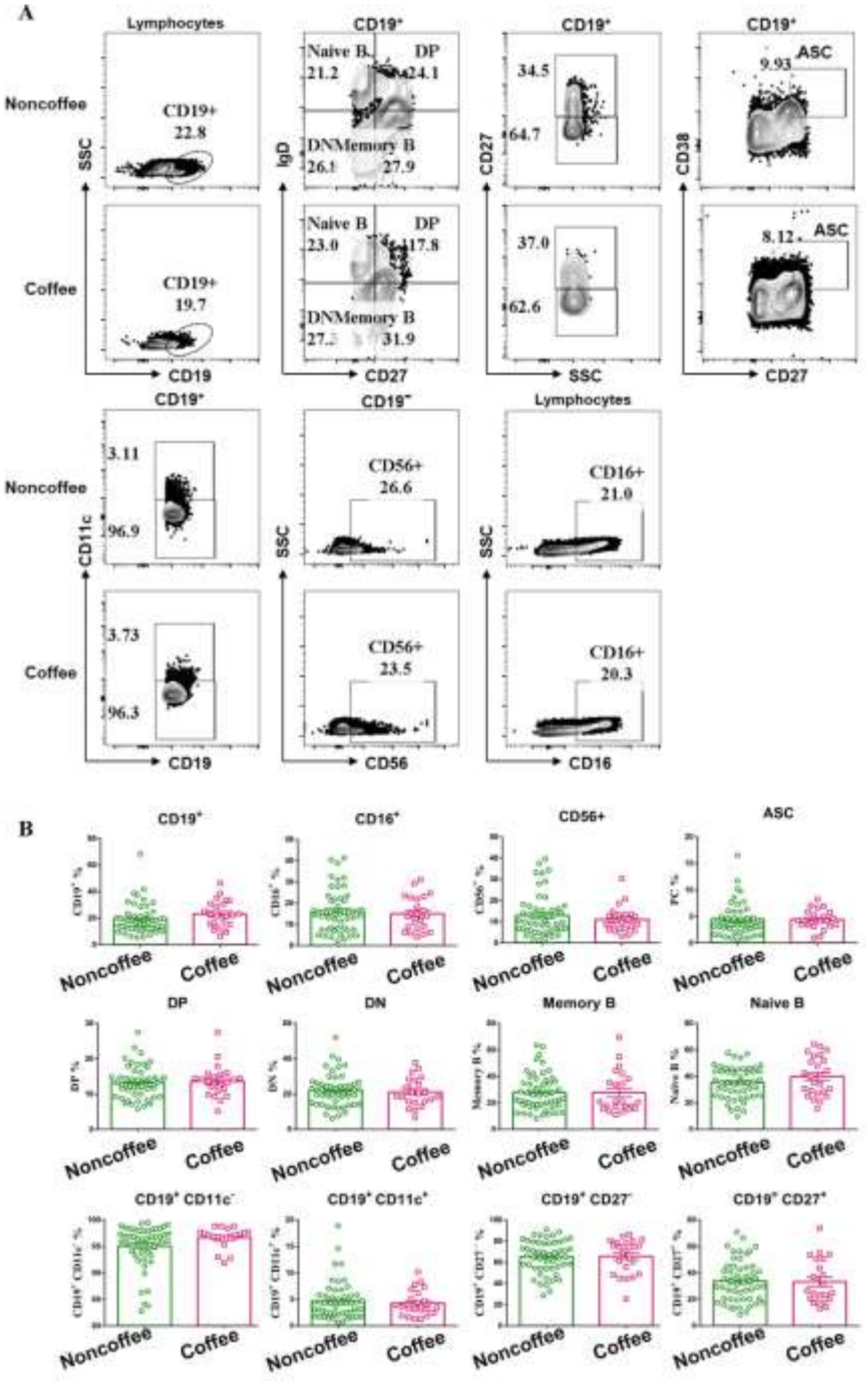

Figure S4. The significantly enriched coffee-associated metabolites between habitual coffee consumers and non-coffee consumption consumers based on their abundances. Non-coffee n = 62, Coffee n = 24, * $P < 0.05$, ** $P < 0.01$, *** $P < 0.001$, **** $P < 0.0001$.

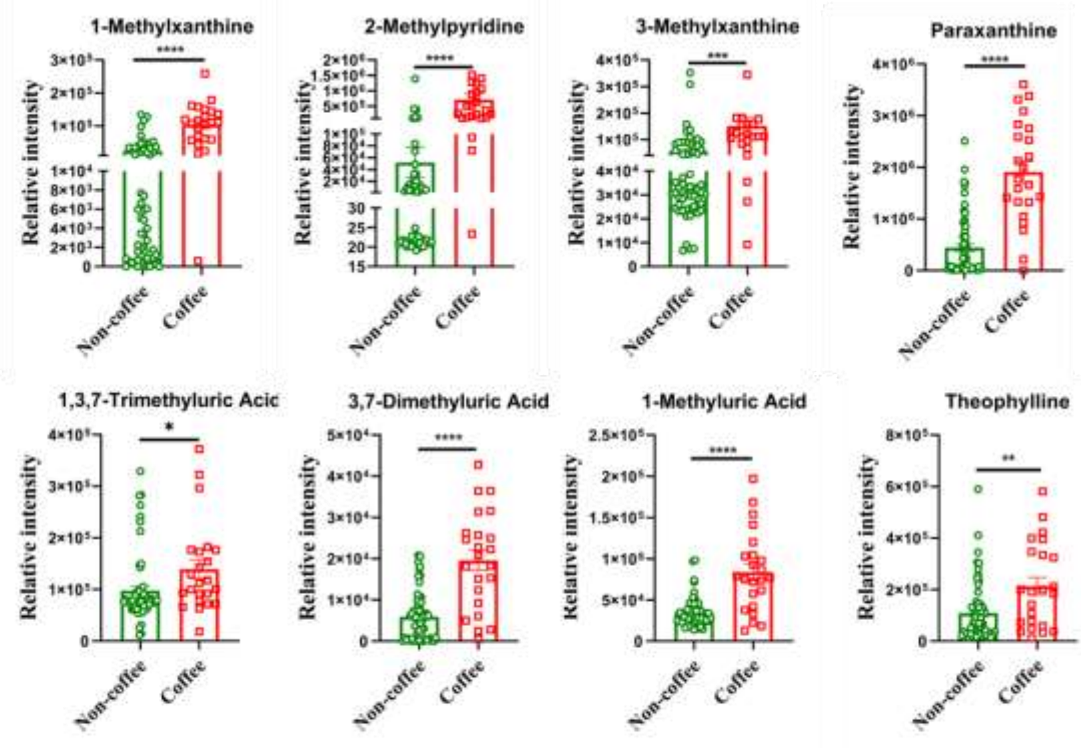

Figure S5. Statistical analysis of the frequency of each CD57$^+$ T-cell subset. Coffee_before n= 26, Coffee_after n = 26, * $P < 0.05$.

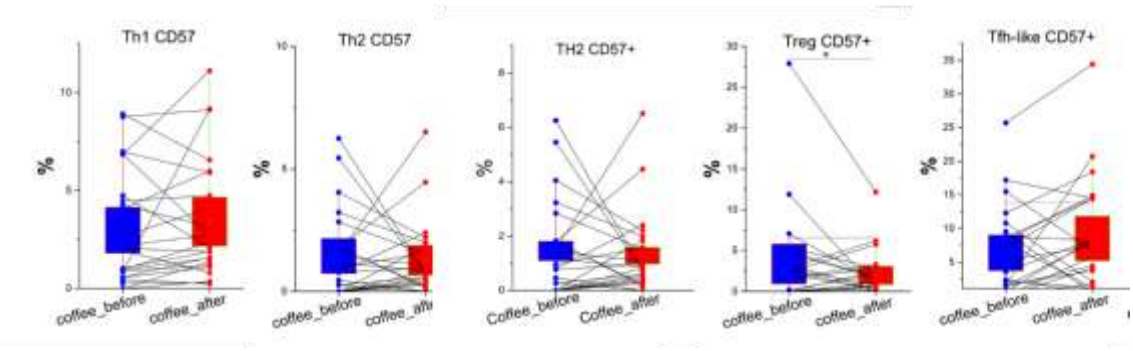

Figure S6. Statistical analysis of the frequency of each B-cell subset and other immune cells between before ground coffee consumption group and after ground coffee consumption group. C_before(Coffee_before) n = 26, C_after(Coffee_after) n = 26, * $P < 0.05$, *** $P < 0.001$.

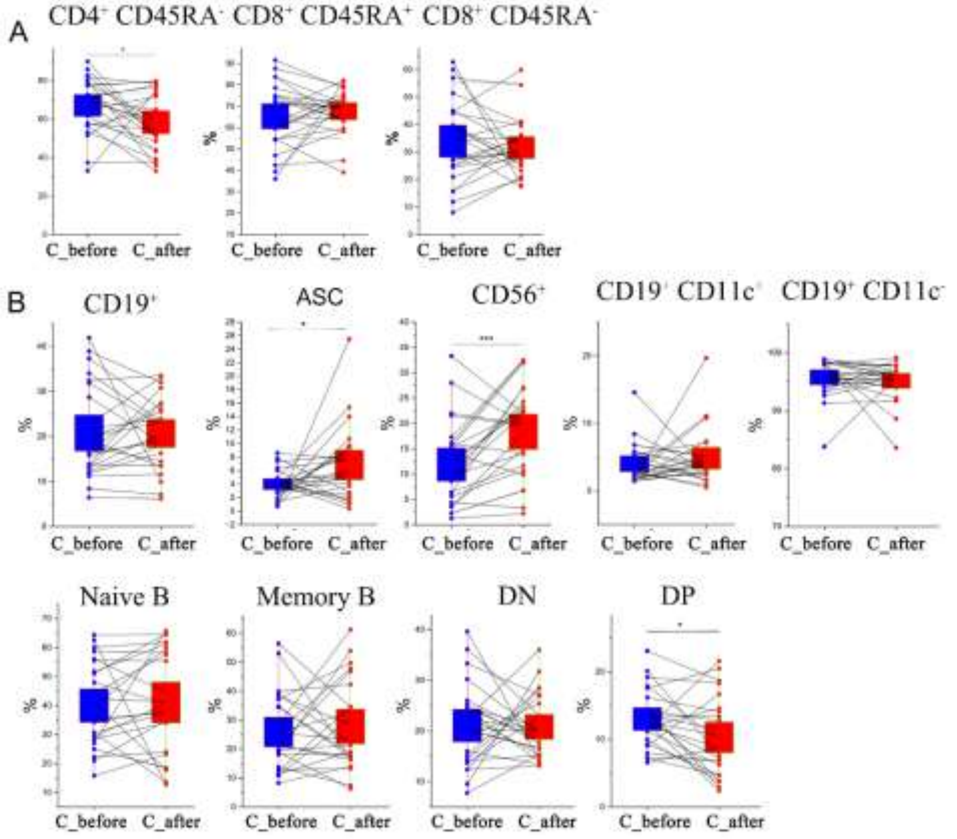

Figure S7. Transcriptomic changes in ground coffee consumption.

(A): Gene set enrichment analysis (GSEA) of RNAseq data.

(B): Heatmap showing the relative levels of mRNA expression clustered with complete clustering (n = 5 vs 5).

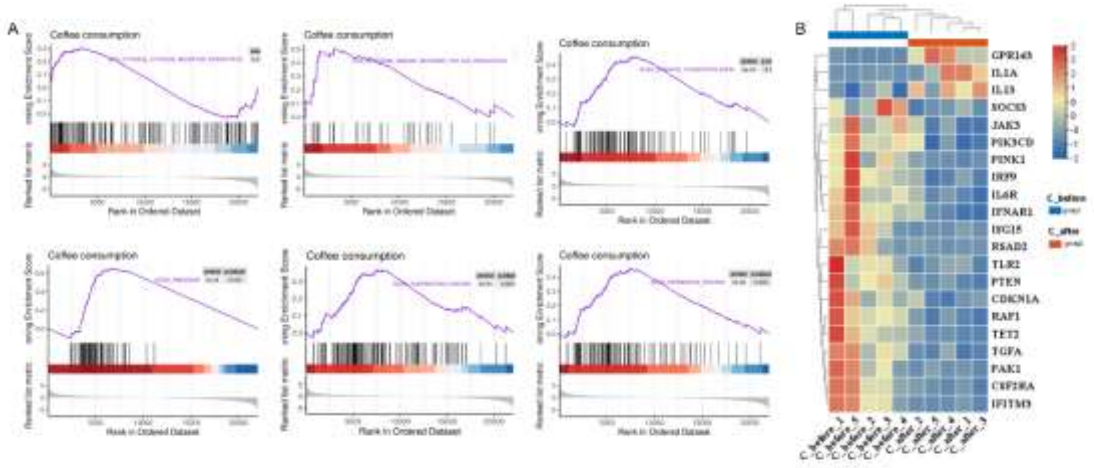

Figure S8. The relative expression of mRNA, including *p16, p21, p53, CD57, BAX, BCL-W, KLRG1, IFNB, IL17A, IRF4, NF-kB, JAK3, TGF-β* and *PPARα*. Green: non-coffee drinking group, n = 62; Red: the habitual coffee drinking group, n = 24. * $P < 0.05$. ** $P < 0.01$.

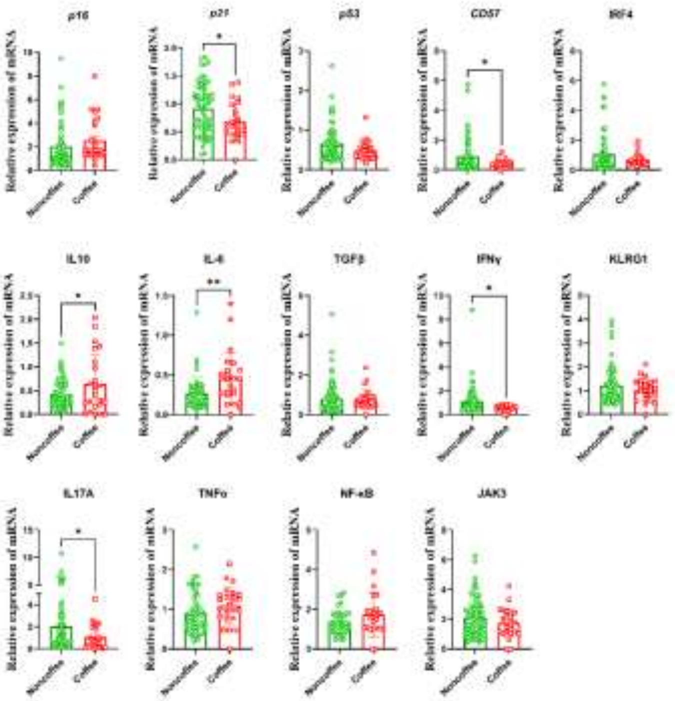

Figure S9A. The significantly enriched coffee-associated metabolites between before ground coffee consumption group and after ground coffee consumption group based on their abundances. Coffee-before n = 26, Coffee-after n = 26, * $P < 0.05$, ** $P < 0.01$, **** $P < 0.0001$.

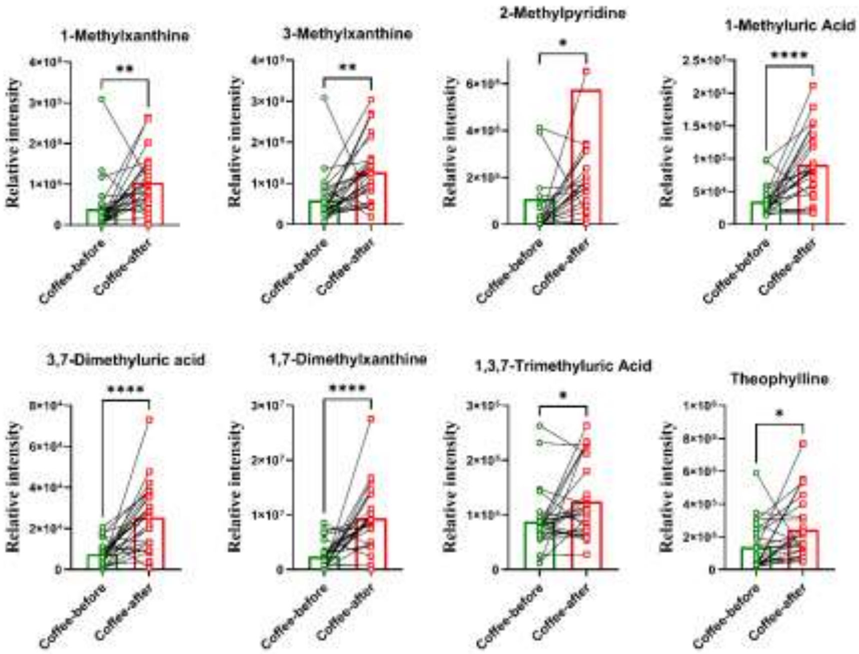

Figure S9B. The significantly enriched coffee-associated metabolites between before ground coffee consumption group and after ground coffee consumption group based on their abundances. Coffee-before n = 26, Coffee-after n = 26, * *P* < 0.05. ** *P* < 0.01, * *P* < 0.001, **** *P* < 0.0001.**

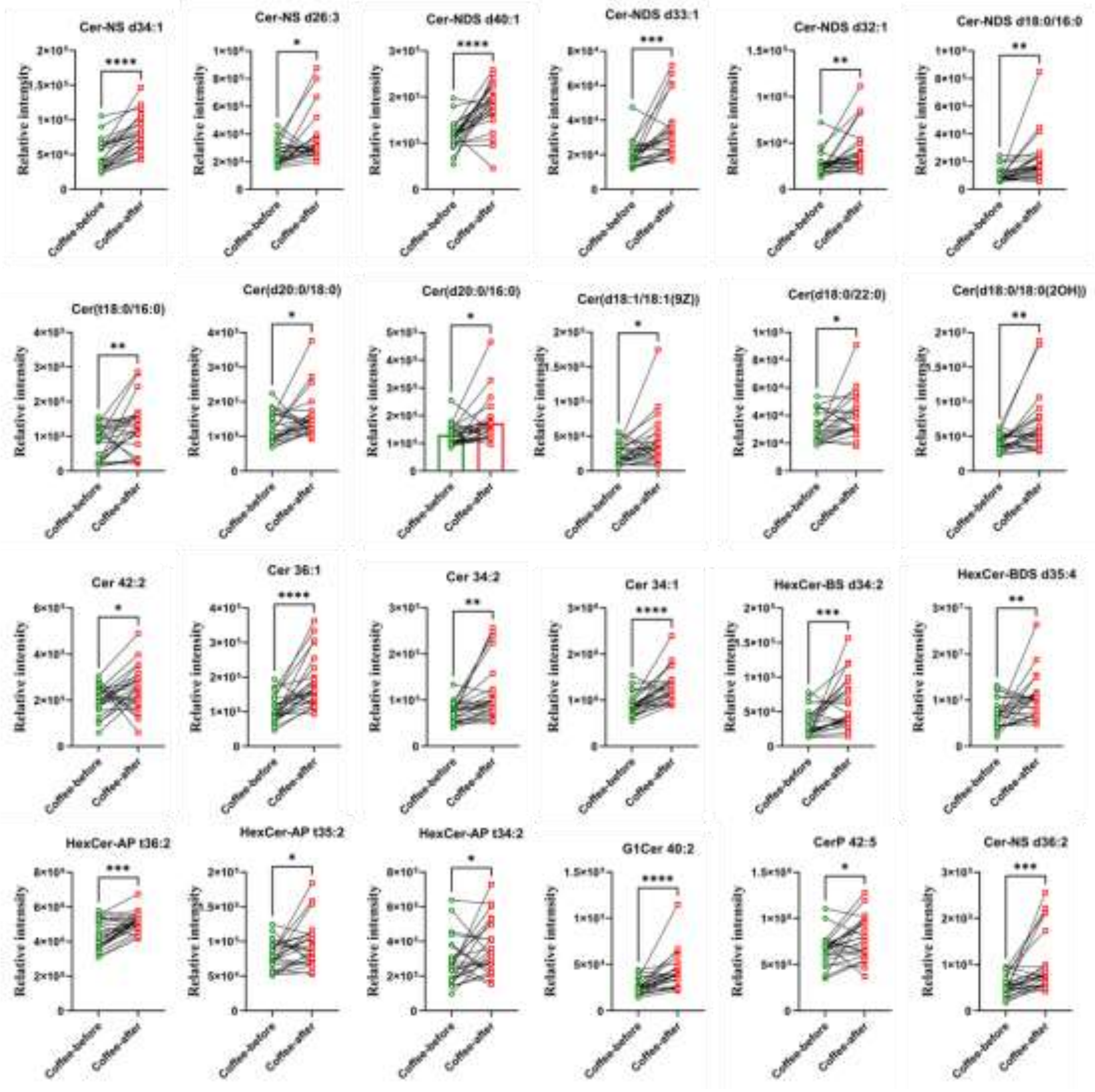

Figure S10. Metabolomic changes of stool samples in subjects before and after ground coffee consumption. (A)-(B): the metabolites analysis in stool samples from subjects before and after ground coffee consumption in ESI$^-$ (A) and ESI$^+$ (B) modes, respectively. (C): Fold change plots of identified metabolites. Log2-fold change value = 1, *P* < 0.05. (D): Heatmap showing the abundances of the metabolites in subjects before and after ground coffee consumption. Red colors indicate the abundances of the metabolites. (E): KEGG metabolic pathway analysis in subjects after ground coffee consumption. *P* < 0.05. Coffee-before n = 26, Coffee-after n = 26.

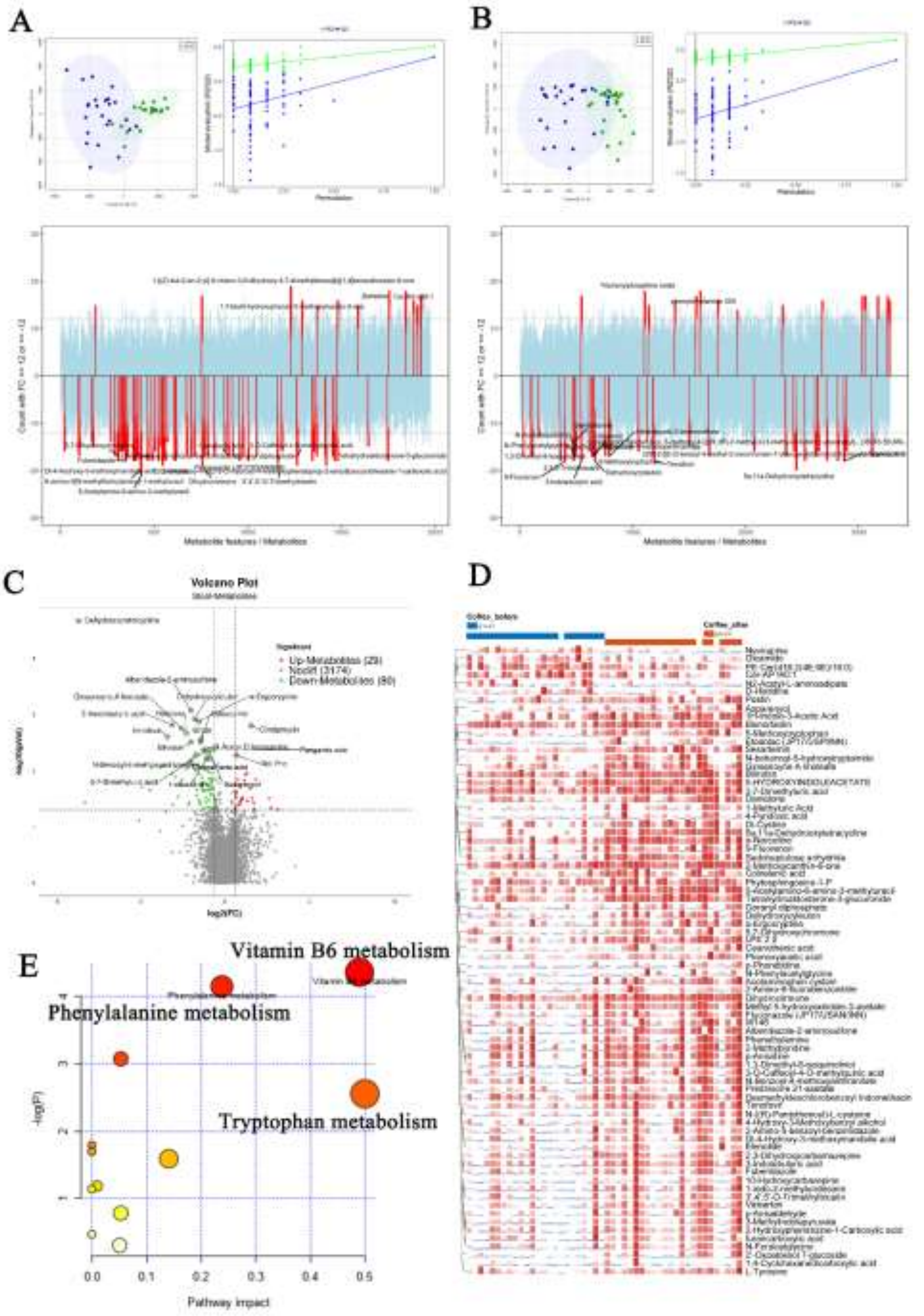

Figure S11. 31 significantly changed shared metabolites between the habitual coffee drinkers and ground coffee consumption group, including 15 in the ESI− (A) and 16 in ESI+ (B) models.

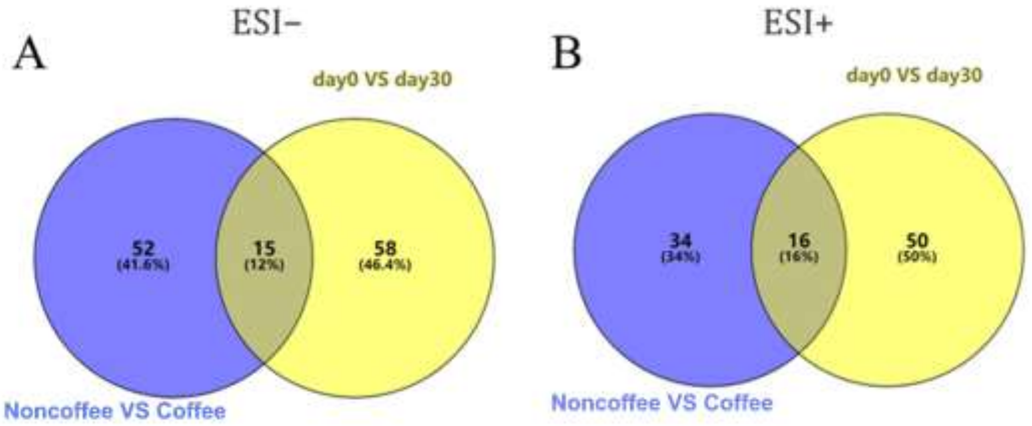

Figure 12A. Cells activity verification of adaptive immune regulation-related metabolites in coffee consumption *in vitro,* including bilirubin, 1-MX (1-methylxanthine), 1-MUA (1-methyluric Acid), 3-MX (3-methylxanthine), PX (paraxanthine) and Cer (ceramide mix metabolites). Ctrl n = 4, Metabolites (1-MX, 1-MUA, 3-MX, PX, Cer, Bilirubin) n = 4.

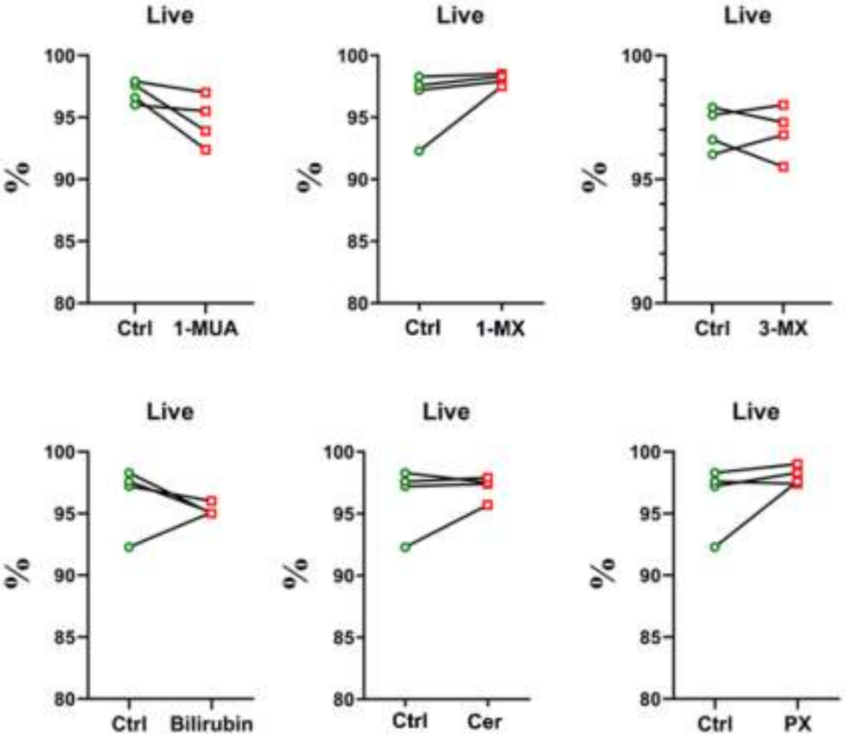

Figure 12B. The effects of 1-MX (1-methylxanthine) on T cells *in vitro.* Statistical analysis of the frequency of each CD4⁺ T-cell subset. Ctrl n = 4, 1-MX n = 4, ** *P* < 0.01.

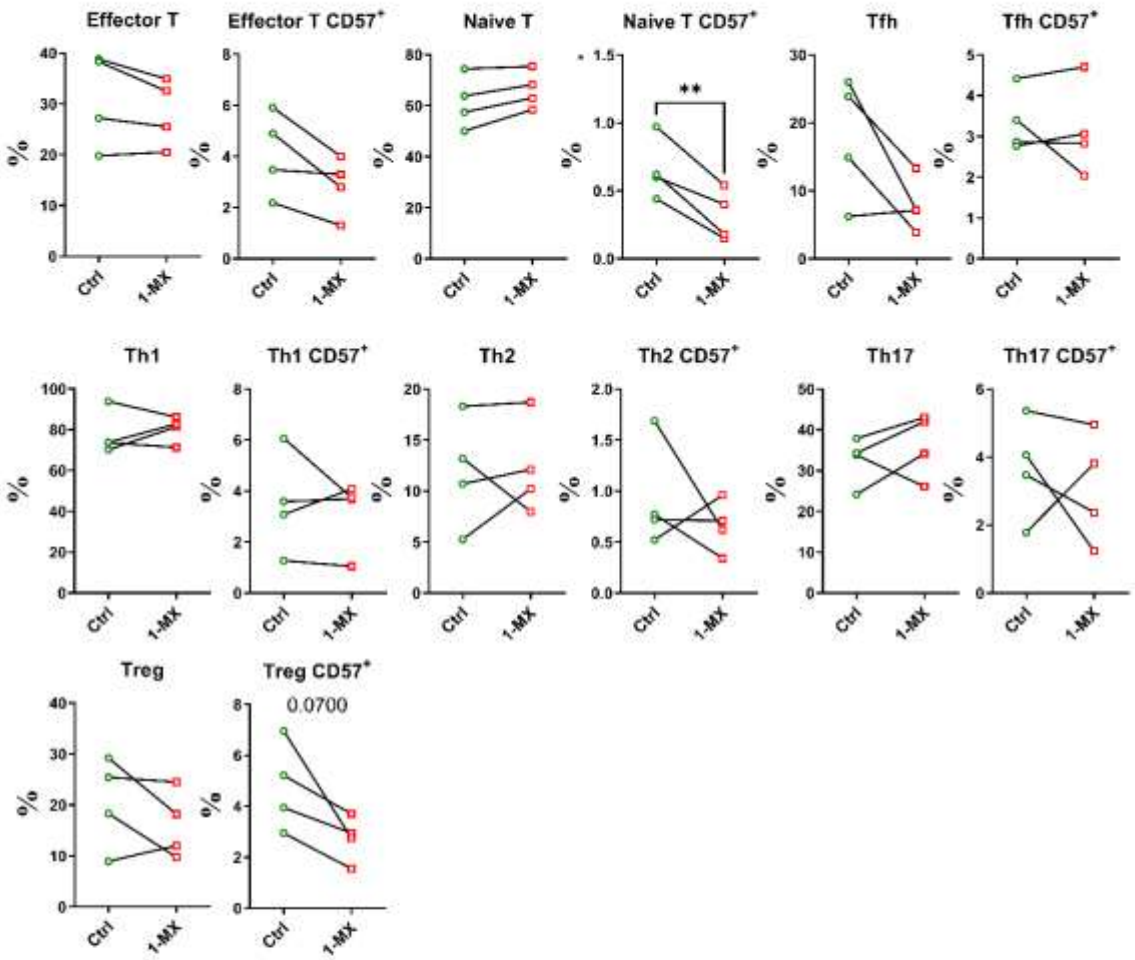

Figure 12C. The effects of 3-MX (3-methylxanthine) on T cells *in vitro.* Statistical analysis of the frequency of each CD4^{+} T-cell subset. Ctrl n = 4, 3-MX n = 4, * $P < 0.05$.

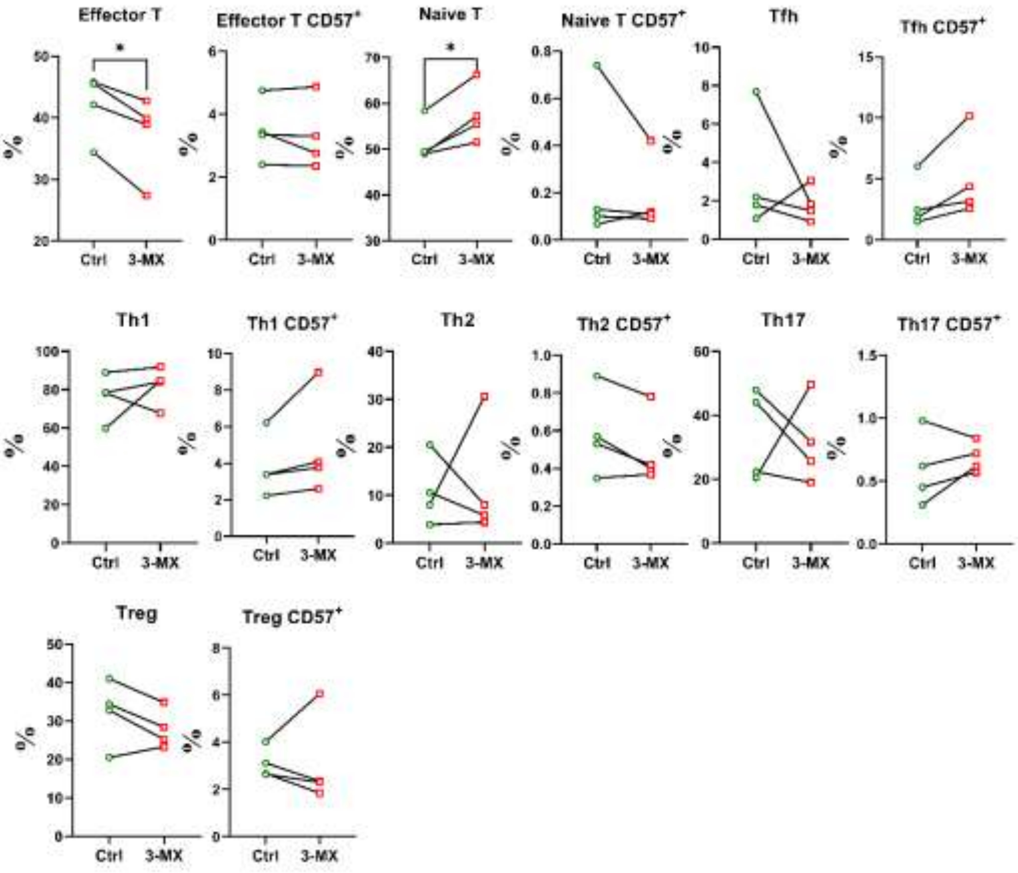

Figure 12D. The effects of 1-MUA (1-methyluric Acid) on T cells *in vitro.* Statistical analysis of the frequency of each CD4⁺ T-cell subset. Ctrl n = 4, 1-MUA n = 4, * *P* < 0.05.

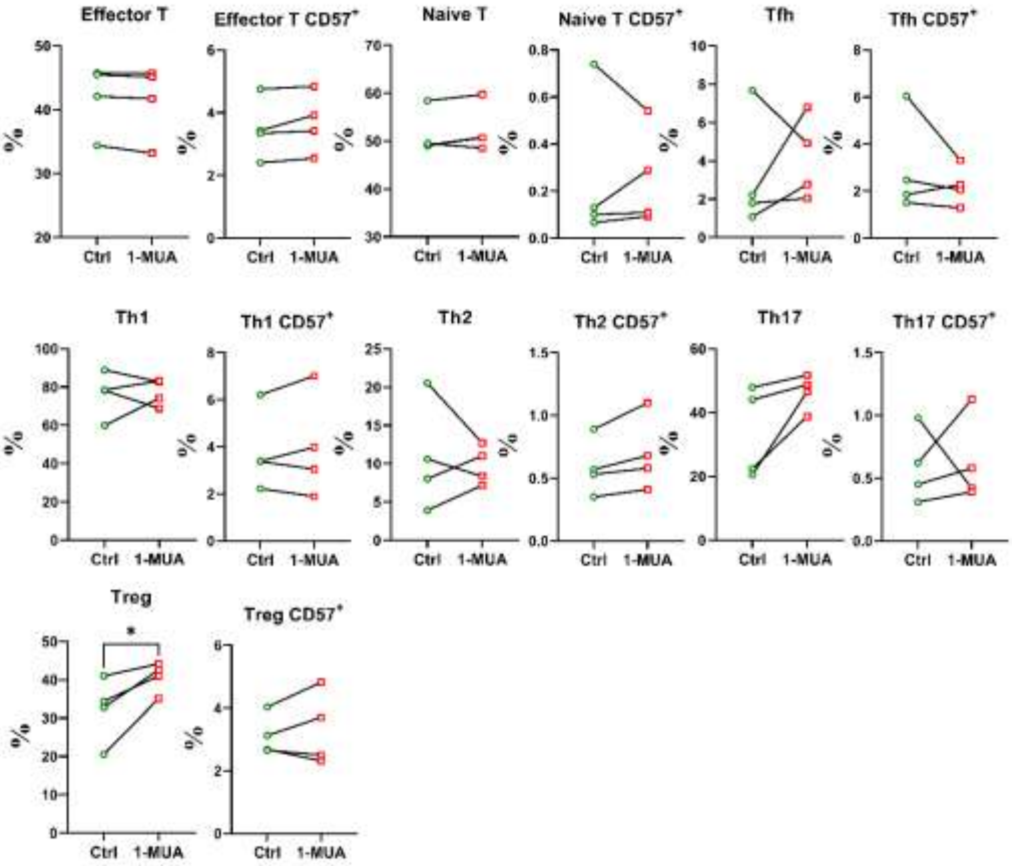

Figure 12E. The effects of PX (paraxanthine) on T cells *in vitro*. Statistical analysis of the frequency of each CD4^{+} T-cell subset. Ctrl n = 4, PX n = 4, * $P < 0.05$. ** $P < 0.01$.

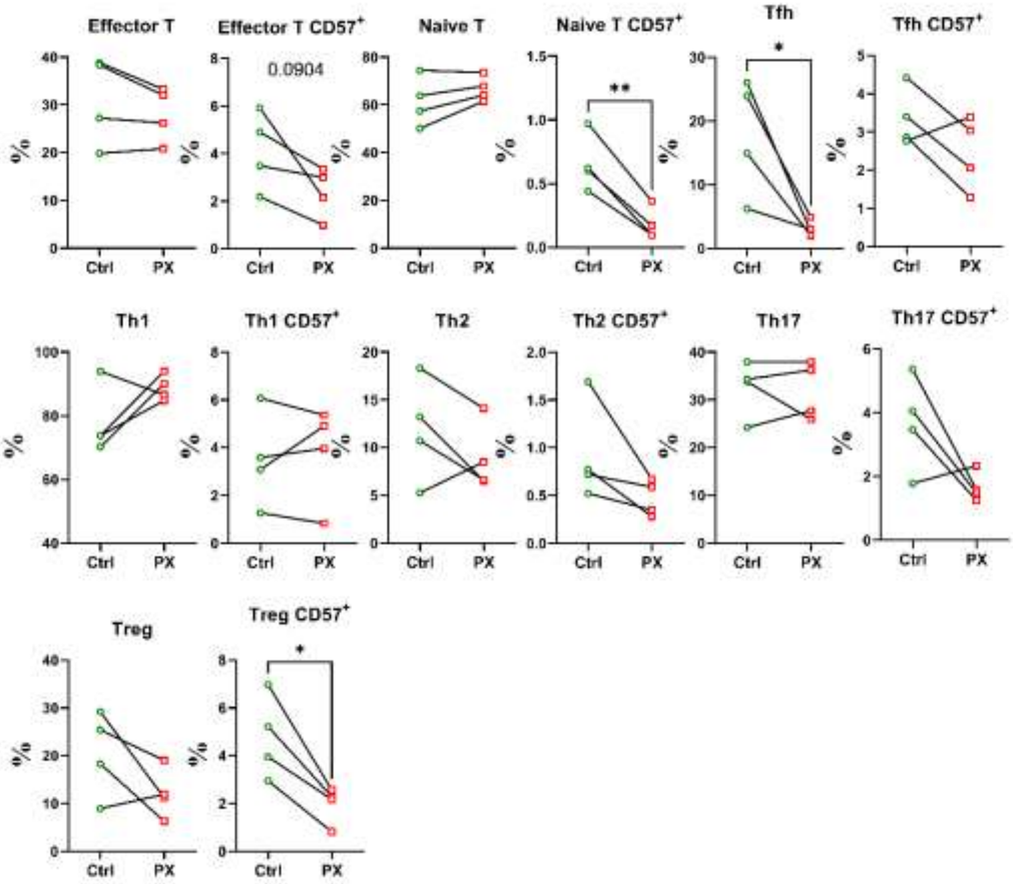

Figure 12F. The effects of Cer (ceramide mix metabolites) on T cells *in vitro.* Statistical analysis of the frequency of each CD4^{+} T-cell subset. Ctrl n = 4, Cer n = 4, * $P < 0.05$, *** $P < 0.001$.

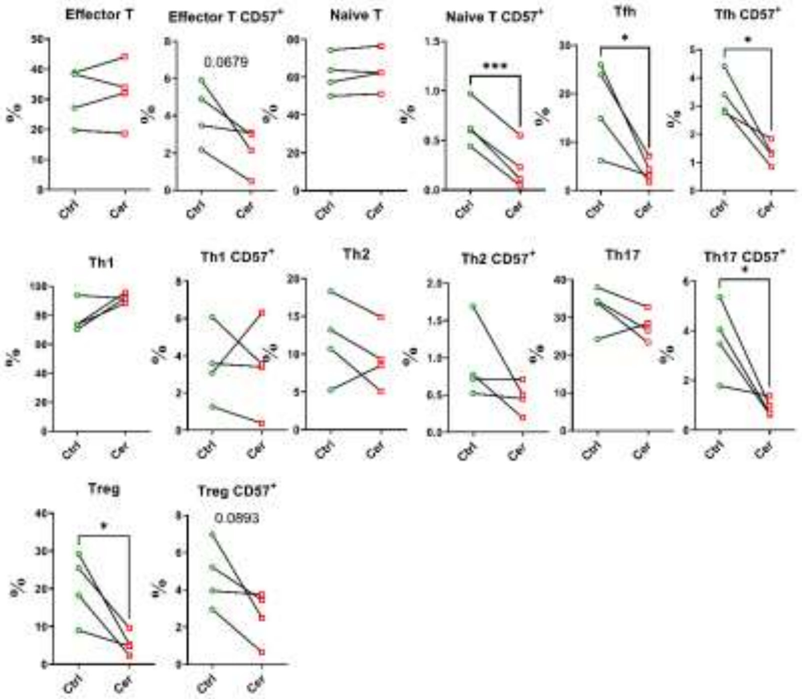

Figure S12G. The effects of bilirubin on T cells *in vitro.* Statistical analysis of the frequency of each CD4^{+} T-cell subset. Ctrl n = 4, Bilirubin n = 4, * $P < 0.05$.

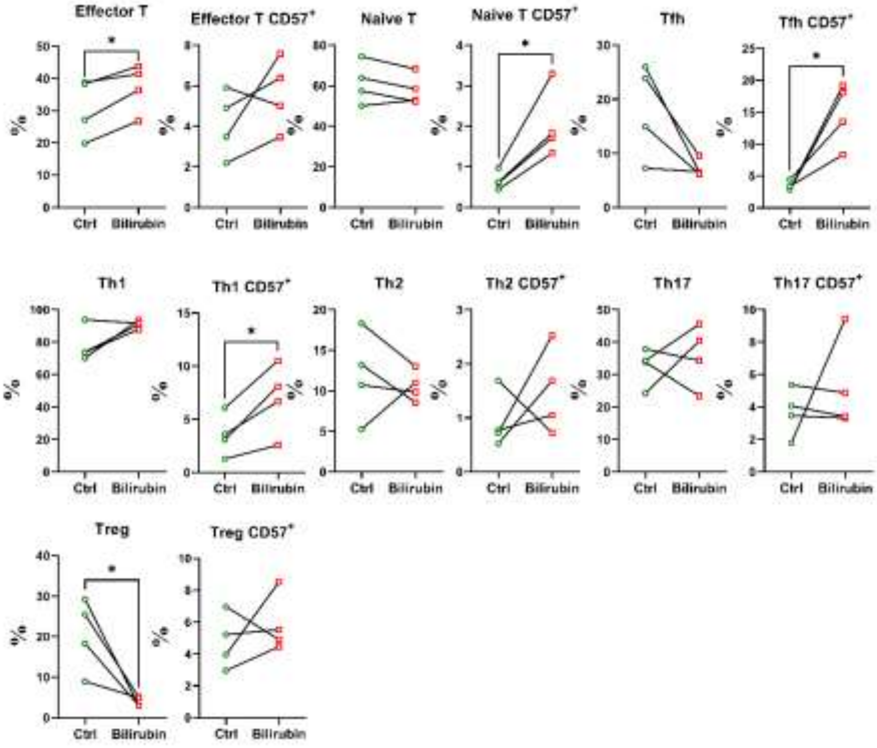

Figure S13. Coffee consumption alleviated imiquimod (IMQ) induced psoriasis-like inflammation of mice. (130 mg/kg/day).

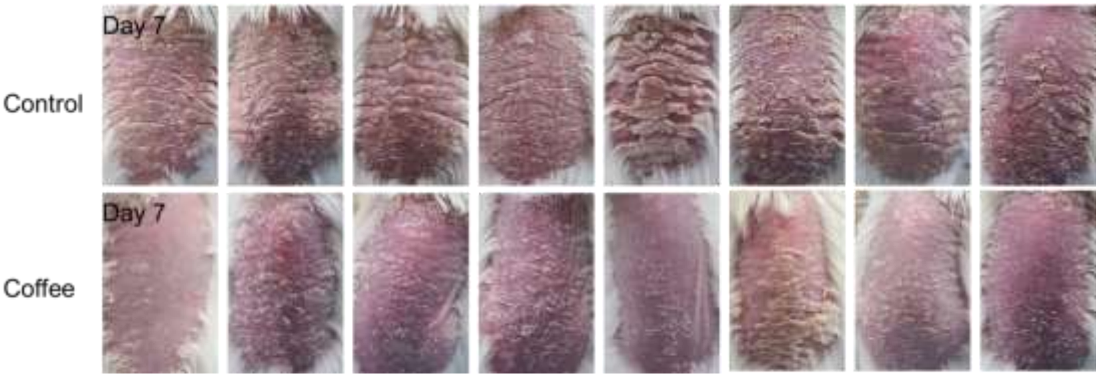

Figure S14. The effects of anti-inflammatory and anti-immunosenescence effects of coffee in KLH immunization-mice. A: Statistical analysis of the percentages of cells in spleens and LNs (lymph nodes). B: The serum levels (OD_{450nm} value) of total IgG, IgG1, IgG2a, IgG2b and IgM from the KLH-immunized mice on day 0, 7, 14, and 21. C: Statistical analysis of mRNA levels of *p16* and *p21* in spleens cells and $CD4^+$ T cells from spleens. Horizontal bars represent the mean ± SEM. Control n = 10, Coffee n = 10, * *P* < 0.05, ** *P* < 0.01, * *P* < 0.001, **** *P* < 0.0001.**

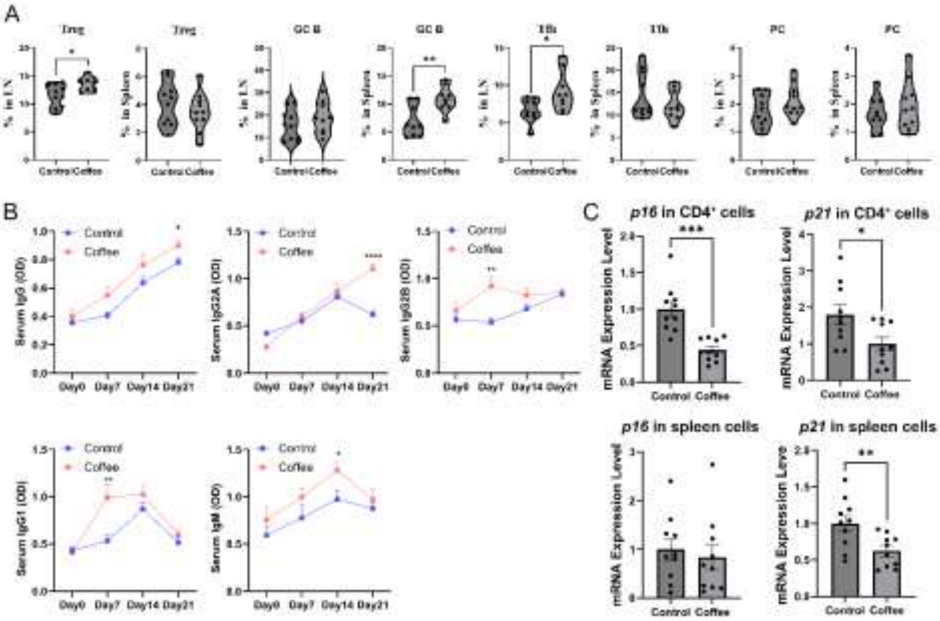

Figure S15. The correlation of serum ESI$^-$ (A) and ESI$^+$ (B) QC samples in the habitual coffee drinkers, serum ESI$^-$ (C) and ESI$^+$ (D) QC samples and stool ESI$^-$ (E) and ESI$^+$ (F) QC samples in the short-term coffee drinkers. ESI-, negative electrospray ionization; ESI$^+$, positive electrospray ionization. QC, quality control.

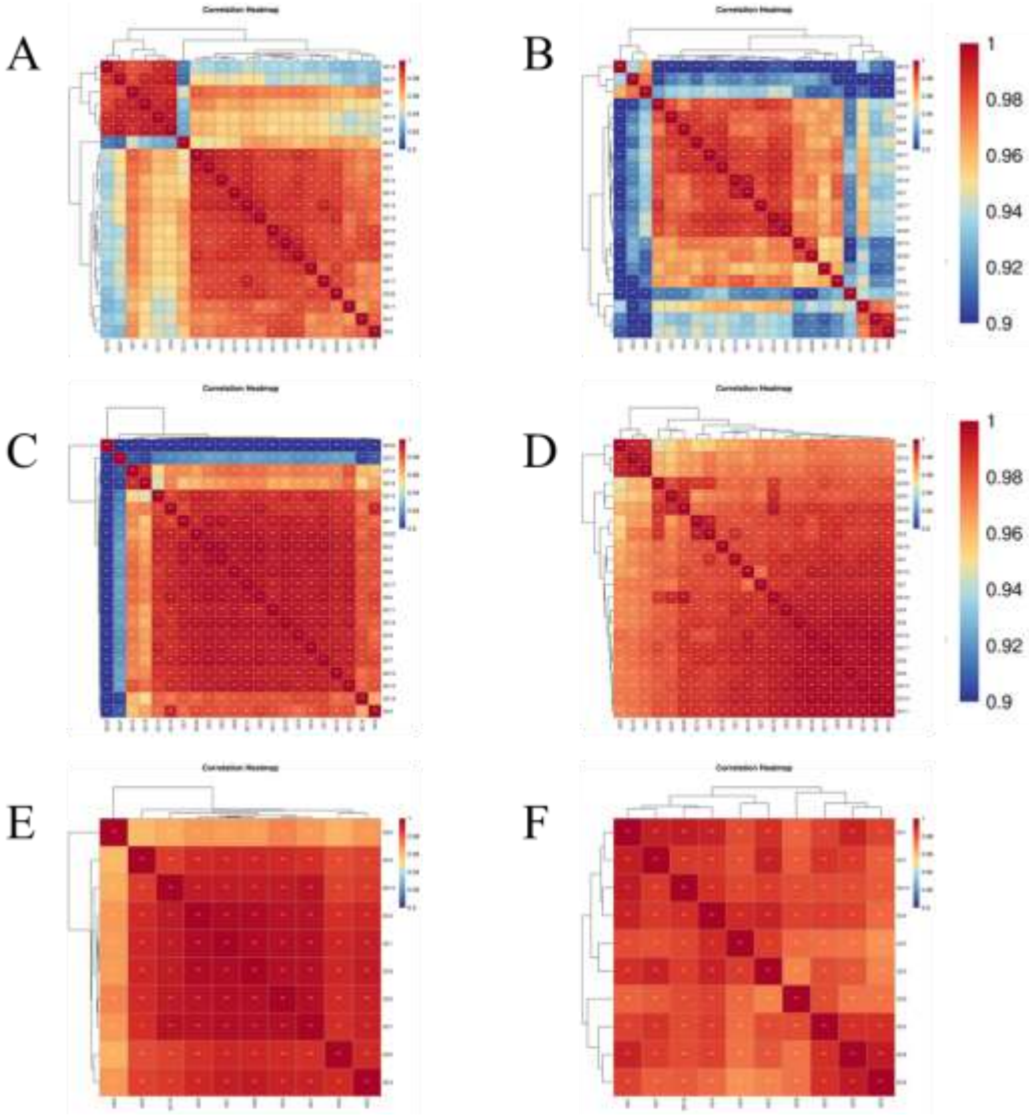

Figure S16. The PCA score plots for all samples containing QC samples. QC samples were closely clustered in the principal component analysis of serum samples ESI^- and ESI^+ (A and B) and stool samples ESI^- and ESI^+ (C and D). QC samples: red dots. PCA, principal component analysis. ESI^-, negative electrospray ionization; ESI^+, positive electrospray ionization.

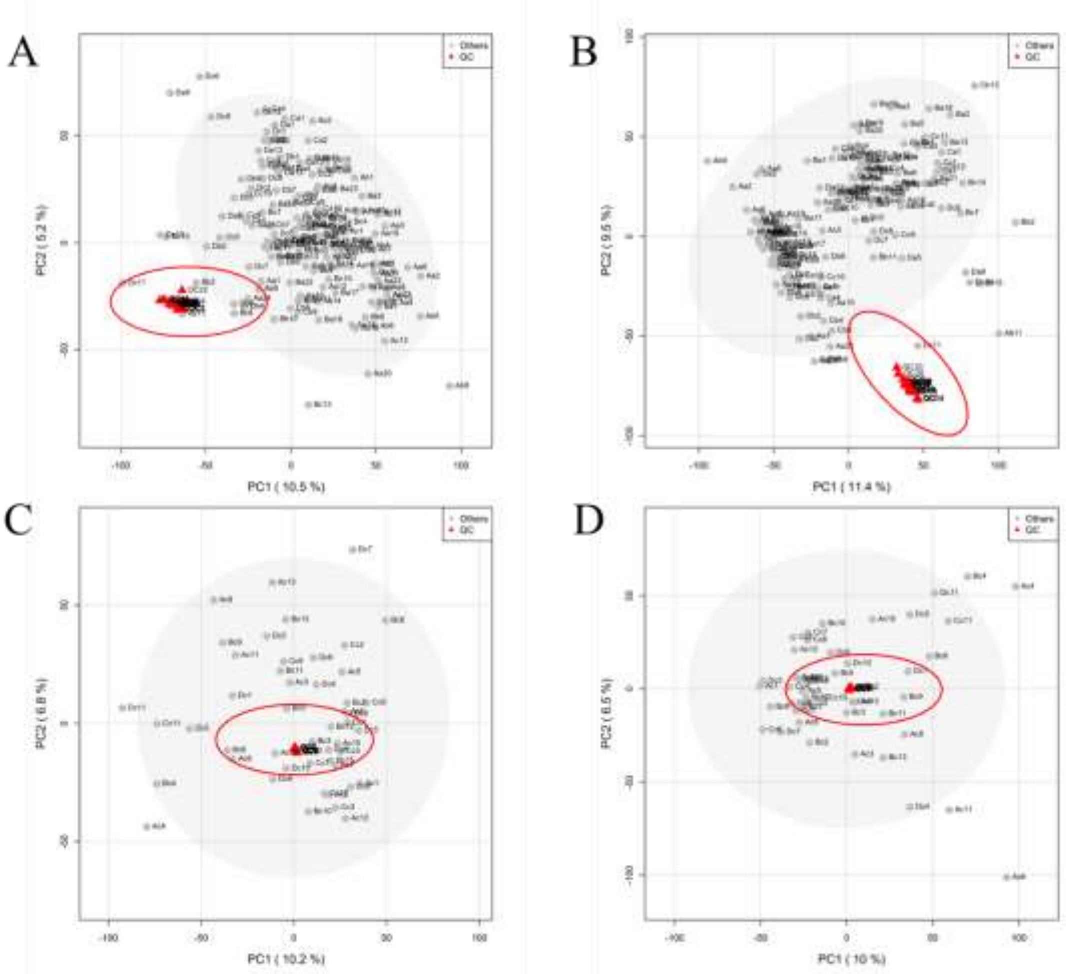

Figure S17. Metabolic alterations in coffee consumption were found in various samples. (A); PLS-DA score plots for serum samples and stool samples in ESI$^-$ model. The habitual coffee drinkers and short-term coffee drinkers are shown in blue, and zero-coffee consumption drinkers are shown in green. X axis and Y axis represent contributions of persons to the first two principal components (PC1 and PC2). (B); Cross-validation plot with a permutation test repeated 200 times. The intercepts of R2= (0.0, 0.39) and Q2= (0.0, –0.77), R2= (0.0, 0.32) and Q2= (0.0, –0.65), R2= (0.0, 0.21) and Q2= (0.0, –0.40) suggest that the PLS-DA model is not overfitting. (C) PLS-DA score plots for serum samples and stool samples in ESI$^+$ model. The habitual coffee drinkers and short-term coffee drinkers are shown in blue, and noncoffee drinkers are shown in green. X axis and Y axis represent contributions of persons to the first two principal components (PC1 and PC2). (D) Cross-validation plot with a permutation test repeated 200 times. The intercepts of R2= (0.0, 0.28) and Q2= (0.0, –0.56), R2= (0.0, 0.34) and Q2= (0.0, –0.50), R2= (0.0, 0.34) and Q2= (0.0, –0.62) suggest that the PLS-DA model is not overfitting. NC, zero-coffee consumption drinkers. Coffee, coffee drinkers. Day_0: the subjects before coffee consumption. Day_30: the subjects after coffee consumption. ESI$^-$, negative electrospray ionisation; ESI$^+$, positive electrospray ionisation; PLS-DA, partial least squares discrimination analysis.

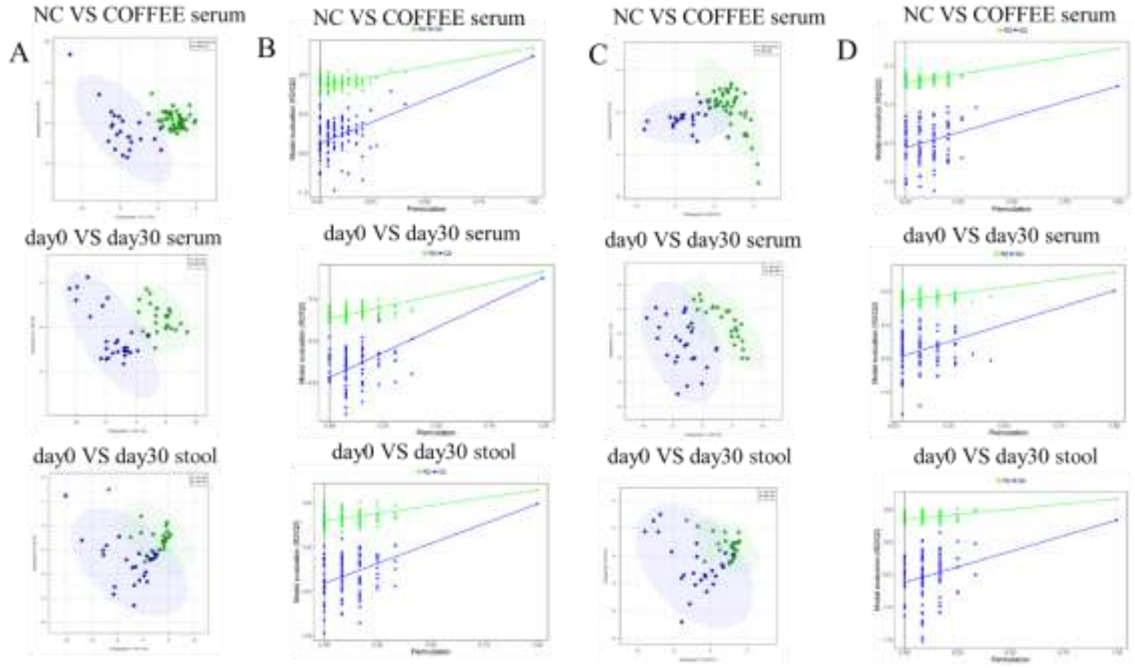

Table S1. A total of 117 metabolite displaying significant differences in abundance between the habitual coffee consumers and zero-coffee consumption consumers.

varID	Compounds	old Change(F(	log2(FC)	p(T test)	ɔgeneityVariaı	VIP
V444	2-Methylpyridine	86.127	6.4284	6.72E-06	8.54E-58	3.149629217
V464	1H-Pyrrole-2-carboxaldehyde	6.4942	2.6991	2.26E-07	1.60E-38	3.513902885
V743	L-Pipecolic acid	1.533	0.61637	0.027373	3.20E-11	1.692708731
V944	(E)-4-(Trimethylammonio)but-2-en	2.8432	1.5075	3.32E-08	0.000373812	3.836655218
V1238	Gabapentin (JAN/USAN/INN)	1.6057	0.68317	0.039541	0.014178396	1.522556273
V1328	L-Canavanine	0.52547	-0.92832	0.00056502	0.001309084	2.508056851
V1369	1,7-Dimethylxanthine	8.8193	3.1407	2.91E-13	2.56E-07	4.538106166
V1370	Theophylline	4.3183	2.1105	4.99E-06	0.002127603	3.153242341
V1411	1-Methyluric Acid	2.556	1.3539	1.37E-08	3.01E-12	3.899682692
V1579	9-Phenanthrol	13.905	3.7975	1.18E-07	3.00E-20	3.586448018
V1580	Cnidilide	0.65243	-0.6161	0.0051323	0.000285797	2.056707464
V1647	4-Phenethylphenol	3.7658	1.913	3.19E-06	0.000699804	3.262025646
V1935	Atrazine	1.614	0.6906	0.041407	0.020178518	1.610410065
V1944	Bisnorbiotin	1.7897	0.83972	0.019579	0.000729068	1.709042724
V2013	Hispolone	2.5435	1.3468	8.31E-05	0.254135014	2.836565292
V2167	Euxanthone	0.54663	-0.87137	0.0029348	0.003306744	2.238360375
V2820	Oil Orange SS	0.13715	-2.8661	0.036591	7.43E-16	1.808992034
V2827	9Z,12Z,15Z-Octadecatrienal	0.61998	-0.6897	0.021235	3.09E-06	1.708895345
V2985	N,N'-Diacetylbenzidine	2.3052	1.2049	0.0091212	0.111841385	1.908813998
V3260	Asn Phe	1.5337	0.61704	0.024169	2.08E-07	1.701899547
V3464	Benzo[a]pyrene-7,8-diol	0.60337	-0.72889	0.037471	0.00426496	1.540266095
V4005	4-Carboxy-4'-sulfoazobenzene	2.5526	1.352	0.046335	1.81E-14	1.534249323
V4662	7-[(2E)-5-hydroxy-3,7-dimethyloctа	0.65621	-0.60777	0.002256	0.496582228	2.245245682
V5343	MG(0:0/18:1(9Z)/0:0)	0.55875	-0.83974	0.032354	5.48E-05	1.598413681
V5843	Riboflavin B2	1.638	0.71198	0.029943	0.145537361	1.679885531
V5852	5α-Androstane-3β,17β-diol diacetat	0.54144	-0.88512	0.010426	2.27E-05	1.89872616
V5866	Karakoline	0.53278	-0.90838	0.012046	1.88E-05	1.867139819
V5893	JWH 210-d9	0.49211	-1.0229	0.0058574	9.60E-05	2.028877246
V5894	2-Arachidonoylglycerol	0.63086	-0.66461	0.044788	0.000156631	1.481951399
V5901	Prostaglandin F1α	1.5359	0.61908	0.049524	1.52E-05	1.498934644
V5924	Leukotriene B4 ethanolamide	0.58814	-0.76576	0.022839	6.29E-05	1.696020867
V6012	Ammoresinol	1.7319	0.79236	0.021156	4.03E-06	1.767695447
V6050	SC-58125	3.2102	1.6827	0.03477	1.68E-13	1.599964022
V7325	cis-(Z)-Flupenthixol dihydrochlorid	0.64551	-0.63149	0.0039998	0.009386943	2.194117274
V7422	2-Butenoic acid, 2-methyl-, (4R,6R	0.55813	-0.84133	0.021333	1.66E-06	1.737524466
V7560	(2alpha,3beta,5alpha,8xi,14xi,25S)-	0.56906	-0.81334	0.037216	7.22E-06	1.559229357
V7855	Delavirdine	0.5794	-0.78738	0.00052876	0.002444397	2.558021819
V8412	Triamcinolone 16,21-diacetate	0.65623	-0.60772	0.0098076	0.143686981	2.038871838
V8579	Stigmatellin Y	0.58457	-0.77454	0.021967	0.00050358	1.742156673
V9627	Trilobolide	0.62239	-0.6841	0.038074	0.245161571	1.558480683
V10211	10-Deacetylbaccatin III	0.61823	-0.69379	0.0036911	0.169109911	2.25554985
V11419	13-Deoxytedanolide	1.5218	0.60575	0.00432	0.363307905	2.129878901
V11783	DG(18:4(6Z,9Z,12Z,15Z)/18:3(9Z,	0.62388	-0.68066	0.006121	0.066749668	1.986713019
V11829	DG(18:3(9Z,12Z,15Z)/18:3(9Z,12Z	0.58367	-0.77678	0.017659	0.047321697	1.768306704
V12348	DG(18:3(9Z,12Z,15Z)/20:5(5Z,8Z,	0.44188	-1.1783	0.0048393	0.001572022	2.053803585
V12374	Purpureacin-1	0.59732	-0.74344	5.66E-05	0.141450246	2.829500021
V12509	OXONITINE	0.58382	-0.7764	0.01758	0.303129079	1.731208351
V12722	Disinomenine	0.64752	-0.627	0.030102	0.009330562	1.62169488
V13054	SM(d18:0/14:0)	1.5214	0.60539	0.00099477	0.008999153	2.356675046
V13116	Fucoxanthin	0.26142	-1.9356	0.024459	9.91E-07	1.762136031
V471	Resorcinol	2.1307	1.0913	0.0012373	0.112691325	2.179190061
V638	2,3-Dimethylsuccinic Acid	2.5261	1.3369	0.0015599	1.43E-24	2.11247311
V794	Acetanilide	1.8773	0.90866	0.0042426	0.013884692	1.95884847
V976	Phenoxyacetic acid	4.7858	2.2587	2.01E-09	2.25E-11	3.629414061
V1186	Perillic acid	0.57899	-0.78838	0.0016153	0.021191241	2.112345891
V1189	3-Methylxanthine	2.9294	1.5506	0.00045554	1.11E-08	2.322832578
V1202	1-Methylxanthine	5.1548	2.3659	5.97E-08	0.395533173	3.392889677
V1237	N-(2-Furoyl)glycine	9.7382	3.2837	0.0050774	4.50E-28	1.901245536
V1242	8-Methyl-6-nonenoic acid	0.61522	-0.70082	0.024668	0.000204752	1.548786054
V1362	Hippuric acid	1.6606	0.73174	0.019868	0.188294703	1.565522714

V1371	Theophylline	2.2912	1.1961	0.000195	0.000223499	2.462374463
V1376	1,7-Dimethylxanthine	6.2654	2.6474	1.52E-13	4.38E-06	4.204554895
V1524	Hydroquinone sulfate	2.2758	1.1864	0.0033762	0.144001341	1.967708978
V1531	Glycylglycylglycine	1.5178	0.60199	0.017014	0.055451422	1.706757496
V1557	Sedoheptulose anhydride	24.99	4.6433	5.48E-06	3.33E-25	2.901781513
V1599	3-Hydroxyhippuric acid	1.9067	0.93107	0.035346	1.07E-07	1.515266985
V1608	3,7-Dimethyluric acid	3.9469	1.9807	9.03E-10	2.66E-06	3.73129444
V1645	5-Acetylamino-6-amino-3-methylur	4.0959	2.0342	1.24E-06	0.000394111	3.121621997
V1814	1,3,7-Trimethyluric acid	1.5086	0.59317	0.017819	0.177056928	1.613491181
V1948	Tyrosol 4-sulfate	5.758	2.5256	0.00059677	1.94E-23	2.303910685
V2163	Diisopropyl adipate	2.8841	1.5281	0.02382	1.59E-11	1.683808262
V2218	Coixinden B	0.60287	-0.73007	0.0035701	0.100851657	1.980814117
V2487	4-methoxy-3-(sulfooxy)benzoic aci	2.1628	1.1129	0.0055705	0.730473329	1.908910878
V2498	Fludioxinil	1.8542	0.89081	0.0036223	0.014194381	1.984738463
V2508	Pyrimethamine	1.8134	0.85871	3.49E-08	0.007409058	3.437851088
V2768	Dl-Cystine	6.7251	2.7496	0.029075	2.03E-27	1.602601105
V2798	Nortriptyline	2.3629	1.2405	3.48E-05	0.001230284	2.794579482
V2832	3-Methoxy-4-Hydroxyphenylethyle	6.3338	2.6631	1.90E-13	8.15E-06	4.201438038
V2916	Inosine	2.8251	1.4983	0.031185	6.36E-11	1.613888638
V2946	2'-O-Methylisoliquiritigenin	2.2857	1.1927	0.034436	2.07E-06	1.550847929
V3031	Ferulic acid 4-sulfate	1.9272	0.94653	0.0083812	0.001621438	1.889506095
V3072	6-Phosphogluconic acid	3.1704	1.6647	0.0053508	1.52E-16	2.007338824
V3169	β-Linoleic acid	0.39022	-1.3576	0.024369	0.000163706	1.579872577
V3255	Physcion	0.61325	-0.70545	0.041744	1.77E-10	1.382723148
V3411	Etrimfos	5.1628	2.3681	0.0071404	3.01E-35	1.834700279
V3574	Myrigalone E	0.60438	-0.72648	0.014766	0.093640811	1.695148644
V3625	Retinyl ester	0.62574	-0.67636	0.031667	0.019757833	1.467177265
V3656	Thymidine 3,5-cyclic monophospha	3.3439	1.7415	0.0091741	1.83E-09	1.853204869
V3829	Phytanate	2.4617	1.2997	0.0045379	5.47E-18	1.891407799
V3852	Icosanoic acid	2.4554	1.2959	0.0053139	1.21E-16	1.86168905
V4344	Prostaglandin D2	71.089	6.1515	3.19E-11	2.25E-42	3.935800022
V4603	(1R,3S,4S,6R)-6,9-Dihydroxyfench	3.3616	1.7492	0.03537	6.62E-20	1.692310821
V4685	(5Z,13E)-9α-Hydroxy-11,15-dioxo	9.3546	3.2257	0.0001533	3.27E-20	2.497152959
V4860	Prednisone	0.3925	-1.3492	0.042999	1.68E-05	1.389689743
V5135	Dihydroferulic acid 4-O-glucuronid	2.8579	1.515	0.00413	5.41E-09	1.991890409
V5406	19-hydroxy-17-oxoandrost-5-en-3-[	0.53833	-0.89344	0.012216	0.000131864	1.691181319
V5523	Quassin	0.61259	-0.70702	0.029861	0.00602512	1.463421454
V5857	Prednisolone 21-acetate	14.001	3.8074	7.50E-10	4.93E-28	3.727318099
V5954	3α,7α-Dihydroxy-12-oxo-5β-cholan	0.46673	-1.0993	0.042975	8.09E-09	1.407020725
V6043	7,11,12-Triacetoxycoumestan	2.5827	1.3689	0.027457	2.53E-06	1.52604469
V6145	Eplerenone	0.63252	-0.66081	9.66E-05	0.041136823	2.52696703
V6449	LPG 12:0	0.52092	-0.94086	0.0014445	0.000115252	2.102652414
V6998	methyl 3-(3,4-dihydroxyphenyl)-2-[	23.111	4.5305	8.37E-07	2.09E-30	3.202625496
V7457	PG 14:0	0.58607	-0.77085	0.0077368	0.009130145	1.78945539
V7602	Lucidenic acid C	0.41462	-1.2701	0.029349	1.30E-05	1.534513767
V8102	LysoPE(20:5(5Z,8Z,11Z,14Z,17Z)/	4.4658	2.1589	0.038166	8.45E-40	1.446665578
V8491	Cinnamoside	0.6209	-0.68757	0.027669	0.000682781	1.50847301
V8510	Ganoderiol C	0.66511	-0.58834	0.02265	3.63E-08	1.568848533
V8849	PS 18:3	0.64803	-0.62587	0.0015288	0.00030823	2.108295058
V8898	PS 18:2	0.53367	-0.90598	0.044787	0.003198187	1.386169414
V9317	Cer-NDS d32:1	1.5035	0.58833	0.0040036	0.001020785	2.04276951
V9931	DGDG 10:0	0.65454	-0.61144	0.020487	0.001691619	1.617874123
V11454	PAz-PC	0.63264	-0.66053	0.042577	0.00955882	1.54361788
V11584	α-N-Acetylneuraminyl-2,6-β-D-gal	0.65763	-0.60464	0.018029	0.003499909	1.668242305
V11876	SM d30:1	1.5196	0.60366	0.0021819	0.168848201	2.061939435
V12313	PE(18:4(6Z,9Z,12Z,15Z)/P-18:1(11	1.6167	0.69305	0.016052	0.000197087	1.628093611
V12343	PC 28:0; PC 14:0-14:0	2.0971	1.0684	0.015655	0.00132665	1.626293218

Table S2. KEGG metabolic pathway analysis

a. KEGG metabolic pathway analysis of annotated metabolites in the habitual coffee consumers and zero-coffee consumption consumers

	pathNames	pathIds	Total	Hits	Raw p	Impact
1	Caffeine metabolism	hsa00232	10	4	0.00048978	1
2	Glycerophospholipid metabolism	hsa00564	36	6	0.003115	0.27989
3	Linoleic acid metabolism	hsa00591	5	2	0.015958	1
4	Glycerolipid metabolism	hsa00561	16	2	0.14259	0.21028
5	Riboflavin metabolism	hsa00740	4	1	0.15763	0.5
6	Pentose phosphate pathway	hsa00030	22	2	0.23479	0.14137
7	Vitamin B6 metabolism	hsa00750	9	1	0.32062	0.4902
8	Phenylalanine metabolism	hsa00360	10	1	0.34928	0
9	alpha-Linolenic acid metabolism	hsa00592	13	1	0.4283	0
10	Biosynthesis of unsaturated fatty acids	hsa01040	36	2	0.45092	0
11	Arachidonic acid metabolism	hsa00590	36	2	0.45092	0.02023
12	Glycosylphosphatidylinositol (GPI)-anchor biosynthesis	hsa00563	14	1	0.45248	0.00399
13	Retinol metabolism	hsa00830	17	1	0.51913	0.15464
14	Sphingolipid metabolism	hsa00600	21	1	0.59571	0
15	Lysine degradation	hsa00310	25	1	0.66026	0
16	Pyrimidine metabolism	hsa00240	39	1	0.81586	0.08886
17	Purine metabolism	hsa00230	65	1	0.94187	0.00234
18	Metabolism of xenobiotics by cytochrome	hsa00980	68	1	0.94918	0.05612

b. KEGG metabolic pathway analysis of annotated metabolites in subjects after ground coffee consumption

	pathNames	pathIds	Total	Hits	Raw p	Impact
1	Glycerophospholipid metabolism	hsa00564	36	7	0.00014191	0.47442
2	Caffeine metabolism	hsa00232	10	4	0.00022007	1
3	Glycerolipid metabolism	hsa00561	16	3	0.015429	0.22274
4	Sphingolipid metabolism	hsa00600	21	4	0.035955	0.13996
5	Glycosylphosphatidylinositol (GPI)-anchor biosynthesis	hsa00563	14	2	0.080303	0.00399
6	Arachidonic acid metabolism	hsa00590	36	3	0.12218	0.04046
7	Linoleic acid metabolism	hsa00591	5	1	0.15986	0
8	Phosphatidylinositol signaling system	hsa04070	28	2	0.248	0.09899
9	Vitamin B6 metabolism	hsa00750	9	1	0.26945	0.4902
10	alpha-Linolenic acid metabolism	hsa00592	13	1	0.36497	0
11	Butanoate metabolism	hsa00650	15	1	0.40803	0.03175
12	Alanine, aspartate and glutamate metabolism	hsa00250	28	1	0.62578	0.04808
13	Porphyrin and chlorophyll metabolism	hsa00860	30	1	0.6514	0.05288
14	Inositol phosphate metabolism	hsa00562	30	1	0.6514	0.0777
15	Biosynthesis of unsaturated fatty acids	hsa01040	36	1	0.71836	0
16	Arginine and proline metabolism	hsa00330	38	1	0.73774	0.00039

Table S3. The Correlation analysis of $CD8^+$ $CD45RA^+$ cells and changed metabolites

Metabolites	Cell	rho	pvalue	relation
Ferulic acid 4-sulfate	CD8+CD45RA+ T cell	-0.416580984	0.00155807	negtive
1,3,7-Trimethyluric acid	CD8+CD45RA+ T cell	-0.303407472	0.024333779	negtive
PS 18:2	CD8+CD45RA+ T cell	-0.302505547	0.024781537	negtive
3-Methylxanthine	CD8+CD45RA+ T cell	-0.270829952	0.045506356	negtive
N-(2-Furoyl)glycine	CD8+CD45RA+ T cell	-0.253657305	0.061670886	negtive
Acetanilide	CD8+CD45RA+ T cell	-0.219492397	0.107373179	negtive
Hippuric acid	CD8+CD45RA+ T cell	-0.207875607	0.127776972	negtive
Tyrosol 4-sulfate	CD8+CD45RA+ T cell	-0.20148998	0.140173831	negtive
Theophylline	CD8+CD45RA+ T cell	-0.189079496	0.166802816	negtive
DGDG 10:0	CD8+CD45RA+ T cell	0.166783917	0.223589578	positive
Prostaglandin D2	CD8+CD45RA+ T cell	-0.1657014	0.226652203	negtive
1-Methylxanthine	CD8+CD45RA+ T cell	-0.159568519	0.244551669	negtive
Prednisone	CD8+CD45RA+ T cell	-0.151920198	0.268192867	negtive
Prednisolone 21-acetate	CD8+CD45RA+ T cell	-0.148384653	0.279620246	negtive
Cer-NDS d32:1	CD8+CD45RA+ T cell	0.141530025	0.302678769	positive
Quassin	CD8+CD45RA+ T cell	-0.126666306	0.356776372	negtive
1,7-Dimethylxanthine	CD8+CD45RA+ T cell	-0.107509425	0.43464593	negtive
2,3-Dimethylsuccinic Acid	CD8+CD45RA+ T cell	-0.084780923	0.538277362	negtive
8:4(6Z,9Z,12Z,15Z)/P-18:1(1	CD8+CD45RA+ T cell	0.078972527	0.566563489	positive
SM d30:1	CD8+CD45RA+ T cell	0.074318596	0.589715342	positive
Resorcinol	CD8+CD45RA+ T cell	-0.056640872	0.681261964	negtive
8-Methyl-6-nonenoic acid	CD8+CD45RA+ T cell	0.055161715	0.689157479	positive
PG 14:0	CD8+CD45RA+ T cell	0.054404098	0.69321436	positive
PC 28:0; PC 14:0-14:0	CD8+CD45RA+ T cell	0.022728503	0.869173277	positive
Perillic acid	CD8+CD45RA+ T cell	-0.022475964	0.870614393	negtive
PS 18:3	CD8+CD45RA+ T cell	-0.019517651	0.887525901	negtive
Nortriptyline	CD8+CD45RA+ T cell	-0.019445497	0.88793903	negtive
Phenoxyacetic acid	CD8+CD45RA+ T cell	0.007576168	0.956220854	positive
ydroferulic acid 4-O-glucuroı	CD8+CD45RA+ T cell	-0.001803849	0.989571596	negtive
PAz-PC	CD8+CD45RA+ T cell	-0.000288616	0.99833141	negtive

Table S4. GO enrichment analyses of DEGs in subjects after ground coffee consumption.

ONTOLO	Description	pvalue	qvalue	Count
BP	cytokine-mediated signaling pathway	1.18E-11	5.11E-08	54
BP	positive regulation of cytokine production	7.84E-11	1.70E-07	52
BP	leukocyte migration	2.65E-10	3.76E-07	44
BP	regulation of inflammatory response	3.47E-10	3.76E-07	45
BP	cell chemotaxis	5.88E-10	5.10E-07	39
BP	myeloid leukocyte migration	2.25E-09	1.63E-06	31
BP	positive regulation of response to external stimulus	2.89E-09	1.79E-06	46
BP	neutrophil migration	4.98E-09	2.70E-06	22
BP	leukocyte chemotaxis	6.67E-09	3.21E-06	31
BP	regulation of immune effector process	7.69E-09	3.34E-06	39
BP	granulocyte migration	4.19E-08	1.51E-05	23
BP	pattern recognition receptor signaling pathway	4.29E-08	1.51E-05	25
BP	myeloid leukocyte activation	4.57E-08	1.51E-05	29
BP	response to molecule of bacterial origin	5.05E-08	1.51E-05	39
BP	regulation of pattern recognition receptor signaling pathway	5.22E-08	1.51E-05	19
BP	regulation of leukocyte mediated immunity	6.14E-08	1.66E-05	29
BP	response to lipopolysaccharide	1.00E-07	2.56E-05	37
BP	positive regulation of defense response	1.69E-07	3.76E-05	32
BP	positive regulation of interleukin-8 production	1.71E-07	3.76E-05	14
BP	regulation of interleukin-8 production	1.74E-07	3.76E-05	18
BP	interleukin-8 production	2.02E-07	4.18E-05	18
BP	positive regulation of interleukin-6 production	2.25E-07	4.43E-05	17
BP	regulation of response to biotic stimulus	2.68E-07	5.06E-05	35
BP	interleukin-6 production	3.20E-07	5.55E-05	23
CC	ficolin-1-rich granule	6.35E-10	1.50E-07	29
CC	secretory granule membrane	7.04E-10	1.50E-07	39
CC	tertiary granule	3.00E-07	4.28E-05	23
CC	ficolin-1-rich granule membrane	9.88E-07	0.000106	13
CC	secondary lysosome	2.74E-06	0.000234	7
CC	endocytic vesicle	4.18E-06	0.000298	33
CC	stress fiber	5.97E-06	0.000319	13
CC	contractile actin filament bundle	5.97E-06	0.000319	13
CC	actin filament bundle	2.30E-05	0.00109	13
CC	actomyosin	2.63E-05	0.001125	13
MF	immune receptor activity	7.49E-11	5.63E-08	27
MF	GTPase regulator activity	6.73E-08	1.69E-05	48
MF	nucleoside-triphosphatase regulator activity	6.73E-08	1.69E-05	48
MF	GTPase activator activity	1.45E-06	0.000272	43
MF	cytokine receptor activity	3.25E-06	0.000472	16
MF	inhibitory MHC class I receptor activity	4.40E-06	0.000472	6
MF	Toll-like receptor binding	4.40E-06	0.000472	6
MF	superoxide-generating NADPH oxidase activator activity	2.95E-05	0.002586	5
MF	guanyl-nucleotide exchange factor activity	3.10E-05	0.002586	24
MF	MHC class I receptor activity	4.90E-05	0.003682	6

Table S5. KEGG enrichment analyses of DEGs in subjects after ground coffee consumption.

ID	Description	GeneRatio	BgRatio	pvalue	p.adjust	qvalue	Count
hsa04380	Osteoclast differentiation	25/397	128/8209	1.55E-09	3.20E-07	2.72E-07	25
hsa04060	Cytokine-cytokine receptor interaction	40/397	295/8209	2.17E-09	3.20E-07	2.72E-07	40
hsa04062	Chemokine signaling pathway	27/397	192/8209	4.77E-07	3.52E-05	2.99E-05	27
hsa05417	Lipid and atherosclerosis	28/397	215/8209	1.43E-06	8.43E-05	7.16E-05	28
hsa04668	TNF signaling pathway	18/397	112/8209	6.09E-06	0.0003	0.000254	18
hsa04666	Fc gamma R-mediated phagocytosis	15/397	97/8209	5.77E-05	0.002134	0.001813	15
hsa04657	IL-17 signaling pathway	14/397	94/8209	0.000154	0.004532	0.003849	14
hsa04630	JAK-STAT signaling pathway	19/397	166/8209	0.000399	0.009797	0.00832	19
hsa04064	NF-kappa B signaling pathway	14/397	104/8209	0.000451	0.010241	0.008697	14
hsa04620	Toll-like receptor signaling pathway	13/397	104/8209	0.001444	0.026622	0.022609	13
hsa04662	B cell receptor signaling pathway	11/397	82/8209	0.001862	0.032309	0.027438	11
hsa04010	MAPK signaling pathway	26/397	294/8209	0.002052	0.033629	0.028559	26
hsa03320	PPAR signaling pathway	10/397	75/8209	0.003099	0.043537	0.036974	10

Table S6. GSEA enrichment analyses of DEGs in subjects after ground coffee consumption.

ID	Description	setSize	·ichmentSc	NES	pvalue
1	KEGG_RIBOSOME	82	0.649318	2.555213	0.000518
2	KEGG_OXIDATIVE_PHOSPHORYLATION	108	0.456799	1.873583	0.000605
3	KEGG_PARKINSONS_DISEASE	109	0.460734	1.89665	0.000605
4	KEGG_HUNTINGTONS_DISEASE	148	0.369584	1.583831	0.000773
5	KEGG_TYPE_I_DIABETES_MELLITUS	35	0.543977	1.80814	0.002153
6	ESTINAL_IMMUNE_NETWORK_FOR_IGA_PR(	36	0.508115	1.699553	0.006519
7	KEGG_ALZHEIMERS_DISEASE	137	0.329032	1.396236	0.007278
8	KEGG_NITROGEN_METABOLISM	18	-0.64974	-1.619547	0.011032
9	_CYTOKINE_CYTOKINE_RECEPTOR_INTERA(	170	0.303299	1.328754	0.011083
10	KEGG_TASTE_TRANSDUCTION	25	-0.587851	-1.57605	0.016893

Table S7. Metabolites and their matched metabolic genes.

English Name	As_substrate_gene	As_product_gene
Bilirubin	UGT1A1/UGT1A3/UGT1A4/U	BLVRA/BLVRB/SLCO1B1/CP/SLCO1A2/
Sedoheptulose anhydride	SHPK/KHK	NA
2-Arachidonoylglycerol	PTGS1/PTGS2	PPAP2A/PPAP2B
2-Methylpyridine	PDXK	PDXP/PNPO
SM(d18:0/20:2(11Z,14Z))	NA	SMPD1/SMPD2/SMPD3/SMPD4/ENPP4
BMP 23:3	LIAS/LIPT2	NA
9(S)-HPETE	GPX1/GPX3/GPX4/GPX5/GP	ALOX12/PRDX6
1,7-Dimethylxanthine	CYP1A2,CYP1A6,XDH	CYP1A2
Cer 36:1	B3GNT2/B3GNT3	NA
Galactosylceramide	ARSA/GAL3ST1/GALC/GLA	GLA/GAL3ST1
Eicosapentaenoic acid	ACSL1/SLC27A2/ACSL3/AC	CEL/LIPA/PTGS2/PTGS1/TBCB
11,12-EET	ACAA1/HSD17B10/HADHA/	CYP1A1/CYP1A2/CYP2E1/CYP3A4/CYP2C8
Tetrahydro-11-deoxycortis	ABCB1	CYP21A2
Succinate semialdehyde	ABAT	ALDH5A1
(±)12,13-DiHOME	NA	NA
(±)-Abscisic acid	NA	NA
(±)-Jasmonic acid	NA	NA
(5Z,13E)-9α-Hydroxy-11,1	NA	NA
(6,8-dipropyloctahydroindo	NA	NA
(E)-4-(Trimethylammonio)	NA	NA
12(S)-HHT	NA	NA
12,20-Dioxo-leukotriene B	NA	NA
15,16-DiHODE	NA	NA
1-hexadecanoyl-2-(9Z,12Z-	NA	NA
1-Hexadecanoyl-2-(9Z-octa	NA	NA
1H-Pyrrole-2-carboxaldehy	NA	NA
1-Methyluric Acid	NA	NA
1-Methylxanthine	NA	NA
1-O-Desmethyltetrabenazir	NA	NA
1-Pyrroline-2-carboxylate	NA	DAO
2-Amino-5-phenylpyridine	NA	NA
2-bromo-octadecanoic acid	NA	NA
2-Butenoic acid	NA	NA
3,4-Dehydrothiomorpholin	NA	NA
3,7,13,17-O-tetracetoxy-15	NA	NA
3,7-Dimethyluric acid	NA	NA
3-Methoxy-4-Hydroxyphen	NA	NA
3-Methylxanthine	NA	NA
5,6-Dihydroxyprostaglandi	NA	NA
5-Acetylamino-6-amino-3-	NA	NA
5-O-Desmethyldonepezil	NA	NA
5α-Androstane-3β,17β-diol	NA	NA
7,11-Methano-2H-cyclooct	NA	NA
7α,17β-Dihydroxyandrosta-	NA	NA
8-(1,1-DMA)kaempferide	NA	NA
8phiC9SPC	NA	NA
9(S)-HETE	NA	CYP2F1
9Z,12Z,15Z-Octadecatrien	NA	NA
Achimilic acid	NA	NA
Aflatoxin ExB2	NA	NA
Albafuran A	NA	NA
Alternariol	NA	NA
Alverine (INN)	NA	NA
Angiotensin IV	NA	NA
Annosquamosin B	NA	NA

Annuionone B	NA	NA
AS 1-5	NA	NA
baicalein	NA	NA
Biopterin	NA	PCBD1/PCBD2
Bisnorbiotin	NA	NA
Bouillonamide B	NA	NA
Cascarillin	NA	NA
Cer 34:1	NA	NA
Cer-NDS d34:0; Cer-NDS	NA	NA
Cer-NS d34:1	NA	NA
Cer-NS d36:2	NA	NA
Cinerin I	NA	NA
Cinncassiol C2	NA	NA
Cinncassiol D2 glucoside	NA	NA
CL 74:5	NA	NA
Cocamidopropylβine	NA	NA
Cysteinyl-Tyrosine	NA	NA
Deflazacort	NA	NA
DGDG 10:0	NA	NA
DGDG 19:1	NA	NA
EtherPE 36:4e; PE(18:2e/1	NA	NA
FAHFA 20:0; FAHFA 12:(	NA	NA
Faradiol myristate	NA	NA
G1Cer 40:2	NA	NA
Genipin 1-β-gentiobioside	NA	NA
Glutamylvaline	NA	NA
Gluten exorphin B4	NA	NA
Gnididilatin	NA	NA
HexCer-BS d34:2	NA	NA
Homomangiferin	NA	NA
Isocycloneosamandaridine	NA	NA
Isodomedin	NA	NA
JWH 210-d9	NA	NA
Karakoline	NA	NA
Lansine	NA	NA
Leukotriene B4 ethanolami	NA	NA
Longifolenaldehyde	NA	NA
LysoPE(22:5(7Z,10Z,13Z,1	NA	NA
methyl 3-(3,4-dihydroxyph	NA	NA
MG(0:0/18:1(9Z)/0:0)	NA	NA
N-(3-Oxododecanoyl)homo	NA	NA
N,N'-Diacetylbenzidine	NA	NA
N-[(4E,8E)-1,3-dihydroxyo	NA	NA
N-Acetyl-L-methionine	NA	NA
Nap-Thr-OH	NA	NA
Nemonapride	NA	NA
Nicardipine	NA	NA
Nilotinib (USAN/INN)	NA	NA
PA 20:3	NA	NA
PA 20:3; PA 2:0-18:3	NA	NA
PA 41:5	NA	NA
PC(15:0/20:5(5Z,8Z,11Z,1	NA	NA
PC(15:0/22:6(4Z,7Z,10Z,1	NA	NA
PE 22:5	NA	NA
PE 34:2	NA	NA
PE 36:2	NA	NA
PE 36:4	NA	NA

PE 38:4	NA	NA
PE 38:4	NA	NA
PE(16:0/22:6)	NA	NA
PE(16:1(9Z)/P-18:1(11Z))	NA	NA
Phenoxyacetic acid	NA	NA
Physalin L	NA	NA
PI 38:4	NA	NA
Picrasin B	NA	NA
Piperonyl sulfoxide	NA	NA
Prednisolone 21-acetate	NA	NA
Prometon	NA	NA
Prostaglandin D2	NA	NA
PS 36:4	NA	NA
PS 38:4	NA	NA
PS 40:6	NA	NA
Ptaquiloside	NA	NA
Raubasine	NA	NA
Samandarone	NA	NA
SM d38:5	NA	NA
SQMG(0:0/16:1(11Z))	NA	NA
Theophylline	NA	NA
Thionazin	NA	NA
Thromboxane B2	NA	NA
Trp Val	NA	NA
α-N-Acetylneuraminyl-2,6-	NA	NA

Table S8. A total of 139 different metabolites in subjects before and after ground coffee consumption.

varID	Compounds	log2(FC)	p(T test)	VIP
V3884	(±)12,13-DiHOME	-0.62783	0.0028468	1.634640161
V2818	(±)-Abscisic acid	-0.71741	0.031493	1.35543114
V1841	(±)-Jasmonic acid	1.061	1.34E-05	2.516370132
V4685	(5Z,13E)-9α-Hydroxy-11,15-dioxoprosta-5,13-dienoate	7.1771	0.020276	1.326776698
V2354	(6,8-dipropyloctahydroindolizin-5-yl)methanol	0.77652	0.0059758	1.606561244
V944	(E)-4-(Trimethylammonio)but-2-enoate	1.1469	2.72E-08	2.972945958
V1369	1,7-Dimethylxanthine	5.3875	1.85E-06	2.78919701
V1376	1,7-Dimethylxanthine	2.5253	9.68E-07	2.815424915
V4019	11,12-EET	-1.4276	0.0015291	1.698550418
V3155	12(S)-HHT	-1.3907	8.59E-05	2.165301273
V4634	12,20-Dioxo-leukotriene B4	-1.3138	8.45E-05	2.170203201
V3817	15,16-DiHODE	0.6868	0.01234	1.414915006
V13724	1-hexadecanoyl-2-(9Z,12Z-octadecadienoyl)-sn-glycero-3-phospho-D-myo-inositol	1.3686	6.61E-06	2.343336243
V12246	1-Hexadecanoyl-2-(9Z-octadecenoyl)-sn-glycero-3-phosphoethanolamine	1.0176	0.0049544	1.846263837
V464	1H-Pyrrole-2-carboxaldehyde	2.1652	3.41E-07	2.785876195
V1411	1-Methyluric Acid	1.3019	9.21E-06	2.584237954
V1202	1-Methylxanthine	2.5685	0.00089235	1.990109533
V3945	1-O-Desmethyltetrabenazine	-1.3257	0.00058973	2.078732419
V516	1-Pyrroline-2-carboxylate	-1.1841	1.41E-05	2.654327826
V1244	2-Amino-5-phenylpyridine	-0.94476	0.0002239	2.114570282
V5894	2-Arachidonoylglycerol	1.191	0.009722	1.629492171
V5494	2-bromo-octadecanoic acid	-0.70697	0.0020231	1.892330464
V7422	2-Butenoic acid, 2-methyl-, (4R,6R)-3,3,7,9-tetramethyl-6-(3-methyl-1-oxobutoxy)-11-oxotricyclo[5.4.0.0~2,8~]undec-9-en-4-yl ester, (2Z)-	0.70413	0.028489	1.47349807
V444	2-Methylpyridine	6.3772	0.037021	1.221633573
V952	3,4-Dehydrothiomorpholine-3-carboxylate	0.72291	0.0050973	1.688875628
V12751	3,7,13,17-O-tetracetoxy-15'hydroxy-5'O-methylbutyroyloxy-14-oxopremyrsinol	0.63033	0.00070838	2.046806441
V1608	3,7-Dimethyluric acid	2.3859	2.51E-06	2.571844187
V2832	3-Methoxy-4-Hydroxyphenylethyleneglycol Sulfate	2.4735	6.91E-07	2.835765834
V1189	3-Methylxanthine	1.3537	0.00085395	1.937873655
V5527	5,6-Dihydroxyprostaglandin F1a	-0.995	0.0027378	1.624881297
V1645	5-Acetylamino-6-amino-3-methyluracil	3.1545	0.00045539	1.918534615
V5568	5-O-Desmethyldonepezil	-1.7563	0.000814	1.976228036
V5852	5α-Androstane-3β,17β-diol diacetate	0.84124	0.016156	1.571805058

V9728	7,11-Methano-2H-cycloocta[3,4]benzo[1,2-c]pyran-8-acetic acid, alpha-(acetyloxy)-4-(3-furanyl)-1,4,4a,5,6,6a,7,8,9,10,11,12-dodecahydro-4a,7,9,9-tetramethyl-2,10,13-trioxo-, methyl ester, (4R,4aR,7R,8S,11R)-	0.76442	0.0006482	2.059843635
V3905	7α,17β-Dihydroxyandrosta-1,4-dien-3-	-1.2742	0.00036181	2.141949959
V5650	8-(1,1-DMA)kaempferide	-0.60535	0.014117	1.480081199
V3872	8phiC9SPC	0.84691	0.04218	1.289426936
V4972	9(S)-HETE	-1.4057	0.00083691	1.975764805
V4806	9(S)-HPETE	0.75533	0.039089	1.325216315
V2827	9Z,12Z,15Z-Octadecatrienal	0.7561	0.01049	1.636308937
V3119	Achimilic acid	1.4188	0.0027083	1.607160581
V4849	Aflatoxin ExB2	-1.3893	1.92E-06	2.460779339
V5249	Albafuran A	-1.4692	0.0012914	1.725642543
V2734	Alternariol	-0.84511	0.022195	1.392851214
V3183	Alverine (INN)	-1.9308	8.04E-05	2.164923757
V14450	Angiotensin IV	0.67725	8.80E-05	2.287268092
V4056	Annosquamosin B	0.74339	0.0056864	1.723504314
V2052	Annuionone B	0.46393	0.012673	1.52991677
V12212	AS 1-5	0.88703	4.84E-05	2.201018107
V2940	baicalein	0.89344	0.0014825	1.955219687
V11213	Bilirubin	-1.0637	0.0020096	2.001228762
V2319	Biopterin	1.3181	6.86E-09	3.143538048
V1944	Bisnorbiotin	0.86145	0.0027184	1.712877629
V11721	BMP 23:3	1.0276	0.00024539	2.13004286
V12144	Bouillonamide B	-1.2663	0.01798	1.47195826
V5999	Cascarillin	-1.1099	0.0064646	1.495791675
V9914	Cer 34:1	0.53094	1.51E-05	2.216160155
V10498	Cer 36:1	0.77896	1.53E-05	2.237151995
V9967	Cer-NDS d34:0; Cer-NDS d18:0/16:0	0.85708	0.0030531	1.685200399
V8949	Cer-NS d34:1	0.76728	1.23E-05	2.249354032
V10466	Cer-NS d36:2	0.78249	0.00124	1.890903233
V4284	Cinerin I	0.62185	0.0078903	1.642896379
V5543	Cinncassiol C2	-1.3641	0.001145	1.929812069
V9826	Cinncassiol D2 glucoside	0.60999	0.00042185	2.130143463
V12610	CL 74:5	1.1963	0.0038926	1.876425752
V4982	Cocamidopropylβine	0.57812	0.014991	1.440875815
V3247	Cysteinyl-Tyrosine	-0.62193	0.00019392	2.133375013
V7517	Deflazacort	0.58318	0.0002838	2.158973327
V9931	DGDG 10:0	-1.4206	0.014571	1.341193111
V13855	DGDG 19:1	0.53977	0.00058325	2.075352005
V3906	Eicosapentaenoic acid	-1.5029	0.00084284	1.976262424
V12377	EtherPE 36:4e; PE(18:2e/18:2)	0.61464	0.0032335	1.939332044
V4517	FAHFA 20:0; FAHFA 12:0/8:0	-0.80448	0.020397	1.337303492
V12643	Faradiol myristate	-1.1743	0.0084662	1.626812238
V13642	G1Cer 40:2	0.66568	0.00021642	1.940761514
V11985	Galactosylceramide	0.83611	0.00015207	2.063584658
V10366	Genipin 1-β-gentiobioside	-0.63491	0.012994	1.512922439
V2466	Glutamylvaline	-0.87326	0.0001707	2.045203323
V8765	Gluten exorphin B4	-1.6169	0.00051521	1.971494921
V12635	Gnididilatin	-1.693	0.003328	1.791651128
V12172	HexCer-BS d34:2	0.8688	0.0010966	1.810233427
V6621	Homomangiferin	4.4679	0.039848	1.186759889
V5053	Isocycloneosamandaridine	-2.5188	0.0062744	1.675160525
V6271	Isodomedin	-1.3514	0.0010518	1.990456318
V5893	JWH 210-d9	0.84807	0.022348	1.530052209
V5866	Karakoline	0.84046	0.018496	1.55070441
V2145	Lansine	2.1626	0.0012231	1.842990348
V5924	Leukotriene B4 ethanolamide	0.76519	0.03518	1.483466731

V2024	Longifolenaldehyde	-0.48775	0.0071389	1.582338042
V8720	LysoPE(22:5(7Z,10Z,13Z,16Z,19Z)/0:0)	0.60308	0.0036208	1.669816732
V6998	methyl 3-(3,4-dihydroxyphenyl)-2-[(2,2-dimethylchromen-6-yl)methyl]-4-methoxy-5-oxofuran-2-carboxylate	10.223	4.46E-13	3.424914381
V5343	MG(0:0/18:1(9Z)/0:0)	0.7118	0.032441	1.503285136
V3505	N-(3-Oxododecanoyl)homoserine lactone	-0.96666	0.025854	1.54781884
V2985	N,N'-Diacetylbenzidine	2.6522	0.042028	1.284669339
V9973	N-[(4E,8E)-1,3-dihydroxyoctadeca-4,8-dien-2-yl]hexadecanamide	0.79377	0.00017084	2.130883277
V1538	N-Acetyl-L-methionine	-0.65952	0.0079764	1.440621222
V6343	Nap-Thr-OH	-1.3421	4.12E-05	2.241314931
V6130	Nemonapride	0.63476	0.00023715	2.193605952
V7659	Nicardipine	1.3803	2.57E-06	2.537100389
V8760	Nilotinib (USAN/INN)	-4.8279	0.00056247	1.941138055
V7558	PA 20:3	-2.2957	0.014746	1.346115771
V7549	PA 20:3; PA 2:0-18:3	-2.39	0.0046379	1.535906863
V12923	PA 41:5	1.2547	0.01164	1.461156497
V12934	PC(15:0/20:5(5Z,8Z,11Z,14Z,17Z))	1.0441	0.0010126	1.965597285
V13251	PC(15:0/22:6(4Z,7Z,10Z,13Z,16Z,19Z))	0.77488	0.0048482	1.788443433
V9036	PE 22:5	-0.80111	0.0088403	1.546309383
V12204	PE 34:2	1.0043	0.0021457	1.837148605
V12638	PE 36:2	1.0858	0.0033136	1.854773211
V12583	PE 36:4	0.8757	0.00082829	2.028153418
V12963	PE 38:4	0.97356	0.001806	1.995050837
V12967	PE 38:4	0.94467	0.0016017	1.889728543
V12900	PE(16:0/22:6)	0.77985	0.011715	1.723499506
V11984	PE(16:1(9Z)/P-18:1(11Z))	0.65443	0.00031582	2.035970041
V976	Phenoxyacetic acid	2.2873	7.08E-09	3.020491672
V8735	Physalin L	-1.3404	1.12E-05	2.314770806
V14134	PI 38:4	-0.6844	0.01992	1.4563275
V5841	Picrasin B	0.52717	0.00049859	2.10273504
V4486	Piperonyl sulfoxide	-1.1818	0.0010843	1.954052174
V5857	Prednisolone 21-acetate	5.3571	1.44E-09	2.959685728
V2091	Prometon	1.1247	0.0035099	1.905289438
V4344	Prostaglandin D2	9.3452	6.87E-12	3.284259488
V13155	PS 36:4	0.75101	0.011177	1.509280544
V13471	PS 38:4	0.7729	0.0019189	1.740304455
V13731	PS 40:6	0.67215	4.88E-06	2.322620708
V6424	Ptaquiloside	0.61192	0.00011344	2.251639415
V13494	Raubasine	-1.6866	0.0042278	1.738648881
V3944	Samandarone	-1.4381	0.00091457	1.964525087
V1557	Sedoheptulose anhydride	9.1667	1.71E-05	2.391284051
V14411	SM d38:5	1.5517	0.00026794	2.073974926
V14186	SM(d18:0/20:2(11Z,14Z))	0.30456	0.0066605	1.683922703
V10462	SQMG(0:0/16:1(11Z))	0.60615	0.00020869	2.192904038
V702	Succinate semialdehyde	1.3538	0.0046626	1.646416649
V5192	Tetrahydro-11-deoxycortisol	0.87707	0.020227	1.434680079
V1370	Theophylline	4.3696	0.00023416	2.205341702
V1371	Theophylline	1.2291	0.026878	1.57959581
V2528	Thionazin	-2.4995	0.0072376	1.738829811
V6267	Thromboxane B2	-4.2707	0.0015361	1.998480748
V3939	Trp Val	0.51275	0.049538	1.160569105
V11584	α-N-Acetylneuraminyl-2,6-β-D-galactosyl-1,4-N-acetyl-β-D-glucosamine	-1.7578	0.011086	1.38472264

Table S9. A total of 82 different metabolites in stool samples before and after ground coffee consumption.

varID	Compounds	Count (up)	Count (down)	p(T test)	PLSDA_VIP
V125	2-Methylpyridine	5	17	0.0089318	2.225213463
V302	Phenethylamine	5	17	0.014625	2.185921647
V323	p-Anisidine	5	17	0.037524	1.958880699
V446	p-Phenetidine	1	13	0.013991	2.137242795
V946	1,3-Dimethyl-8-isoquinolinol	5	18	0.0083215	2.316927715
V1045	9-Fluorenon	4	18	0.010062	2.107271255
V1264	N-Phenylacetylglycine	2	17	0.017447	2.065006755
V1314	3,7-Dimethyluric acid	4	16	0.0045585	2.292043605
V1418	3-Indolebutyric acid	4	18	0.0015502	2.610989691
V1420	N2-Acetyl-L-aminoadipate	17	5	0.020781	1.922462607
V1655	3-Methylindolepyruvate	4	17	0.0069812	2.324451219
V1710	Dehydroxyzyleuton	5	18	0.0012189	2.629313248
V1971	5-Methoxytryptophan	4	17	0.0068522	2.198710465
V2055	Albendazole-2-aminosulfone	3	17	0.00086783	2.666081018
V2073	Dl-Cystine	4	16	0.011327	2.103576691
V2726	N-Benzoyl-4-methoxyanthranilate	3	16	0.00514	2.368965554
V2920	Asparenyol	2	14	0.0044727	2.331183369
V2945	Oleamide	15	3	0.046457	1.649860874
V3065	Etodolac (JP17/USP/INN)	4	17	0.017368	1.951416873
V3074	Tenofovir	3	18	0.0025268	2.456965095
V3940	1-Oxaspiro[2.5]octan-6-ol, 5-methoxy-4	1	15	0.036266	1.7705592
V4390	W146	4	17	0.0028207	2.44333753
V5725	Phytosphingosine-1-P	3	16	0.033998	1.796560706
V7080	Ceanothenic acid	4	16	0.0059796	2.242262181
V7171	5a,11a-Dehydrooxytetracycline	2	20	2.17E-05	3.320736335
V7878	1α;,25-dihydroxy-11α;-phenylvitamin D	5	17	0.0084342	2.175613582
V7958	Postin	4	16	0.021857	1.894722022
V8007	N-behenoyl-5-hydroxytryptamide	4	16	0.012972	2.047557506
V8383	Ginsenoyne A linoleate	5	18	0.0018076	2.549162937
V9242	α-Ergocryptine	2	18	0.0013338	2.572438373
V9378	Bilirubin	4	16	0.010378	2.097897583
V10120	PE-Cer(d16:2(4E,6E)/16:0)	17	5	0.011683	2.077846809
V10965	alpha-D-Glucopyranoside, 4-O-acetyl-6	17	5	0.049366	1.632766973
V404	4-Hydroxy-3-Methoxybenzyl alkohol	4	16	0.00153	2.107009886
V405	p-Anisaldehyde	4	17	0.0034686	2.093560663
V410	2-Amino-6-fluorobenzonitrile	2	15	0.02897	1.512312739
V549	Phenoxyacetic acid	2	14	0.0045166	1.932779156
V582	D-Histidine	15	2	0.041601	1.582560697
V739	1,4-Cyclohexanedicarboxylic acid	1	14	0.021382	1.712245896
V768	1H-Indole-3-Acetic Acid	4	17	0.0071417	1.789720796
V802	5,7-Dihydroxychromone	1	17	0.0024043	2.048298023
V839	L-Tyrosine	2	16	0.0042157	1.948701401
V849	1-Methyluric Acid	3	16	0.0049111	1.959386903
V863	4-Pyridoxic acid	3	15	0.0051923	1.975722463
V875	Fuberidazole	3	18	0.0085965	1.975997656
V897	5-Butyltetrahydro-2-oxo-3-furancarbox	5	17	0.038654	1.787958803
V958	5-HYDROXYINDOLEACETATE	4	16	0.010904	1.788787893
V960	Desmethyldeschlorobenzoyl Indometha	5	18	0.042801	1.775683855
V966	Sedoheptulose anhydride	4	18	0.011093	1.780644559
V1039	6-amino-5[N-methylformylamino]-1-m	2	20	0.00073012	2.261640763
V1041	Dl-4-Hydroxy-3-methoxymandelie acid	4	19	0.030628	1.875282109
V1044	5-Acetylamino-6-amino-3-methyluracil	2	21	1.40E-05	2.757492123
V1189	Danielone	3	18	0.002525	2.034673706
V1232	Bisnorbiotin	4	16	0.036695	1.654395986
V1298	Methyl 5-hydroxyoxindole-3-acetate	6	18	0.018922	1.746546263
V1346	Elenolide	2	19	0.018659	1.618013708
V1520	2-Amino-5-benzoyl-benzimidazole	3	18	0.025374	1.776389383
V1555	2-Hydroxyphenanzine-1-Carboxylic aci	5	17	0.025514	1.894681129
V1708	2-Methoxycanthin-6-one	4	17	0.023253	1.766032732
V1712	N-Feruloylglycine	4	17	0.025274	1.917712994
V1759	Acetaminophen cystein	5	17	0.012781	1.913507178
V1762	10-Hydroxycarbazepine	4	16	0.031962	1.731706332
V1810	LPE 2:0	3	17	0.0042337	2.066379478

V1945	Nevirapine	17	4	0.0089871	1.856588376
V1950	Dihydrocitrinone	4	19	0.0080735	2.076777842
V1985	2,3-Dihydroxycarbamazepine	5	17	0.010938	2.013282685
V2181	1-cyclopropyl-6,7-difluoro-1,4-dihydro-	4	17	0.001632	2.322854386
V2383	Colnelenic acid	2	17	0.00051047	2.366576419
V2448	1-iodo-2-methylundecane	4	17	0.0082237	2.020114094
V2630	Fluconazole (JP17/USAN/INN)	3	18	0.01629	1.926119167
V2771	Geranyl diphosphate	3	18	0.01505	1.684804648
V2924	N-((R)-Pantothenoyl)-L-cysteine	5	17	0.013415	1.862240355
V3344	3',4',5'-O-Trimethyltricetin	2	18	0.028491	1.807695305
V3811	3-O-Caffeoyl-4-O-methylquinic acid	2	17	0.043405	1.440058681
V4332	2'-Oxoaloesol 7-glucoside	2	16	0.0032681	2.013888668
V4461	Prednisone 21-acetate	4	17	0.0084607	1.791827796
V4720	α-Narcotine	4	17	0.0047769	2.025192218
V4743	1-(2H-1,3-Benzodioxol-5-yl)-2-[2,6-dir	4	19	3.15E-05	2.644882731
V5022	Sesartemin	3	18	0.0020683	2.05148759
V5115	Valsartan	4	18	0.0059257	2.000355361
V7002	Tetrahydroaldosterone-3-glucuronide	1	18	1.54E-05	2.795828282
V8486	Cer-AP t40:1	17	5	0.038	1.453750622

Table S10. Correlation analysis of metabolomics and T cell subsets in subjects after ground coffee consumption.

Metabolites	Cell	rho	pvalue	relation
(±)12,13-DiHOME	CD4+CD45RA+	-0.392460082	0.004817487	negtive
Nicardipine	CD4+CD45RA+	0.336174813	0.016990287	positive
Prednisolone 21-acetate	CD4+CD45RA+	0.327434268	0.020279312	positive
(22:5(7Z,10Z,13Z,16Z,1	CD4+CD45RA+	0.323544245	0.02190751	positive
ierPE 36:4e; PE(18:2e/18	CD4+CD45RA+	0.28882219	0.04193102	positive
8phiC9SPC	CD4+CD45RA+	0.282674993	0.046697968	positive
1-Pyrroline-2-carboxylate	CD4+CD45RA+	-0.281762519	0.04744179	negtive
baicalein	CD4+CD45RA+	0.27984152	0.049039272	positive
Prostaglandin D2	CD4+CD45RA+	0.272301599	0.055736973	positive
HFA 20:0; FAHFA 12:0/	CD4+CD45RA+	-0.242526115	0.089699987	negtive
1-Methylxanthine	CD4+CD45RA+	0.228646898	0.110238423	positive
Cer-NS d34:1	CD4+CD45RA+	0.207467885	0.148262038	positive
AS 1-5	CD4+CD45RA+	0.188257895	0.190447288	positive
HexCer-BS d34:2	CD4+CD45RA+	0.184271823	0.20017792	positive
ylamino-6-amino-3-meth	CD4+CD45RA+	0.183743548	0.201493424	positive
PE(16:1(9Z)/P-18:1(11Z)	CD4+CD45RA+	0.174138553	0.226483065	positive
Homomangiferin	CD4+CD45RA+	0.172457679	0.231067152	positive
)/22:6(4Z,7Z,10Z,13Z,16	CD4+CD45RA+	0.164533559	0.253535587	positive
Phenoxyacetic acid	CD4+CD45RA+	0.163236884	0.257347819	positive
Galactosylceramide	CD4+CD45RA+	0.157233763	0.275497364	positive
PE(16:0/22:6)	CD4+CD45RA+	0.150078042	0.29821118	positive
Sedoheptulose anhydride	CD4+CD45RA+	0.150030017	0.298367598	positive
DS d34:0; Cer-NDS d18:	CD4+CD45RA+	-0.149693842	0.299464008	negtive
3-Methylxanthine	CD4+CD45RA+	0.139272423	0.334741151	positive
1,7-Dimethylxanthine	CD4+CD45RA+	0.139128348	0.335246321	positive
3,7-Dimethyluric acid	CD4+CD45RA+	0.137495499	0.341004778	positive
N-Acetyl-L-methionine	CD4+CD45RA+	-0.135526475	0.348029834	negtive
Cer-NS d36:2	CD4+CD45RA+	0.134277825	0.352530566	positive
PI 38:4	CD4+CD45RA+	-0.10872854	0.45228353	negtive
-Hydroxyphenylethyleneg	CD4+CD45RA+	0.106567416	0.461373239	positive
Cer 36:1	CD4+CD45RA+	0.106279266	0.46259262	positive
PE 36:4	CD4+CD45RA+	0.102533318	0.478601921	positive
Cysteinyl-Tyrosine	CD4+CD45RA+	-0.099411695	0.492163561	negtive
2-Amino-5-phenylpyridin	CD4+CD45RA+	-0.09897947	0.494056926	negtive
Alverine (INN)	CD4+CD45RA+	-0.09830712	0.497009645	negtive
2,20-Dioxo-leukotriene B	CD4+CD45RA+	-0.097634771	0.499971444	negtive
5:0/20:5(5Z,8Z,11Z,14Z,	CD4+CD45RA+	0.094849322	0.512337684	positive
-Dihydroxyprostaglandin	CD4+CD45RA+	-0.087117302	0.547455648	negtive
Physalin L	CD4+CD45RA+	0.086877177	0.548564435	positive
Gluten exorphin B4	CD4+CD45RA+	0.085964702	0.552787592	positive
12(S)-HHT	CD4+CD45RA+	-0.08024973	0.579583229	negtive
Cascarillin	CD4+CD45RA+	-0.080201705	0.579810879	negtive
Albafuran A	CD4+CD45RA+	-0.071076961	0.623780691	negtive
PS 40:6	CD4+CD45RA+	0.069684236	0.6306129	positive
Glutamylvaline	CD4+CD45RA+	-0.058398368	0.687067021	negtive
15,16-DiHODE	CD4+CD45RA+	0.057341818	0.692445753	positive
xododecanoyl)homoserine	CD4+CD45RA+	-0.055228719	0.703248159	negtive
PA 20:3; PA 2:0-18:3	CD4+CD45RA+	-0.052827471	0.715594311	negtive
PE 38:4	CD4+CD45RA+	0.051674871	0.721546378	positive
Nilotinib (USAN/INN)	CD4+CD45RA+	0.050954497	0.725274755	positive
PE 22:5	CD4+CD45RA+	0.050618322	0.727016831	positive
Aflatoxin ExB2	CD4+CD45RA+	0.049561772	0.732500793	positive
PA 20:3	CD4+CD45RA+	-0.046103974	0.750539658	negtive
DGDG 10:0	CD4+CD45RA+	0.043222476	0.765674065	positive
Achimilic acid	CD4+CD45RA+	0.038083804	0.792876483	positive
PS 38:4	CD4+CD45RA+	-0.033473406	0.817492945	negtive
PA 41:5	CD4+CD45RA+	-0.032897107	0.820582819	negtive
PS 36:4	CD4+CD45RA+	-0.030880058	0.831418333	negtive
Cer 34:1	CD4+CD45RA+	0.028814984	0.842544059	positive
Nap-Thr-OH	CD4+CD45RA+	0.027182135	0.851362856	positive
11,12-EET	CD4+CD45RA+	-0.023196062	0.872964731	negtive
G1Cer 40:2	CD4+CD45RA+	0.022715812	0.87557389	positive

CL 74:5	CD4+CD45RA+	-0.020266539	0.888900409	negtive
PE 34:2	CD4+CD45RA+	0.01805739	0.900946649	positive
Theophylline	CD4+CD45RA+	-0.012390443	0.93194269	negtive
(±)-Abscisic acid	CD4+CD45RA+	0.005474847	0.969899682	positive
PE 36:2	CD4+CD45RA+	-0.004082123	0.97755449	negtive
(22:5(7Z,10Z,13Z,16Z,1(	Tfh-like	-0.507803121	0.000204143	negtive
Prednisolone 21-acetate	Tfh-like	-0.402641056	0.003995743	negtive
1-Pyrroline-2-carboxylate	Tfh-like	0.399663866	0.004294271	positive
Nicardipine	Tfh-like	-0.388043217	0.005656414	negtive
Cer-NS d34:1	Tfh-like	-0.379879952	0.006827633	negtive
ıerPE 36:4e; PE(18:2e/18	Tfh-like	-0.369123649	0.008691674	negtive
AS 1-5	Tfh-like	-0.359711885	0.01067153	negtive
Galactosylceramide	Tfh-like	-0.346938776	0.01397612	negtive
Prostaglandin D2	Tfh-like	-0.340888355	0.015826716	negtive
?E(16:1(9Z)/P-18:1(11Z)	Tfh-like	-0.309195678	0.029319194	negtive
baicalein	Tfh-like	-0.304777911	0.031806707	negtive
PE 36:4	Tfh-like	-0.301416567	0.033815501	negtive
5:0/20:5(5Z,8Z,11Z,14Z,	Tfh-like	-0.285090036	0.045136032	negtive
Phenoxyacetic acid	Tfh-like	-0.282496999	0.04719247	negtive
)/22:6(4Z,7Z,10Z,13Z,16	Tfh-like	-0.273853541	0.054609124	negtive
PE(16:0/22:6)	Tfh-like	-0.273661465	0.054784129	negtive
Homomangiferin	Tfh-like	-0.269627851	0.058565828	negtive
Cysteinyl-Tyrosine	Tfh-like	0.263193277	0.06503389	positive
PS 40:6	Tfh-like	-0.260984394	0.067382441	negtive
(±)12,13-DiHOME	Tfh-like	0.257527011	0.071194647	positive
ylamino-6-amino-3-meth	Tfh-like	-0.247346939	0.083428521	negtive
PE 38:4	Tfh-like	-0.242160864	0.090269877	negtive
3,7-Dimethyluric acid	Tfh-like	-0.241392557	0.091319833	negtive
Glutamylvaline	Tfh-like	0.22967587	0.108546789	positive
Cer-NS d36:2	Tfh-like	-0.229579832	0.108697693	negtive
8phiC9SPC	Tfh-like	-0.22487395	0.116292262	negtive
Sedoheptulose anhydride	Tfh-like	-0.207490996	0.147904018	negtive
HFA 20:0; FAHFA 12:0/	Tfh-like	0.207010804	0.148860159	positive
PE 34:2	Tfh-like	-0.189435774	0.187101794	negtive
1,7-Dimethylxanthine	Tfh-like	-0.188091236	0.190294783	negtive
HexCer-BS d34:2	Tfh-like	-0.183961585	0.200346327	negtive
Cer 36:1	Tfh-like	-0.180792317	0.208312828	negtive
Cascarillin	Tfh-like	0.178007203	0.215496565	positive
Achimilic acid	Tfh-like	-0.177527011	0.216752535	negtive
N-Acetyl-L-methionine	Tfh-like	0.172340936	0.230645356	positive
-Hydroxyphenylethyleneg	Tfh-like	-0.165042017	0.251226362	negtive
1-Methylxanthine	Tfh-like	-0.161968788	0.260255269	negtive
PE 36:2	Tfh-like	-0.156302521	0.277471017	negtive
CL 74:5	Tfh-like	-0.155246098	0.280762585	negtive
Nap-Thr-OH	Tfh-like	0.128835534	0.371438193	positive
PS 36:4	Tfh-like	-0.125954382	0.382300082	negtive
PI 38:4	Tfh-like	0.125378151	0.384495053	positive
PS 38:4	Tfh-like	-0.120384154	0.403831594	negtive
3-Methylxanthine	Tfh-like	-0.115390156	0.423724994	negtive
Alverine (INN)	Tfh-like	0.110588235	0.443370462	positive
Physalin L	Tfh-like	0.108955582	0.450163858	positive
Cer 34:1	Tfh-like	-0.100312125	0.487071985	negtive
PE 22:5	Tfh-like	-0.098871549	0.493374727	negtive
Aflatoxin ExB2	Tfh-like	0.089459784	0.535579453	positive
G1Cer 40:2	Tfh-like	-0.078511405	0.586809285	negtive
PA 20:3	Tfh-like	0.078223289	0.588186833	positive
DS d34:0; Cer-NDS d18:	Tfh-like	0.077166867	0.593250286	positive
12(S)-HHT	Tfh-like	0.072364946	0.616507616	positive
Theophylline	Tfh-like	-0.071980792	0.618384964	negtive
DGDG 10:0	Tfh-like	0.06909964	0.632542025	positive
ꝁododecanoyl)homoserine	Tfh-like	0.066218487	0.646831824	positive
PA 20:3; PA 2:0-18:3	Tfh-like	0.062184874	0.667052124	positive
2,20-Dioxo-leukotriene B	Tfh-like	0.060552221	0.675305194	positive
PA 41:5	Tfh-like	0.058247299	0.687021588	positive

Albafuran A	Tfh-like	0.057959184	0.688491383	positive
-Dihydroxyprostaglandin	Tfh-like	0.054597839	0.705722657	positive
2-Amino-5-phenylpyridin	Tfh-like	0.042785114	0.76740351	positive
15,16-DiHODE	Tfh-like	-0.042304922	0.769944575	negtive
(±)-Abscisic acid	Tfh-like	0.028187275	0.845622102	positive
11,12-EET	Tfh-like	0.018487395	0.898482134	positive
Nilotinib (USAN/INN)	Tfh-like	0.012917167	0.929052797	positive
Gluten exorphin B4	Tfh-like	0.0084994	0.953373627	positive
Prednisolone 21-acetate	Th1	-0.659158589	1.9407E-07	negtive
Prostaglandin D2	Th1	-0.591537803	6.07328E-06	negtive
Sedoheptulose anhydride	Th1	-0.572807613	1.38013E-05	negtive
3,7-Dimethyluric acid	Th1	-0.557727409	2.57907E-05	negtive
Phenoxyacetic acid	Th1	-0.548506393	3.72487E-05	negtive
Nicardipine	Th1	-0.497550673	0.000236725	negtive
3-Methylxanthine	Th1	-0.487032951	0.000334802	negtive
ylamino-6-amino-3-meth	Th1	-0.453702819	0.000935767	negtive
Homomangiferin	Th1	-0.435933152	0.001553662	negtive
Cascarillin	Th1	0.429161468	0.001871578	positive
baicalein	Th1	-0.427096345	0.001979421	negtive
15,16-DiHODE	Th1	-0.4198444	0.002403185	negtive
Cer-NS d34:1	Th1	-0.416770728	0.002605815	negtive
1,7-Dimethylxanthine	Th1	-0.414609552	0.002757202	negtive
(22:5(7Z,10Z,13Z,16Z,1	Th1	-0.401594472	0.003844441	negtive
-Hydroxyphenylethylene	Th1	-0.3996254	0.004038175	negtive
8phiC9SPC	Th1	-0.386850451	0.005516585	negtive
1-Pyrroline-2-carboxylat	Th1	0.3729709	0.007639324	positive
1-Methylxanthine	Th1	-0.363653831	0.009433591	negtive
Galactosylceramide	Th1	-0.363557779	0.009453837	negtive
Achimilic acid	Th1	-0.359379506	0.010371655	negtive
AS 1-5	Th1	-0.357266357	0.010864378	negtive
PS 40:6	Th1	-0.341321683	0.015274469	negtive
Theophylline	Th1	-0.339688794	0.015802068	negtive
Cysteinyl-Tyrosine	Th1	0.335174338	0.017342159	positive
Nap-Thr-OH	Th1	0.33291711	0.018158824	positive
(±)12,13-DiHOME	Th1	0.323936225	0.021738607	positive
HFA 20:0; FAHFA 12:0/	Th1	0.306502741	0.030396369	positive
PE 36:4	Th1	-0.298866587	0.035004588	negtive
11,12-EET	Th1	0.295024496	0.037533051	positive
Glutamylvaline	Th1	0.29199885	0.039628495	positive
Aflatoxin ExB2	Th1	0.288733074	0.041997161	positive
Physalin L	Th1	0.25084046	0.07890763	positive
PE 34:2	Th1	-0.250552303	0.079263713	negtive
DGDG 10:0	Th1	0.247958892	0.082525683	positive
Cer 36:1	Th1	-0.24786284	0.08264849	negtive
Alverine (INN)	Th1	0.239698398	0.093621781	positive
herPE 36:4e; PE(18:2e/18	Th1	-0.238881954	0.094778541	negtive
Nilotinib (USAN/INN)	Th1	0.237585249	0.096638501	positive
Cer 34:1	Th1	-0.235760256	0.099303964	negtive
PE 38:4	Th1	-0.234031315	0.101881198	negtive
PE(16:1(9Z)/P-18:1(11Z)	Th1	-0.233839211	0.10217071	negtive
CL 74:5	Th1	-0.233262897	0.103043052	negtive
G1Cer 40:2	Th1	-0.231101722	0.106365483	negtive
PS 38:4	Th1	-0.229372781	0.109082144	negtive
12(S)-HHT	Th1	0.228076076	0.111154269	positive
PS 36:4	Th1	-0.227547788	0.112007041	negtive
Gluten exorphin B4	Th1	0.225818848	0.114832889	positive
Albafuran A	Th1	0.22529056	0.115707085	positive
PA 20:3; PA 2:0-18:3	Th1	0.222553071	0.120318354	positive
N-Acetyl-L-methionine	Th1	0.219047164	0.126425833	positive
Cer-NS d36:2	Th1	-0.21227548	0.138880002	negtive
2,20-Dioxo-leukotriene B	Th1	0.204063012	0.15518475	positive
5:0/20:5(5Z,8Z,11Z,14Z,	Th1	-0.199452504	0.164933556	negtive
PE 36:2	Th1	-0.197099224	0.170078221	negtive
PA 20:3	Th1	0.196811067	0.170716094	positive

PE(16:0/22:6)	Th1	-0.188022286	0.191012892	negtive
2-Amino-5-phenylpyridin	Th1	0.17284603	0.230002413	positive
-Dihydroxyprostaglandin	Th1	0.167082895	0.246152011	positive
)/22:6(4Z,7Z,10Z,13Z,16	Th1	-0.160791472	0.264641692	negtive
PI 38:4	Th1	0.144606668	0.3163727	positive
PE 22:5	Th1	0.133032371	0.357055134	positive
DS d34:0; Cer-NDS d18:	Th1	-0.127509366	0.377543096	negtive
HexCer-BS d34:2	Th1	-0.122994911	0.394799572	negtive
xododecanoyl)homoserine	Th1	0.108587072	0.452875528	positive
PA 41:5	Th1	0.085486506	0.555006953	positive
(±)-Abscisic acid	Th1	0.081068102	0.575710197	positive
PS 36:4	Treg	0.482024939	0.000393354	positive
PS 38:4	Treg	0.475637003	0.000481424	positive
PE 34:2	Treg	0.466799559	0.000632614	positive
Cer-NS d34:1	Treg	0.464302021	0.000682474	positive
Homomangiferin	Treg	0.444946097	0.001205456	positive
PE 36:2	Treg	0.432842641	0.001692236	positive
CL 74:5	Treg	0.417184996	0.002577651	positive
DGDG 10:0	Treg	-0.416992877	0.002590679	negtive
PS 40:6	Treg	0.407915285	0.003276646	positive
PE 36:4	Treg	0.39686848	0.004323821	positive
Glutamylvaline	Treg	-0.37597561	0.007127706	negtive
PA 41:5	Treg	-0.368675112	0.008426	negtive
N-Acetyl-L-methionine	Treg	-0.362431266	0.009694107	negtive
Aflatoxin ExB2	Treg	-0.362047029	0.009777256	negtive
Cascarillin	Treg	-0.360894319	0.010030405	negtive
(22:5(7Z,10Z,13Z,16Z,1	Treg	0.355370917	0.011323342	positive
Nilotinib (USAN/INN)	Treg	-0.347157857	0.013509703	negtive
PE 38:4	Treg	0.345284703	0.014056059	positive
Achimilic acid	Treg	0.308782217	0.029122871	positive
Gluten exorphin B4	Treg	-0.308590099	0.02922846	negtive
AS 1-5	Treg	0.301625809	0.033277006	positive
Physalin L	Treg	-0.300136891	0.034200244	negtive
11,12-EET	Treg	-0.282462003	0.046870735	negtive
5:0/20:5(5Z,8Z,11Z,14Z,	Treg	0.282462003	0.046870735	positive
15,16-DiHODE	Treg	0.277418897	0.051115935	positive
xododecanoyl)homoserine	Treg	0.276218157	0.052171318	positive
ierPE 36:4e; PE(18:2e/18	Treg	0.274248944	0.053940287	positive
Galactosylceramide	Treg	0.271703376	0.056298504	positive
Cer 34:1	Treg	0.268341305	0.059539751	positive
Nap-Thr-OH	Treg	-0.268245245	0.059634515	negtive
PA 20:3; PA 2:0-18:3	Treg	-0.250570357	0.079241366	negtive
-Dihydroxyprostaglandin	Treg	-0.247256316	0.083427272	negtive
Theophylline	Treg	0.244278481	0.087334654	positive
Sedoheptulose anhydride	Treg	0.242981682	0.089080315	positive
ylamino-6-amino-3-methy	Treg	0.242501387	0.089733719	positive
PE(16:1(9Z)/P-18:1(11Z)	Treg	0.241732913	0.09078693	positive
Prednisolone 21-acetate	Treg	0.236113451	0.098783727	positive
3,7-Dimethyluric acid	Treg	0.23510483	0.100274976	positive
)/22:6(4Z,7Z,10Z,13Z,16	Treg	0.231166404	0.106264867	positive
PA 20:3	Treg	-0.226651623	0.113465065	negtive
Albafuran A	Treg	-0.225691031	0.115043934	negtive
Phenoxyacetic acid	Treg	0.217093735	0.129928681	positive
DS d34:0; Cer-NDS d18:	Treg	0.21526861	0.133266909	positive
8phiC9SPC	Treg	0.208256291	0.146692156	positive
(±)-Abscisic acid	Treg	-0.20249274	0.158456276	negtive
HexCer-BS d34:2	Treg	0.195000125	0.174764614	positive
PE(16:0/22:6)	Treg	0.184241497	0.200253271	positive
1-Methylxanthine	Treg	0.157969313	0.2732292	positive
Cer 36:1	Treg	0.151869555	0.292414101	positive
Alverine (INN)	Treg	-0.149996401	0.298477115	negtive
Cer-NS d36:2	Treg	0.149948372	0.298633638	positive
Prostaglandin D2	Treg	0.147162656	0.30780275	positive
3-Methylxanthine	Treg	0.12458875	0.388655086	positive

1-Pyrroline-2-carboxylate	Treg	0.123099832	0.394393342	positive
Cysteinyl-Tyrosine	Treg	-0.110131844	0.446434111	negtive
12(S)-HHT	Treg	-0.104416323	0.470518003	negtive
2,20-Dioxo-leukotriene B	Treg	-0.09774021	0.499506369	negtive
baicalein	Treg	0.090535772	0.531788179	positive
(±)12,13-DiHOME	Treg	-0.087990204	0.54343405	negtive
1,7-Dimethylxanthine	Treg	0.072524677	0.616711787	positive
PI 38:4	Treg	0.067817777	0.639817166	positive
-Hydroxyphenylethyleneg	Treg	0.054225404	0.708397763	positive
HFA 20:0; FAHFA 12:0/	Treg	0.053168753	0.713835104	positive
PE 22:5	Treg	-0.050815303	0.725995896	negtive
2-Amino-5-phenylpyridin	Treg	-0.044379339	0.759587145	negtive
G1Cer 40:2	Treg	0.038855936	0.788772586	positive
Nicardipine	Treg	0.011863308	0.934831716	positive
